# Endothelial senescence mediates hypoxia-induced vascular remodeling by modulating PDGFB expression

**DOI:** 10.3389/fmed.2022.908639

**Published:** 2022-09-20

**Authors:** Priscilla Kyi, Kathryn Hendee, Tendai Hunyenyiwa, Kienna Matus, Tadanori Mammoto, Akiko Mammoto

**Affiliations:** ^1^Department of Pediatrics, Medical College of Wisconsin, Milwaukee, WI, United States; ^2^Department of Cell Biology, Neurobiology and Anatomy, Medical College of Wisconsin, Milwaukee, WI, United States; ^3^Department of Pharmacology and Toxicology, Medical College of Wisconsin, Milwaukee, WI, United States

**Keywords:** pulmonary hypertension, hypoxia, endothelial cell, senescence, TWIST1, PDGFB

## Abstract

Uncontrolled accumulation of pulmonary artery smooth muscle cells (PASMCs) to the distal pulmonary arterioles (PAs) is one of the major characteristics of pulmonary hypertension (PH). Cellular senescence contributes to aging and lung diseases associated with PH and links to PH progression. However, the mechanism by which cellular senescence controls vascular remodeling in PH is not fully understood. The levels of senescence marker, p16^INK4A^ and senescence-associated β-galactosidase (SA-β-gal) activity are higher in PA endothelial cells (ECs) isolated from idiopathic pulmonary arterial hypertension (IPAH) patients compared to those from healthy individuals. Hypoxia-induced accumulation of α-smooth muscle actin (αSMA)-positive cells to the PAs is attenuated in *p16*^*fl*/*fl*^*-Cdh5(PAC)-Cre*^*ERT*2^ (*p16*^*iΔEC*^) mice after tamoxifen induction. We have reported that endothelial TWIST1 mediates hypoxia-induced vascular remodeling by increasing platelet-derived growth factor (PDGFB) expression. Transcriptomic analyses of IPAH patient lungs or hypoxia-induced mouse lung ECs reveal the alteration of senescence-related gene expression and their interaction with TWIST1. Knockdown of p16^INK4A^ attenuates the expression of PDGFB and TWIST1 in IPAH patient PAECs or hypoxia-treated mouse lungs and suppresses accumulation of αSMA–positive cells to the supplemented ECs in the gel implanted on the mouse lungs. Hypoxia-treated mouse lung EC-derived exosomes stimulate DNA synthesis and migration of PASMCs *in vitro* and in the gel implanted on the mouse lungs, while *p16*^*iΔEC*^ mouse lung EC-derived exosomes inhibit the effects. These results suggest that endothelial senescence modulates TWIST1-PDGFB signaling and controls vascular remodeling in PH.

## Introduction

PH is a cardiopulmonary disorder characterized by a sustained elevation of PA pressure, resulting in right-side heart failure and eventual death (1–4). Remodeling of distal PAs is a key feature of PH and involves marked accumulation of PASMCs to normally non-muscularized distal PAs, which narrows and blocks the PAs, increasing PA pressure (5, 6). ECs secrete angiocrine factors and regulate various physiological functions (7, 8). Disrupted PAEC signaling and dysfunctional secretion of angiocrine factors stimulate PASMC proliferation and accumulation to distal PAs (3, 9–11), highlighting EC dysfunction as a critical contributor to PH pathology.

Accumulation of senescent cells, the cells that irreversibly lose the ability to proliferate (12), promotes aging and exacerbates age-related pathologies (12–18), including chronic lung disease (19–21) and cancer (22–24). Although senescent cells are unable to replicate, they secrete senescence-associated secretory phenotype (SASP) factors such as inflammatory cytokines, chemokines, growth factors, and proteases (13, 18, 25–28), which allow the cells to be metabolically active. Senescent ECs play a key role in vascular aging and age-related cardiovascular and degenerative diseases (12, 13, 15), and senolytic reagents are extensively studied in the aging research (28, 29). Cellular senescence also contributes to idiopathic pulmonary fibrosis (IPF) and chronic obstructive pulmonary disease (COPD) (19–21) associated with PH (30, 31). Recently, it is reported that EC senescence is involved in PH (32, 33), however the underlying mechanism is not fully understood.

TWIST1 is a bHLH family transcription factor and contributes to chronic lung diseases associated with PH such as IPF (31, 34, 35). We have reported that TWIST1 is upregulated in IPAH patients-derived PAECs and mediates hypoxia-induced increases in right ventricular systolic pressure (RVSP) and accumulation of PASMCs to PAs (11, 36). TWIST1 contributes to age-dependent inhibition of angiogenesis and lung regeneration (37) and is involved in cellular senescence to promote tumor cell proliferation (38), suggesting that endothelial TWIST1 and senescence may contribute to vascular remodeling in PH.

Exosomes are one of the types of extracellular vesicles with size typically from 30 to 150 nm in diameter and contain various proteins, lipids, and nucleic acids (DNA, mRNA, miRNA, non-coding RNA) (39–41). Exosomes are produced by ECs and other cell types, and serve as a messenger of signals for cell-cell communications (39–43). Exosomes also remove unused or harmful molecules and proteins (39, 40, 42, 44, 45) to maintain tissue homeostasis in normal physiology and contribute to disease pathology [e.g., aging (46), cancer (45), atherosclerosis (47)]. SASP factors such as inflammatory cytokines, membrane organization and signaling proteins (48, 49) are enriched in exosomes from senescence cells (50), which constitutes part of the SASP and mediates paracrine effects on the microenvironment (45, 51, 52). It has been reported that human mesenchymal stem cell (MSC)-derived exosomes suppress PH and other lung diseases such as bronchopulmonary dysplasia (BPD), airway inflammation, and pulmonary fibrosis in animal models (53–61). We have demonstrated that exosomes collected from ECs promote angiogenesis (41). However, the role of EC-derived exosomes in PH pathology has not been well characterized.

Here we have demonstrated that EC senescence modulates TWIST1-PDGFB signaling and mediates hypoxia-induced αSMA-positive cell accumulation to PAs in the mouse lungs. Inhibition of EC senescence suppresses accumulation of αSMA-positive cells to IPAH patient lung ECs and exosomes collected from p16^INK4A^ knocked down ECs inhibit hypoxia-induced αSMA-positive cell recruitment in the gel implanted on the mouse lungs. Understanding the mechanism by which EC senescence mediates vascular remodeling in PH will lead to the development of novel therapeutics to manage PH and exosomes derived from ECs, in which cellular senescence is modulated, could be one of the sound strategies to prevent PH.

## Materials and methods

### Materials

Anti-p16^INK4A^, -TWIST1, -PDGFB, -VE-cadherin, -SLUG, -ERG, and -αSMA antibodies were purchased from Abcam (Cambridge, MA). Anti-p21 and -Flotillin-1 antibodies were from Cell Signaling (Danvers, MA). Anti-GM130 antibody was from BD Biosciences (Franklin Lakes, NJ). Anti-CD63 antibody was from Santa Cruze Biotechnology (Dallas, TX). Phospho-gamma-H2AX (Ser139) antibody was from Thermo Fisher Scientific (Waltham, MA). β-actin antibody was from Sigma (Burlington, MA). ABT-263 was purchased from Selleckchem (Houston, TX). Human pulmonary artery smooth muscle cells (HPASMCs) were purchased from Lonza and cultured in DMEM containing 5% FBS.

De-identified human IPAH patient ECs were obtained from unused donor control lungs at time of transplantation *via* the Pulmonary Hypertension Breakthrough Initiative (PHBI) Network, which is funded by the Cardiovascular Medical and Education Fund (CMREF) and NIH-NHLBI. The study using these de-identified human cells has been determined and approved as Non-Human Subjects Research by the Medical College of Wisconsin Institutional Review Board (IRB PRO00029154). We obtained ECs isolated from PA (>5 mm in diameter) from females and males (5 control samples; 45.6 ± 2.6 years old, 5 IPAH samples; 34.4 ± 2.5 years old). The patient demographic information is in Table 1. These ECs were cultured in ECM medium containing 5% FBS and growth factors (VEGF, bFGF and PDGF, Science Cell, Carlsbad, CA).

**Table 1:** Sample demographics.

**ID**	**Age**	**Sex**	**Race**
Con-1	36	Female	White
Con-2	45	Female	White
Con-3	47	Male	White
Con-4	49	Female	White
Con-5	51	Male	White
PAH-1	27	Female	White
PAH-2	32	Male	White
PAH-3	33	Female	White
PAH-4	40	Male	White
PAH-5	40	Female	White

### Plasmid construction and gene knockdown

ON-TARGET plus human p16^INK4A^ siRNA SMARTPool was purchased from Horizon Discovery (Lafayette, CO). As a control, siRNA with irrelevant sequences was used (36, 41). Lentiviral construct targeting human p16^INK4A^ (p16^INK4A^ shRNA, CCGGAGTAACCATGCCCGCATAGATCTCGAGATCTATGCGGGCATGGTTACTTTTTTG) was obtained from Sigma. As a control, plasmid with vector only was used. Generation of lentiviral vectors was accomplished by a five-plasmid transfection procedure as reported (36, 41, 62). Viral supernatants were collected starting 48 h after transfection, for four consecutive times every 12 h, pooled, and filtered through a 0.45 μm filter. Viral supernatants were then concentrated 100-fold by ultracentrifugation in a Beckman centrifuge for 1.5 h at 16,500 rpm. PAECs were incubated with viral stocks in the presence of 5 μg/ml polybrene (Sigma) and 90–100% infection was achieved 3 days later (41, 62).

### Molecular biological and biochemical methods

Quantitative reverse transcription (qRT)-PCR was performed with the iScript reverse transcription and iTaq SYBR Green qPCR kit (BioRad, Hercules, CA) using the BioRad real time PCR system (BioRad). β2 microglobulin and cyclophilin controlled for overall cDNA content as a reference gene. The primers used for human β2 microglobulin, human TWIST1, mouse Twist1, and mouse cyclophilin were previously described (35, 36, 62, 63). Human p16^INK4A^ primers, forward; GATCCAGGTGGGTAGAAGGTC, reverse; CCCCTGCAAACTTCGTCCT, human p21 primers, forward; TGTCCGTCAGAACCCATGC, reverse; AAAGTCGAAGTTCCATCGCTC, mouse p16^INK4A^ primers, forward; CGCAGGTTCTTGGTCACTGT, reverse; TGTTCACGAAAGCCAGAGCG, mouse αSMA primers, forward; CCCAGACATCAGGGAGTAATGG, reverse; TCTATCGGATACTTCAGCGTCA, and mouse Pdgfb primers forward; CATCCGCTCCTTTGATCTT, reverse; GTGCTCGGGTCATGTTCAAGT. The protein levels of human PDGFB were measured using ELISA (R&D systems, Minneapolis, MN) and normalized by the protein levels of total cell lysate. Senescence β-galactosidase activity was measured using SA β-gal staining kit (Cell Signaling).

### Mouse hypoxic exposure model *in vivo*

The *in vivo* animal study was carried out in strict accordance with the recommendations in the Guide for the Care and Use of Laboratory Animals of the National Institutes of Health. The protocols were reviewed and approved by the Animal Care and Use Committee of Medical College of Wisconsin. *p16*^*fl*/*fl*^ mice (B6;129S4-*Cdkn2a*^*tm*2.1*Nesh*^/Mmnc, 043540-UNC, MMRRC) (64) were crossed with *Cdh5(PAC)-Cre*^*ERT*2^ mice [obtained from Dr. Ralf Adams (65)] to develop *p16*^*iΔEC*^ mice. Eight to Ten-week old male *p16*^*iΔEC*^, *p16*^*fl*/*fl*^, and *Cdh5(PAC)-Cre*^*ERT*2^ mice as a control were treated with tamoxifen (125 μg/mouse, 5 days), housed in plexiglass chambers, and exposed to 8.5 ± 0.5% O_2_ for 3 weeks. After 3 weeks of exposure, right ventricular systolic pressure (RVSP) was measured (36); after IP injection of ketamine/xylazine, right jugular vein was exposed and a pressure transducer catheter (Millar Instruments, Houston, TX) was inserted into the jugular vein *via* a minimal incision. The catheter was advanced into the right ventricle and the position of the catheter was confirmed by the ventricular wave form. RVSP measurements were recorded and analyzed using a Quad Bridge Amplifier connected to a Power Lab device (AD Instruments, Colorado Springs, CO). We also measured Fulton's index; hearts and pulmonary vasculature were perfused *in situ* with cold 1X PBS injection into the right ventricle (RV); hearts were excised and used for Fulton's Index measurements (ratio of RV weight over left ventricle (LV) plus septal (S) weight, RV/[LV + S]) (36). Both ventricles were weighed first, then the right ventricular free wall was dissected and the remaining LV and ventricular septum was weighed. For pulmonary histological analysis, lungs were inflated by tracheotomy and perfused with 4% paraformaldehyde (PFA), excised, and fixed in 4% PFA overnight at 4°C followed by OCT embedding and cryosectioning.

### Mouse EC isolation

Mouse lung ECs were isolated from tamoxifen-induced *p16*^*iΔEC*^ or *p16*^*fl*/*fl*^ mouse lungs using anti-CD31 conjugated magnetic beads (41, 66). We cut lung tissue from *p16*^*iΔEC*^ or *p16*^*fl*/*fl*^ mouse into small pieces using small scissors and treated the tissue with collagenase A (5 ml, 1 mg/ml) for 30 min at 37°C. The tissue suspension was filtered through a 40 μm cell strainer (Falcon) to remove the undigested cell clumps and separate single cells. Cells were centrifuged (1,000 rpm, 5 min) at room temperature (RT) and the pellet was resuspended into 0.5 ml RBC Lysis Buffer (sigma, 1 min, RT). The lysis reaction was stopped by adding 10 ml 10% FBS/DMEM, centrifuged (1,000 rpm, 5 min, RT), and the pellet was resuspended into 0.5 ml 4% FBS/PBS with APC anti-mouse CD31 (Biolegend, 1/100), incubated (20 min, on ice) and washed three times with 4% FBS/PBS. Cells were centrifuged (1,000 rpm, 5 min, RT) and resuspended into 0.1 ml 4% FBS/PBS with anti-APC conjugated microbeads (Miltenyl Biotec, Somerville, MA), incubated (10 min, on ice) and washed three times with 4% FBS/PBS. The cells were then resuspended in 0.5 ml 4% FBS/PBS and CD31-positive ECs were magnetically separated using MACS column (Miltenyi Biotec) according to manufacturer's instruction. To increase the purity of the magnetically separated fraction, the eluted fraction was enriched over a second new MACS column. Using this method, we obtained 5 × 10^5^ cells/mouse and FACS analysis confirmed that more than 80% of the cells are CD31^+^ and VE-cadherin^+^ cells [not shown (41, 66)].

### Fibrin gel implantation on the mouse lung

Fibrin gel was fabricated as described (35, 36, 41, 66, 67). Briefly, we added thrombin (2.5 U/ml) to the fibrinogen solution (12.5 mg/ml), mixed well, supplemented the gel with human ECs labeled with GFP using lentiviral transduction (1 × 10^6^ cells), incubated the mixture at 37°C for 30 min until they solidified, and implanted on non-obese diabetic/severe combined immunodeficiency gamma (NSG, Jackson Laboratories, stock # 005557) mouse lungs. We also supplemented the gel with exosomes (5 μg/gel) isolated from tamoxifen-induced and hypoxia-treated *p16*^*iΔEC*^ or *p16*^*fl*/*fl*^ mouse lung ECs, incubated the mixture at 37°C for 30 min until they solidified, and implanted on *p16*^*fl*/*fl*^ mouse lungs. We used NSG mice for implantation of gel supplemented with human ECs to enhance engraftment of cells in the gel. In case of implantation of gel containing exosomes isolated from *p16*^*fl*/*fl*^ or *p16*^*iΔEC*^ mouse lung ECs, we used a syngeneic mouse implantation model, in which gel was implanted on the mouse of the same genetic background (*p16*^*fl*/*fl*^). Since the syngeneic mice retain intact immune systems, we selected this model rather than the immunocompromised NSG mouse model when we don't implant human cells. It is known that the NOD genetic background eliminates hemolytic complement and reduces dendritic cell and macrophage functions to inhibit immune system. NOD mice also express a unique variant of the Sirp-alpha protein to superior human cell engraftment. To date, NSG mouse line is one of the most standard mouse lines for human cell engraftment and therefore, we have been using NSG mice for our human cell implantation (11, 36, 37, 68–70). It is important to note that diabetes in NOD mice results from an autoimmune response, in which endogenous T cells attack and destroy beta cells in the pancreas, and therefore immune-deficient NOD-*scid* mice that lack these T cells do not become diabetic. For gel implantation on the mouse lungs, NSG or *p16*^*fl*/*fl*^ mice were mechanically ventilated and thoracotomy was performed in the fifth left intercostal space (35, 36, 41, 66, 67). After thoracotomy, a small area of the left visceral pleura (0.5 mm^2^) was scraped using forceps and the fabricated fibrin gel was implanted on the mouse lung surface using fibrin glue. For histological analysis, gels were fixed in 4% PFA overnight at 4°C followed by OCT embedding and cryosectioning. Fluorescent images were taken on a Nikon A1 confocal imaging system. Fluorescently labeled supplemented EC-derived vascular structures and accumulation of αSMA-positive cells in the gel were evaluated in five different areas of the gel using ImageJ software (35, 36, 41, 62, 66, 67).

### Microarray data analysis

Publicly available microarray datasets from 6 IPAH patient lungs and 11 healthy adult human frozen lung tissues (NCBI GEO, GSE113439) were utilized, and differential gene expression analysis was performed by GEO2R. The total number of genes identified by the array was 33,297, with 19,919 being downregulated and 13,378 upregulated. Of these, 790 downregulated and 1,285 upregulated genes possessed adjusted *p*-values < 0.001 following Benjamini and Hochberg false discovery rate multiple-testing correction of *p*-values, resulting in a total of 2,075 significantly differentially expressed genes. The 2,075 significantly differentially expressed genes underwent Biological Processes Gene Ontology (BP GO) Term analysis through the Functional Annotation Chart tool of the Database for Annotation, Visualization, and Integrated Discovery (DAVID) software, v6.8, and total 235 BP GO term categories were identified (Supplementary Table 1). Among these total 235 GO Term categories, 113 BP GO Term categories contain genes related to SASP/senescence and classified into transcription/gene expression, protein/RNA processing, cell cycle, inflammatory/immune response, cell signaling/signal transduction, or miscellaneous groups (Supplementary Table 2). To select GO Terms linked to Senescence/SASP, we used the master list curated from the literature [SASP Atlas (71), CellAge: the Database of cell senescence Genes https://genomics.senescence.info/cells/query.php]. Although the senescence/SASP genes were originally identified using biased analysis including the antibody arrays which selectively measure the secretion of pro-inflammatory cytokines, proteases and protease inhibitors, and growth factors, a recent comprehensive proteomic analysis demonstrated that SASP is more dynamic and heterogeneous dependent on cell types and senescence inducers. Therefore, we utilized unbiased genomic and proteomic database [SASP Atlas (71), CellAge] to create the master list that includes diverse senescence/SASP and aging genes, which covers broad range of genes related to senescence/SASP, while containing genes of normal pathways such as cell division, cell adhesion indirectly related to senescence/SASP. All genes from 113 BP GO Term categories containing senescence/SASP genes were made into a network and color-coded using Ingenuity Pathway Analysis (IPA) software. These genes were linked to TWIST1 and PDGFB agnostically by adding TWIST1 and PDGFB to the network. Basically, we identified (1) the shortest interaction between TWIST1 and all genes from 113 BP GoTerms, (2) the shortest interaction between PDGFB and all genes from 113 BP GoTerms, and (3) the shortest interaction between the genes from TWIST1 network and PDGFB networks. In the resulting network, all genes that are connected to <4 nodes are removed to reduce the number of genes in the interactome. In a separate analysis, we confirmed the involvement of narrower categories of major senescence/SASP genes (e.g., pro-inflammatory cytokines, proteases and protease inhibitors, and growth factors) using a master list curated from the literature (32); The 128 major senescence/SASP genes were identified in the total number of genes identified by the array (33,297). These senescence/SASP genes were further narrowed down following Benjamini and Hochberg false adjustment and filtered to adjusted *p*-values of <0.05, resulting in a total of 31 significantly differentially expressed senescence/SASP genes, with 16 being significantly downregulated and 15 being significantly upregulated (Supplementary Figure 1C). Heatmaps of these senescence/SASP-related genes were generated in Excel using data from the profile graph generated by GEO2R.

### RNA sequencing and analysis

ECs were isolated from male C57BL6 mouse lungs treated with normoxia or hypoxia for 3 weeks (8 week old, *n* = 3 per group) using anti-CD31 conjugated magnetic beads (41, 66) and isolated ECs were validated by FACS for EC markers (CD31^+^, VE-Cadherin^+^, CD45^−^). RNA was extracted using RNeasy mini kit (QIAGEN). Total RNA samples were submitted to the Institute for Systems Biology Molecular and Cell Core (Seattle, WA) for RNA sequencing. Library preparation was employed using the Illumina TruSeq Stranded mRNA kit. Sequencing was performed using the Illumina NextSeq500. Paired end sequencing was performed on a high output 150 cycle kit v2.5. The RNA sequencing reads were aligned to the mouse genome (mm10 reference genome). Differential gene expression analysis and Fragments Per Kilobase Million (FPKM) calculation were performed with Basepair Tech (www.basepairtech.com) using the DEseq2 pipeline. Significantly differentially expressed genes (335 upregulated and 403 downregulated) were defined as having a log2 fold change >1, and a *p*-adjusted value calculated by the Benjamini-Hochberg adjustment and filtered to <0.05 (Supplementary Table 3). BP GO Term analysis of significant targets was done *via* DAVID v 6.8 using the Functional Annotation Chart tool. Charts were filtered by BP GO Terms and sorted by *p*-value. The 317 BP GO term categories were identified (Supplementary Table 4). Genes related to cellular senescence or the SASP gathered from the GeneCards and CellAge database were agnostically identified in the 178 BP GO Term categories (Supplementary Table 5). The top 50 BP GO Terms were color-coded into groups relating to: transcription/gene expression, protein/RNA processing, cell cycle, inflammatory/immune response, and cell signaling/signal transduction (Supplementary Figure 3A). All genes from top 50 BP GO Term categories containing senescence and SASP genes were made into a network and color-coded using IPA software. The network mapped the shortest interactions among Twist1, Pdgfb, and the genes from top 50 BP GO Term categories related to cellular senescence and SASP. The 133 major senescence and SASP genes curated from literature (32) were identified in the total number of genes identified by the Basepair (19,068). These 133 senescence/SASP genes were further narrowed down to adjusted *p*-values of <0.05 following Benjamini and Hochberg false discovery rate multiple-testing correction of *p*-values, resulting in a total of 14 significantly differentially expressed senescence/SASP genes, with 7 being downregulated and 7 being upregulated. Heatmaps of the 7 upregulated and 7 downregulated senescence/SASP-related genes were generated by Basepair. RNAseq results are available in NCBI Geo (GSE193272).

### Exosome isolation

ECs isolated from tamoxifen-induced *p16*^*iΔEC*^ or *p16*^*fl*/*fl*^ mouse lungs were plated (1 × 10^6^ cells per 6 cm dish), cultured with media containing exosome free FBS, and conditioned media was collected after 24 h. Exosomes were isolated using Total Exosome Isolation Reagent from Cell Culture Media (Thermo Fisher Scientific, Waltham, MA) according to the manufacturer's protocol (41, 72). The exosome pellet was resuspended in 25 μl of 0.2 μm filtered PBS. Isolated exosomes were confirmed with exosome marker proteins (CD63, flotillin-1) using immunoblotting (IB). For transmission electron microscopy (TEM) to analyze the ultrastructure of the exosome, resuspended exosomes were adsorbed onto freshly ionized, 400 mesh formvar/carbon grids, washed once with distilled water, and negatively stained with 2% aqueous Uranyl acetate. Exosome preparations were viewed in a Hitachi H600 transmission electron microscope and images were recorded with a Hamamatsu ccd camera using AMT image capture software. Size and concentration distributions of exosomes were determined using nanoparticle tracking analysis (NTA; NanoSight LM10 system, Malvern instruments, Malvern, UK) (41, 72).

### *In vitro* cell biological assay

HPASMCs (DMEM with 2% serum) were treated with exosomes isolated from tamoxifen-induced *p16*^*iΔEC*^ or *p16*^*fl*/*fl*^ mouse lung ECs with or without hypoxia treatment, and DNA synthesis of HPASMCs was analyzed by an EdU assay (36, 41, 70). HPASMC migration was analyzed using a modified transwell migration assay (41, 70). The cells that migrated toward the 0.5% serum DMEM or supplemented with exosomes (10 μg/ml) through the membrane were stained with Giemsa and counted.

### Proteomics analysis

Proteomics analysis was performed by the Northwestern University Proteomics Core Facility (41) and the Mass Spectrometry Technology Access Center at McDonnell Genome Institute (MTAC@MGI) at Washington University School of Medicine. Isolated exosomes (100 μg) were briefly tip sonicated (~10 s) to break the exosome membrane and purified proteins by acetone/TCA precipitation. Then, the proteins were reduced, alkylated, and digested with trypsin according to the optimized protocol. Digested peptides were desalted on C18 columns then subjected to mass spec analysis. Data was searched against a *Mus musculus* database. Proteomics data analysis on three control (normoxia) and three hypoxia (3 weeks) treated C57BL6 mouse lung EC exosome replicates was performed using Scaffold 5.1.0 software. Total 438 proteins were identified in the control and hypoxia sample replicates (Supplementary Table 6). A cutoff threshold of <8 Total Spectrum Counts in at least one overall replicate was used to further narrow the list of protein of interest, resulting in 37 proteins. Among them, 10 proteins were significantly differentially expressed in the hypoxia- vs. normoxia-treated exosomes. Out of 37 proteins, 30 SASP related proteins were identified using the “SASP Atlas” (50). These 30 SASP related proteins underwent BP GO analysis *via* the Functional Annotation Chart feature of the DAVID v6.8 software and the 51 BP GO term categories were identified (Supplementary Table 7, red bold: significantly differentially expressed proteins). The proteomics data are available *via* ProteomeXchange with identifier PXD033549.

### Statistical analysis

All phenotypic analysis was performed by masked observers unaware of the identity of experimental groups. Error bars (SEM) and *p*-values were determined from the results of three or more independent experiments. Student's *t*-test was used for statistical significance for two groups. For more than two groups, one-way ANOVA with a *post-hoc* analysis using the Bonferroni test was conducted.

## Results

### Senescence increases in IPAH patient PAECs *in vitro*

**Figure 1:**
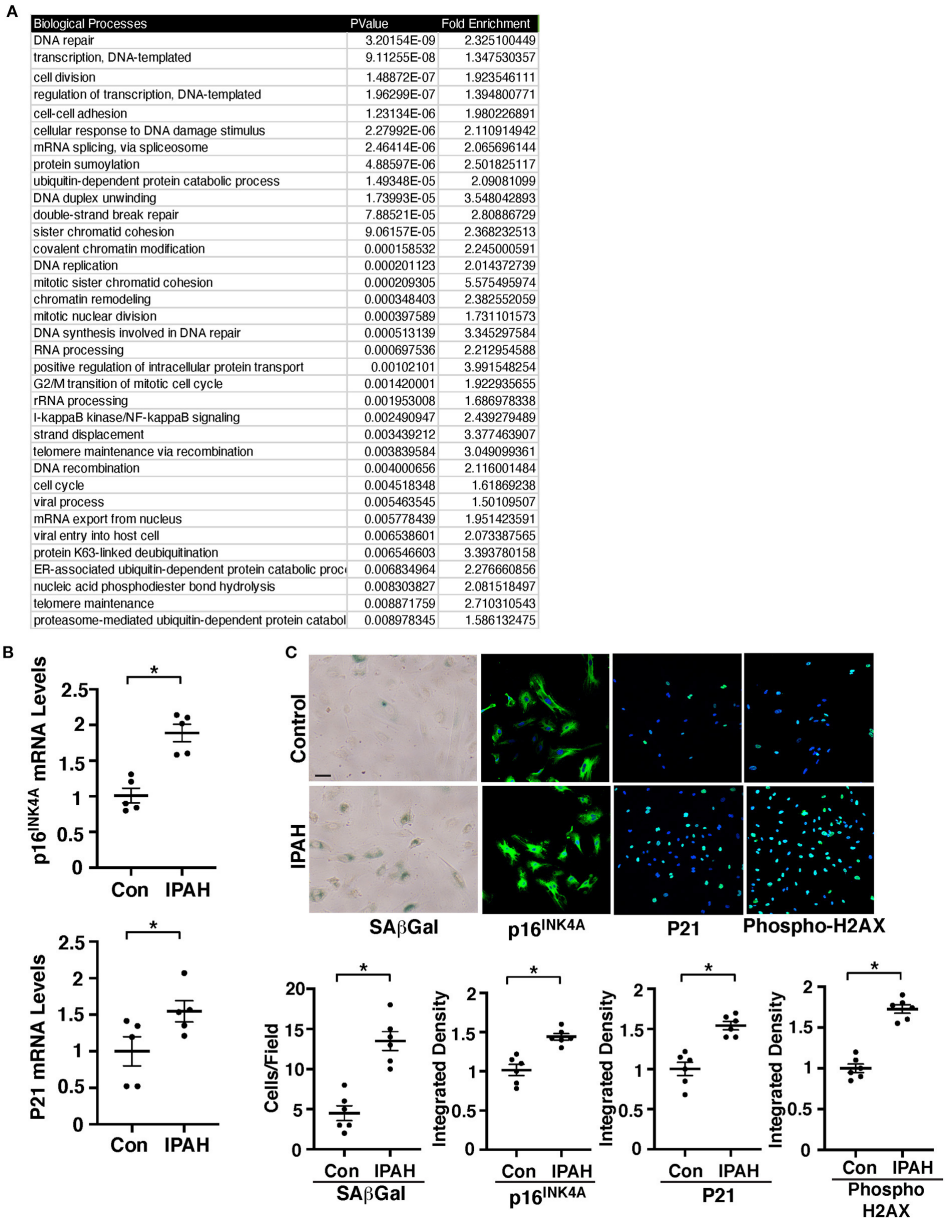
EC senescence increases in IPAH patient lung ECs. **(A)** List of top 35 BP GO term categories derived from significantly differentially expressed genes related to cellular senescence and SASP in control vs. IPAH patient lungs. **(B)** Graphs showing the mRNA levels of p16^INK4A^ (*top*) and p21 (*bottom*) in PAECs from IPAH patients or healthy individuals (*n* = 5, *p < 0.05). Error bars represent SEM. **(C)** Micrographs of PAECs from IPAH patients or healthy individuals showing SAβGal activity (*left*). Immunofluorescence (IF) micrographs of p16^INK4A^ expression and DAPI (*2nd*), p21 expression and DAPI (*3rd*), and the levels of phospho-gamma H2AX and DAPI (*right*). Scale bar, 20 μm. Graphs showing the number of SAβGal-positive cells and integrated fluorescent density of p16^INK4A^, p21, and phospho-gamma H2AX (*n* = 6, *p < 0.05). Error bars represent SEM.

It has been reported that cellular senescence contributes to PH pathology (32, 33), however, the mechanism by which EC senescence controls PH phenotype is not fully understood. Differential expression analysis of publicly available microarray data (GSE113439) comparing 11 control and 6 IPAH patient lungs resulted in 2,075 genes with known gene names at a significance threshold of adjusted *p*-value < 0.001. These genes were sorted into BP GO term categories and 113 BP GO Term categories were found to contain 48 senescence/SASP related genes compiled from the GeneCards database (Supplementary Table 2; Figure 1A; Supplementary Figure 1B). In the separate analysis, 128 major senescence/SASP genes selectively related to pro-inflammatory cytokines, proteases and protease inhibitors, and growth factors curated from literature (32) were identified in the total number of genes (33,297). These senescence/SASP genes were further narrowed down following Benjamini and Hochberg false adjustment and filtered to adjusted *p*-values of <0.05, resulting in 16 downregulated and 15 upregulated genes identified (Supplementary Figure 1C). Gene network analysis of microarray dataset using IPA software reveals that genes from 113 BP GO Term categories containing cellular senescence/SASP genes of normal vs. IPAH patient lungs, which is classified into transcription/gene expression, protein/RNA processing, cell cycle, inflammatory/immune response, cell signaling/signal transduction, or miscellaneous groups, directly or indirectly interacted with TWIST1 (Supplementary Figure 1A). Endothelial TWIST1 mediates hypoxia-induced vascular remodeling by increasing PDGFB expression (11). Although the direct link between TWIST1 and PDGFB was not identified, significantly differentially expressed genes of healthy vs. IPAH patient lungs that interacted with TWIST1 also interacted with PDGFB (Supplementary Figure 1A).

Consistent with microarray data, the mRNA levels of major senescence markers p16^INK4A^ and p21 were 1.8- and 1.5- times higher, respectively, in IPAH patient-derived PAECs compared to those in control healthy PAECs (Figure 1B). Immunocytochemical (ICC) and immunoblotting (IB) data confirmed that the expression of p16^INK4A^ and p21 is upregulated in the PAECs of IPAH patients (Figure 1C; Supplementary Figure 2A). SA β-Gal activity and an early induction of cellular senescence with accumulated DNA damage detected by phospho gamma H2AX staining and IB were also upregulated in IPAH patient-derived PAECs (Figure 1C; Supplementary Figure 2A), suggesting that EC senescence increases in PH.

### Knockdown of endothelial p16^**INK4A**^ attenuates hypoxia-induced vascular remodeling in the mouse lung

**Figure 2:**
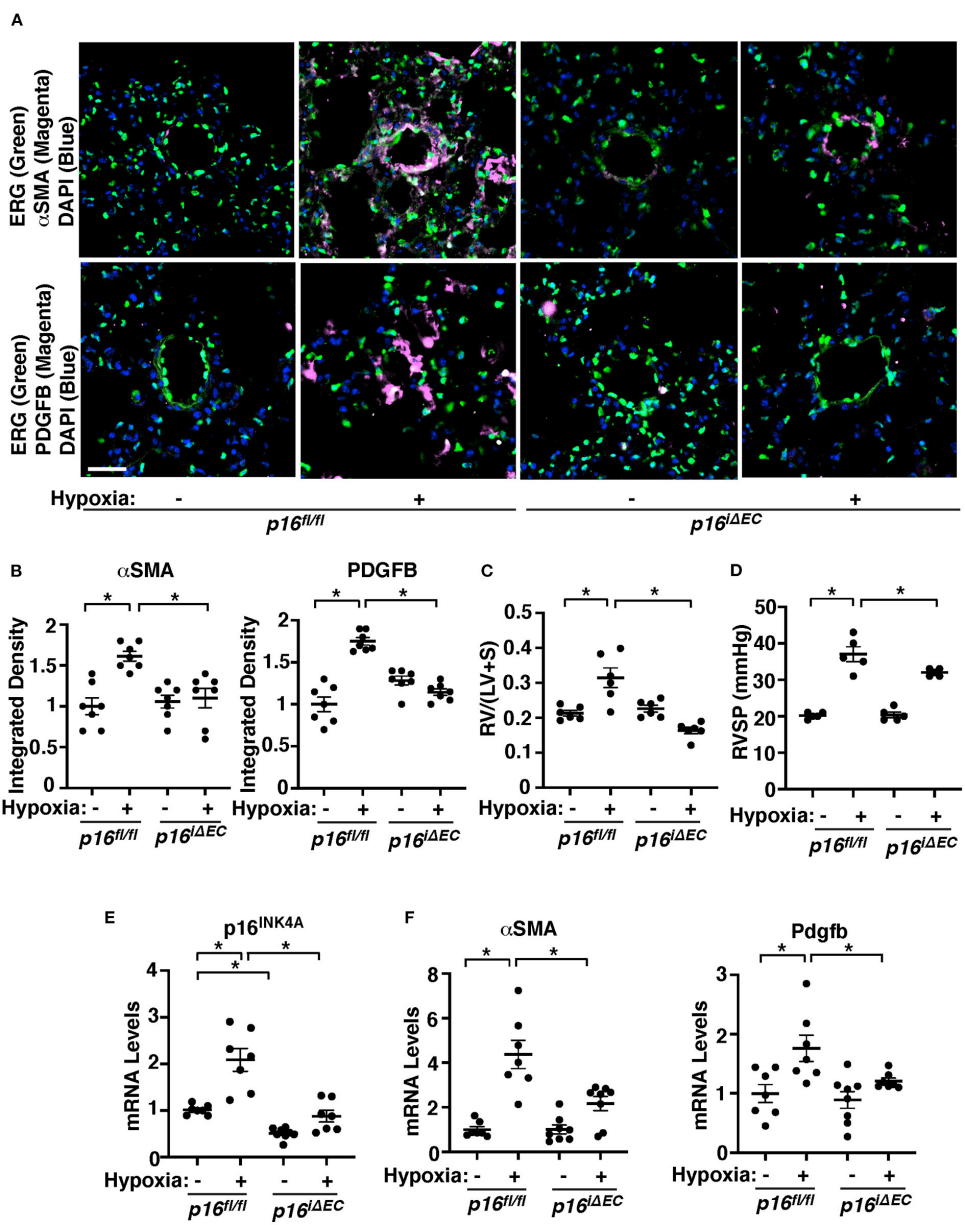
Endothelial p16^INK4A^ mediates hypoxia-induced vascular remodeling in pulmonary arterioles in the mouse lung. **(A)** IF images of representative pulmonary arterioles in the lungs of tamoxifen-induced *p16*^*fl*/*fl*^ or *p16*^*iΔEC*^ mice treated with normoxia or hypoxia for 3 weeks stained for αSMA, ERG, and DAPI (*top*) or PDGFB, ERG, and DAPI (*bottom*). Scale bar: 25 μm. **(B)** Graphs showing integrated fluorescent density of αSMA and PDGFB in tamoxifen-induced *p16*^*fl*/*fl*^ or *p16*^*iΔEC*^ mice treated with normoxia or hypoxia for 3 weeks (*n* = 7, mean ± SEM, *p < 0.05). **(C)** Graph showing Fulton's index (right ventricle/[left ventricle + septum], [RV/(LV + S)]) of tamoxifen-induced *p16*^*fl*/*fl*^ or *p16*^*iΔEC*^ mice treated with normoxia or hypoxia for 3 weeks (*n* = 6, mean ± SEM, *p < 0.05). **(D)** Graph showing right ventricular systolic pressure (RVSP) of tamoxifen-induced *p16*^*fl*/*fl*^ or *p16*^*iΔEC*^ mice treated with normoxia or hypoxia for 3 weeks (*n* = 5, mean ± SEM, *p < 0.05). **(E)** Graph showing the mRNA levels of p16^INK4A^ in the ECs isolated from tamoxifen-induced *p16*^*fl*/*fl*^ or *p16*^*iΔEC*^ mouse lungs treated with normoxia or hypoxia for 3 weeks (*n* = 7–8, mean ± SEM, *p < 0.05). **(F)** Graphs showing the mRNA levels of αSMA and Pdgfb in the tamoxifen-induced *p16*^*fl*/*fl*^ or *p16*^*iΔEC*^ mouse lungs treated with normoxia or hypoxia for 3 weeks (*n* = 7–8, mean ± SEM, *p < 0.05).

Cellular senescence is upregulated in the IPAH patient PAECs (Figure 1). We next examined whether p16^INK4A^ knockdown in ECs attenuates SMC accumulation to the distal PAs (10–100 μm in diameter) in a hypoxia-induced mouse PH model. Consistent with IPAH patient PAECs, when we exposed control *p16*^*fl*/*fl*^ mice (8–10 week old) to hypoxia (8.5% O_2_) for 3 weeks, p16^INK4A^ mRNA levels increased by 2.1-times in mouse lung ECs compared to that treated with normoxia (Figure 2E). Hypoxia-treated *p16*^*fl*/*fl*^ mice exhibited accumulation of αSMA-positive cells to distal PAs (Figures 2A,B), upregulated αSMA mRNA expression (Figure 2F), increased right ventricular hypertrophy evaluated by a Fulton's index (36) (Figure 2C), and raised RVSP (Figure 2D) compared with those treated with normoxia. These effects were attenuated in tamoxifen-induced *p16*^*iΔEC*^ mice (Figures 2A–D,F), in which p16^INK4A^ expression was 52% lower in lung ECs (Figure 2E). To examine the effects of Cre gene, we also treated tamoxifen-induced *Cdh5(PAC)-Cre*^*ERT*2^ control mice with hypoxia and examined the effects on accumulation of αSMA-positive cells to distal PAs, right ventricular hypertrophy, and RVSP. Hypoxia stimulated accumulation of αSMA-positive cells to distal PAs and increased Fulton's index and RVSP in *Cdh5(PAC)-Cre*^*ERT*2^ mice, suggesting that inhibition of αSMA-positive cell accumulation, right ventricular hypertrophy, and RVSP in *p16*^*iΔEC*^ mice is not because of the effects of Cre gene (Supplementary Figures 2B–E). We and others have reported that hypoxia induces vascular remodeling by increasing PDGFB expression (11, 73). Hypoxia-induced increases in PDGFB expression were suppressed in *p16*^*iΔEC*^ mouse lungs when analyzed using IHC (Figures 2A,B) and qRT-PCR (Figure 2F), indicating that EC senescence increases PDGFB expression and mediates the hypoxia-induced pathological accumulation of αSMA–positive cells to distal PAs.

### Endothelial p16^**INK4A**^ mediates hypoxia-induced TWIST1 expression in the mouse lung

**Figure 3:**
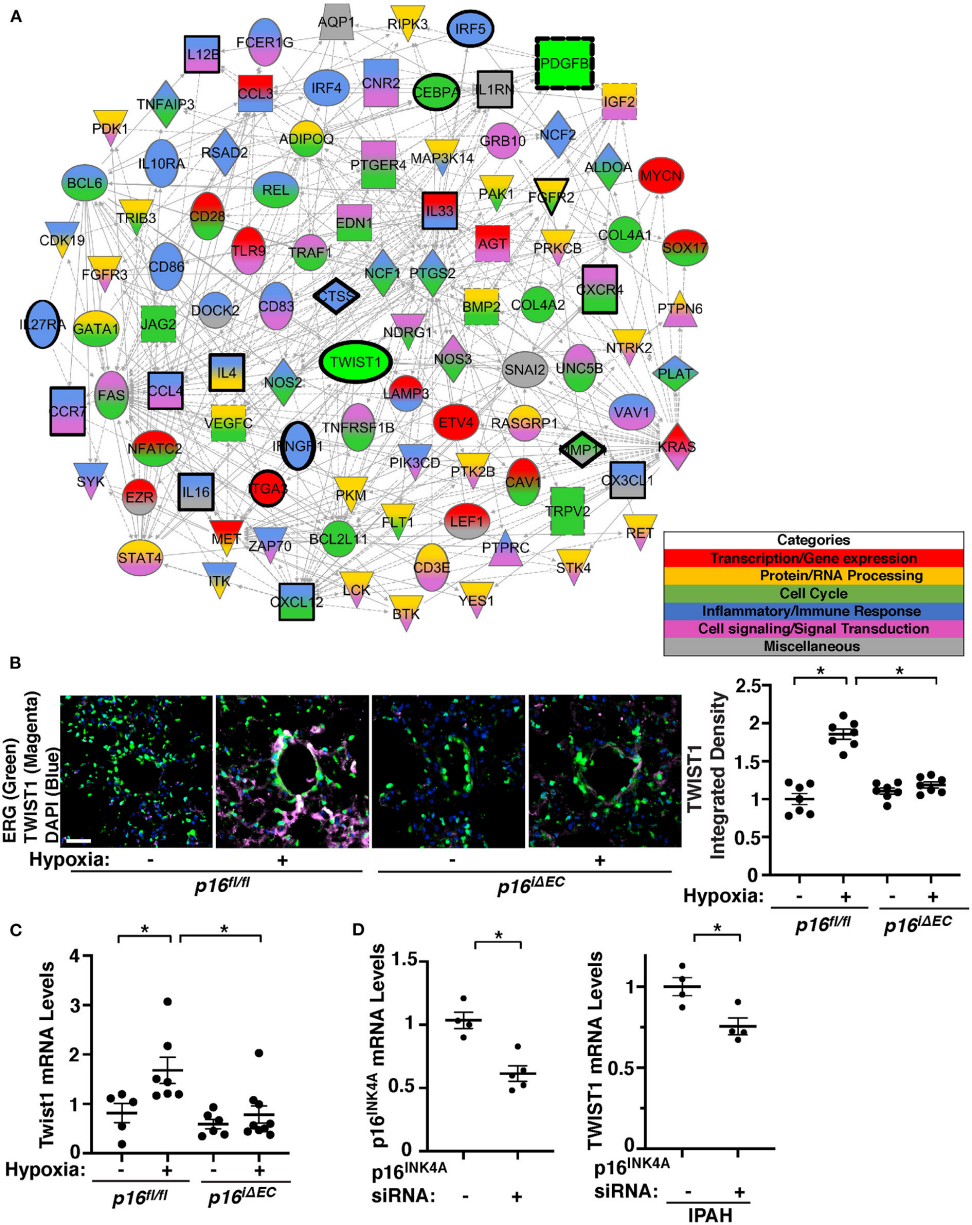
Endothelial p16^INK4A^ mediates hypoxia-induced TWIST1 expression in the mouse lung. **(A)** Gene network demonstrating interactions between Twist1, Pdgfb, and differentially expressed genes listed in the top 50 BP GO Term categories relating to senescence/SASP in ECs isolated from mouse lungs treated with hypoxia for 3 weeks compared to those from normoxia-treated mouse lung ECs. Red: Transcription/gene expression, Gold: Protein/RNA processing, Green: Cell cycle. Blue: Inflammatory/immune response. Pink: Cell signaling/signal transduction, Gray: Miscellaneous. **(B)** IF images of representative pulmonary arterioles in the lungs of tamoxifen-induced *p16*^*fl*/*fl*^ or *p16*^*iΔEC*^ mice treated with normoxia or hypoxia for 3 weeks stained for TWIST1, ERG, and DAPI. Scale bar: 25 μm. Graph showing integrated fluorescent density of TWIST1 in tamoxifen-induced *p16*^*fl*/*fl*^ or *p16*^*iΔEC*^ mouse lungs treated with normoxia or hypoxia for 3 weeks (*n* = 7, mean ± SEM, *p < 0.05). **(C)** Graph showing the mRNA levels of Twist1 in tamoxifen-induced *p16*^*fl*/*fl*^ or *p16*^*iΔEC*^ mouse lungs treated with normoxia or hypoxia for 3 weeks (*n* = 5–9, mean ± SEM, *p < 0.05). **(D)** Graph showing the mRNA levels of p16^INK4A^ in PAECs treated with p16^INK4A^ siRNA or scrambled control siRNA (*left, n* = 4–5, mean ± SEM, *p < 0.05). Graph showing the mRNA levels of TWIST1 in IPAH PAECs treated with p16^INK4A^ siRNA or scrambled control siRNA (*right, n* = 4, mean ± SEM, *p < 0.05).

We have reported that endothelial TWIST1 mediates hypoxia-induced vascular remodeling through PDGFB signaling (11). Network analysis of publicly available microarray data (GSE113439) of control and IPAH patient lungs revealed that the cellular senescence/SASP genes network with one another as well as with TWIST1 or PDGFB (Supplementary Figure 1A). RNAseq analysis of ECs from hypoxia (8.5% O_2_, 3 weeks)—or normoxia-treated mouse lungs also revealed that a total of 19,068 genes were altered. This list was filtered for genes with a >2-fold change and an adjusted *p*-value < 0.01, which narrowed the list to 738 genes (335 upregulated and 403 downregulated) that were significantly differentially expressed (Supplementary Table 3, GSE193272) and 317 BP GO Terms categories were generated (Supplementary Table 4). Of these GO Term categories, 178 categories were identified as categories with senescence/SASP-related genes appeared on a master list comprised of GeneCards and relating to transcription/gene expression, protein/RNA processing, cell cycle, inflammatory/immune response, and cell signaling/signal transduction (Supplementary Table 5; Supplementary Figure 3A). IPA network analysis demonstrated that genes from top 50 BP GO Term categories relating to cellular senescence/SASP interact closely with Twist1 and Pdgfb (Figure 3A). We have reported that (1) TWIST1 overexpression increases the expression of PDGFB in human pulmonary arterial endothelial (HPAE) cells, (2) Twist1 knockdown suppresses hypoxia-induced upregulation of PDGFB expression and accumulation of αSMA–positive cells in the mouse lungs, and (3) IPAH patient-derived PAE cells stimulate accumulation of αSMA–positive cells through endothelial TWIST1-PDGFB signaling (11). Therefore, we next examined whether EC senescence controls TWIST1 expression in the hypoxia-treated mouse lungs. The levels of TWIST1 were 1.8-times higher in the hypoxia-treated *p16*^*fl*/*fl*^ mouse lungs, while the levels were not significantly altered in the tamoxifen-induced *p16*^*iΔEC*^ mouse lungs (Figure 3B). Consistently, the mRNA levels of Twist1 increased in hypoxia-treated *p16*^*fl*/*fl*^ mouse lungs compared to those under normoxia, while the effects were suppressed in *p16*^*iΔEC*^ mouse lungs (Figure 3C). We also confirmed the results using IPAH patient PAECs; p16^INK4A^ knockdown using siRNA transfection decreased the levels of TWIST1 in IPAH PAECs (Figure 3D). These results suggest that EC senescence increases TWIST1 signaling and mediates hypoxia-induced vascular remodeling.

### Inhibition of EC senescence suppresses accumulation of αSMA-positive cells to IPAH ECs in the gel implanted on the mouse lungs

**Figure 4:**
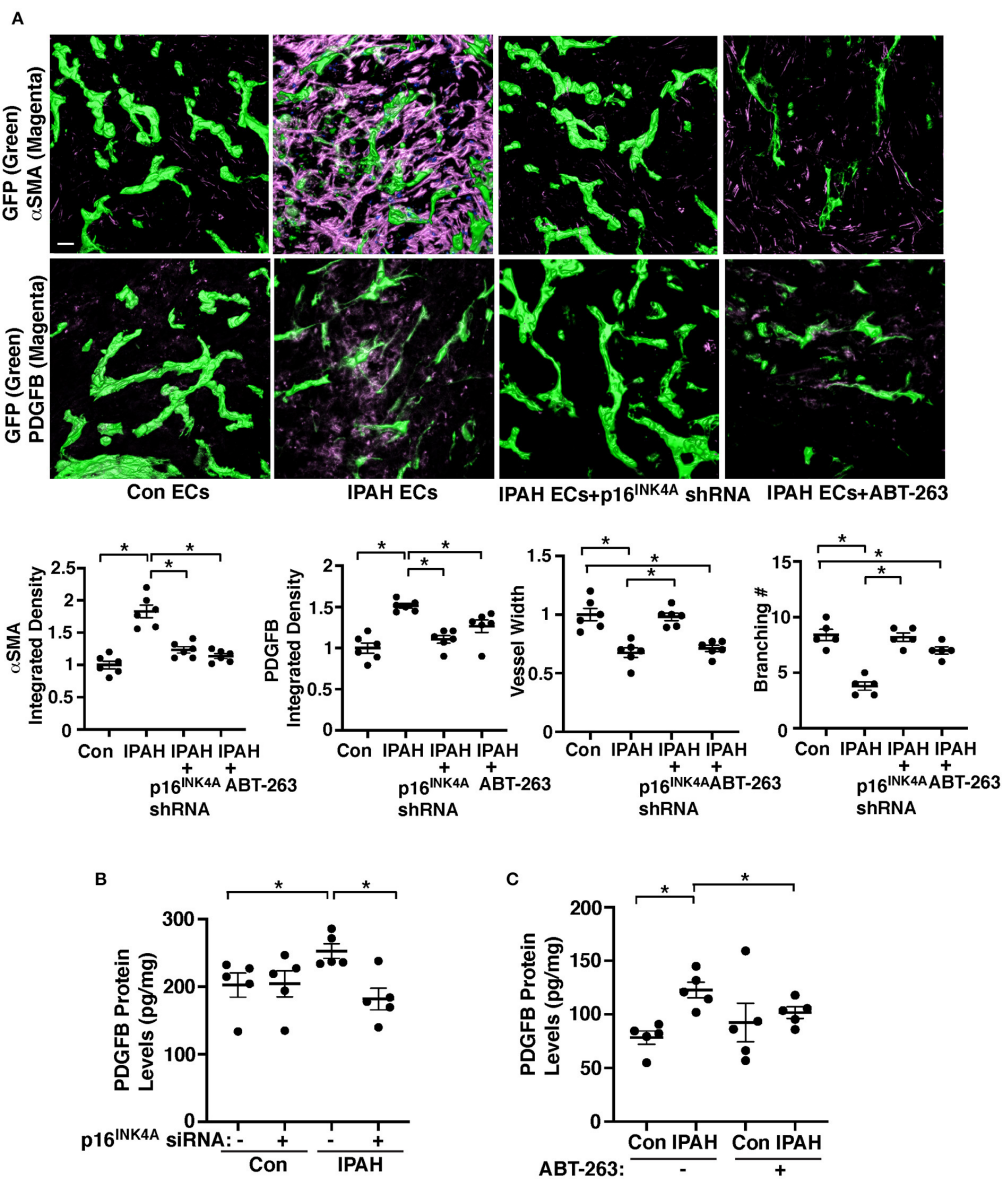
Inhibition of EC senescence suppresses accumulation of αSMA-positive cells and PDGFB expression in the gel implanted on the mouse lung. **(A)** IF micrographs of fibrin gel supplemented with GFP-labeled healthy or IPAH patient ECs or in combination with treatment with p16^INK4^ shRNA or ABT-263 and implanted on the NSG mouse lungs for 7 days; GFP-labeled blood vessels and αSMA expression (*top*) and GFP-labeled blood vessels and PDGFB expression (*bottom*) in the fibrin gel. Scale bar, 50 μm. Graphs showing integrated fluorescent density of αSMA (*left*) and PDGFB (*2nd, n* = 6, mean ± SEM, *p < 0.05), vessel width (*3rd, n* = 6, mean ± SEM, *p < 0.05), and branching number (*right, n* = 5, mean ± SEM, *p < 0.05). **(B)** Graph showing the protein levels of PDGFB measured by ELISA in healthy or IPAH patient PAECs or in combination with treatment with p16^INK4^ siRNA or scrambled control siRNA (*n* = 5, mean ± SEM, *p < 0.05). **(C)** Graph showing the protein levels of PDGFB measured by ELISA in healthy or IPAH patient PAECs or in combination with treatment with ABT-263 (250 nM, *n* = 5, mean ± SEM, *p < 0.05).

To further examine the effects of EC senescence on vascular structures and PASMC accumulation in the mouse lungs, we implanted fibrin gel mixed with IPAH patient ECs or in combination with modulation EC senescence on the lung surface of living mice (35, 36, 41, 66, 67). When we implanted fibrin gel supplemented with PAECs from IPAH patients or healthy individuals on the immunocompromised NOD scid gamma (NSG) mouse lung (8–10 week old) for 7 days (35, 36, 41, 66, 67), GFP-labeled healthy ECs supplemented in the gel formed a well-developed vascular structure in the gel (Figure 4A). Supplementation of IPAH patient-derived PAECs reduced blood vessel formation, increased PDGFB expression, and induced recruitment of αSMA-positive cells from host mouse lungs to accumulate in the gel compared to that in the gel supplemented with healthy ECs (Figure 4A); the levels of αSMA and PDGFB were 1.8- and 1.5-times higher in the gel mixed with IPAH PAECs, while p16^INK4A^ knockdown using lentivirus expressing p16^INK4A^ shRNA or treatment the gel with a senolytic reagent, ABT-263 (1 μg/gel) inhibited accumulation of αSMA-positive cells and PDGFB expression in the gel (Figure 4A).

We also examined the effects of inhibition of EC senescence on PDGFB expression in IPAH patient PAECs. Consistent with the results of hypoxia-treated *p16*^*iΔEC*^ mouse lungs (Figures 2A,B,F), the PDGFB protein levels were significantly higher in IPAH PAECs compared to those in healthy human PAECs when analyzed using ELISA (Figures 4B,C), while the effects were suppressed when p16^INK4A^ was knocked down in IPAH PAECs using siRNA transfection compared to those treated with scrambled control siRNA (Figure 4B) or IPAH PAECs were treated with ABT-263 (250 nM, Figure 4C). These results suggest that EC senescence increases PDGFB expression and mediates αSMA-positive cell accumulation to IPAH PAECs.

### Exosomes from hypoxia-treated mouse lung ECs stimulate SMC recruitment in the fibrin gel implanted on the mouse lungs

**Figure 5:**
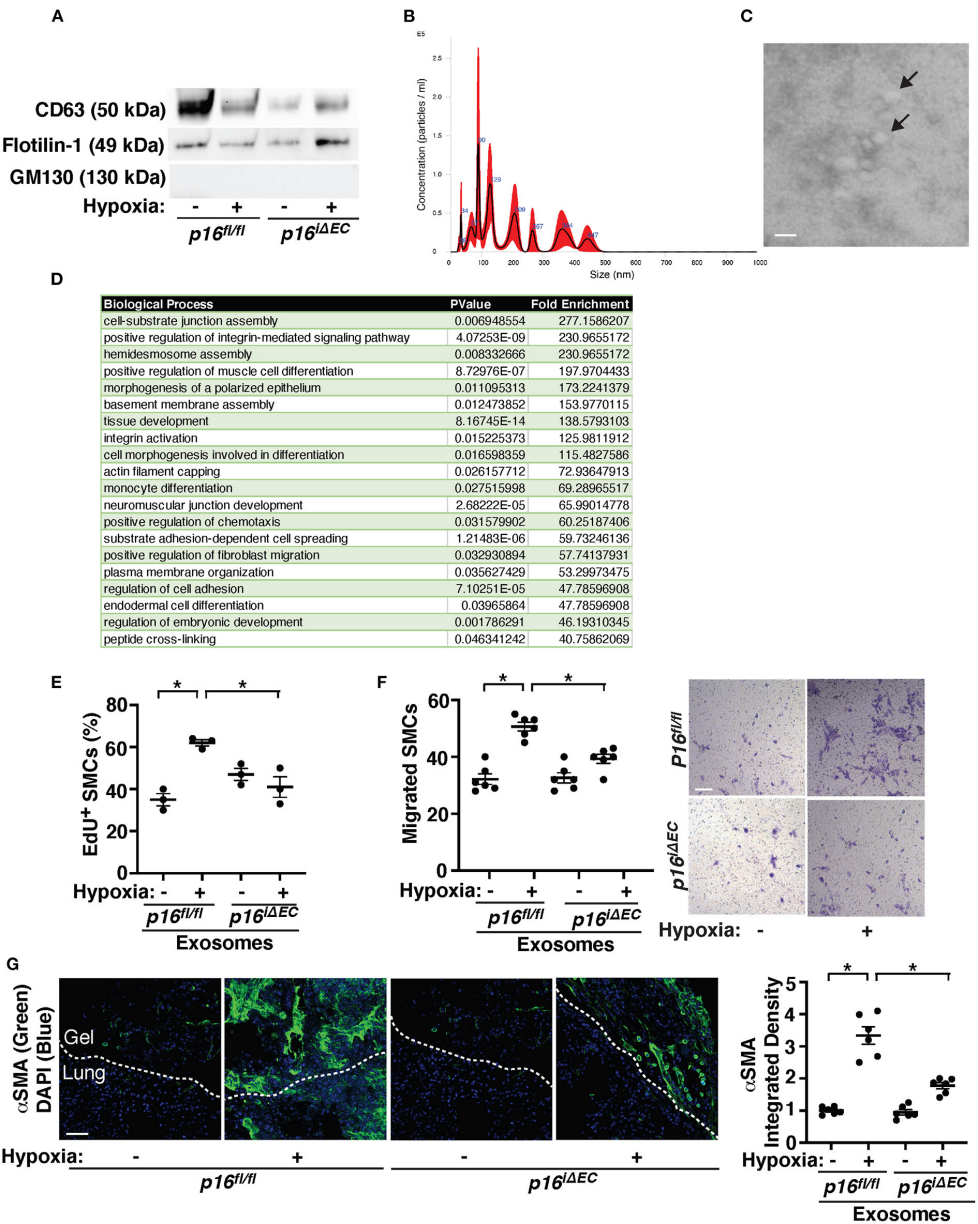
Exosomes from hypoxia-treated mouse lung ECs stimulate recruitment of αSMA-positive cells in the fibrin gel implanted on the mouse lungs. **(A)** IB analysis of CD63, Flotillin-1, and GM130 in exosomes collected from conditioned media of ECs isolated from tamoxifen-induced *p16*^*fl*/*fl*^ or *p16*^*iΔEC*^ mouse lungs treated with normoxia or hypoxia for 3 weeks. **(B)** Size distribution and particle concentration of isolated exosomes analyzed using NTA. **(C)** TEM image of exosome morphology. Scale bar: 150 nm. Arrows indicate exosomes. **(D)** List of top 20 BP GO terms of proteins differentially enriched in exosomes isolated from conditioned media of mouse lung ECs isolated from C57BL6 mouse lungs treated with normoxia or hypoxia for 3 weeks. **(E)** Graph showing EdU-positive PASMCs treated with exosomes (10 μg/ml) collected from conditioned media of ECs isolated from tamoxifen-induced *p16*^*fl*/*fl*^ or *p16*^*iΔEC*^ mouse lungs treated with normoxia or hypoxia for 3 weeks (*n* = 3, mean ± SEM, *p < 0.05). **(F)** Graph showing PASMCs migrating toward medium containing exosomes (10 μg/ml) collected from conditioned media of ECs isolated from tamoxifen-induced *p16*^*fl*/*fl*^ or *p16*^*iΔEC*^ mouse lungs treated with normoxia or hypoxia for 3 weeks (*left, n* = 6, mean ± SEM, *p < 0.05). Representative micrographs showing PASMCs migrating toward medium containing exosomes (10 μg/ml) collected from conditioned media of ECs isolated from tamoxifen-induced *p16*^*fl*/*fl*^ or *p16*^*iΔEC*^ mouse lungs treated with normoxia or hypoxia for 3 weeks (*right*, Wright Giemsa staining). Scale bar, 50 μm. **(G)** IF micrographs of αSMA expression and DAPI in the fibrin gel supplemented with exosomes collected from conditioned media of ECs isolated from tamoxifen-induced *p16*^*fl*/*fl*^ or *p16*^*iΔEC*^ mouse lungs treated with normoxia or hypoxia for 3 weeks and implanted on the *p16*^*fl*/*fl*^ mouse lung for 7 days. Scale bar, 50 μm. Graph showing integrated fluorescent density of αSMA (*n* = 6, mean ± SEM, *p < 0.05).

It has been reported that human MSC-derived exosomes suppress various lung diseases including PH in animal models (53–61). However, the role of EC exosomes in PH pathology and the involvement of EC senescence have not been studied before. When exosomes were isolated from pre-filtered (0.2 μm) conditioned media of ECs (1 × 10^6^ cells) isolated from tamoxifen-induced *p16*^*fl*/*fl*^ or *p16*^*iΔEC*^ mouse lungs treated with hypoxia (41, 72, 74, 75), the isolated exosome population was positive for exosome markers (CD63, Flotillin-1) and negative for the cellular marker GM130 when analyzed using IB (Figure 5A). NTA revealed that isolated EC exosomes were heterogeneous in diameter with 90–130 nm (Figure 5B). TEM images exhibited the typical round vesicular like morphology with ~50–100 nm in size (Figure 5C).

Proteomics analysis of exosomes isolated from conditioned media of three normoxia- vs. three hypoxia-treated mouse lung EC replicates identified 438 proteins present in the control and hypoxia sample replicates (Supplementary Table 6). Proteins with total spectrum count of ≥8 identified 37 proteins, 10 of which were significantly differentially expressed in hypoxia- vs. normoxia-treated exosomes. Out of these 37 proteins, 30 proteins were identified as SASP related proteins based on the “SASP Atlas” (50). These 30 proteins underwent BP GO analysis *via* the Functional Annotation Chart feature of the DAVID v6.8 software and 51 total BP GO term categories were identified (Supplementary Table 7). The top 20 BP GO Term categories derived from the 30 SASP-related proteins were classified as cell differentiation, tissue development, cell signaling/signal transduction, and extracellular matrix (ECM)/cellular junction assembly, which contribute to vascular remodeling and cell-cell communications (Figure 5D). When we treated PASMCs with exosomes collected from hypoxia-treated *p16*^*fl*/*fl*^ mouse lung ECs, PASMC DNA synthesis and migration were induced by 1.7- and 1.6-times, respectively, compared to those treated with normoxia-treated *p16*^*fl*/*fl*^ mouse lung EC-derived exosomes when analyzed using an EdU assay and a transwell migration assay, respectively (Figures 5E,F). However, these effects were suppressed when PASMCs were treated with exosomes derived from hypoxia-treated *p16*^*iΔEC*^ mouse lung ECs (Figures 5E,F).

To confirm the effects of hypoxia-treated mouse lung EC-derived exosomes on PASMC behaviors in the lungs, we implanted fibrin gel supplemented with exosomes (5 μg/gel) isolated from conditioned media of normoxia- or hypoxia-treated *p16*^*fl*/*fl*^ or *p16*^*iΔEC*^ mouse lung ECs. Recruitment of αSMA-positive cells from the host mouse lungs was significantly stimulated in the gel containing hypoxia-treated *p16*^*fl*/*fl*^ mouse lung EC-derived exosomes compared to that supplemented with exosomes from normoxia-treated *p16*^*fl*/*fl*^ mouse lung ECs (Figure 5G). Exosomes isolated from conditioned media of hypoxia-treated *p16*^*iΔEC*^ mouse lung ECs suppressed recruitment of host mouse lung αSMA-positive cells in the gel (Figure 5G), suggesting that senescence-related factors in the hypoxia-treated mouse lung EC-derived exosomes are required for SMC recruitment in the implanted gel.

## Discussion

Here, we have demonstrated that endothelial senescence mediates PH pathology. The levels of senescence markers are higher in IPAH patient PAECs compared to those from healthy individuals. Hypoxia-induced accumulation of αSMA-positive cells to the PAs is attenuated in tamoxifen-induced *p16*^*iΔEC*^ mice. The levels of PDGFB and TWIST1 increase in hypoxia-treated mouse lungs or IPAH patient PAECs, while the effects are attenuated by knocking down p16^*INK*4*A*^ in ECs or treating ECs with senolytic reagent. Exosomes collected from hypoxia-treated mouse lung ECs stimulate SMC DNA synthesis and migration *in vitro* and recruitment of αSMA-positive cells in the gel implanted on the mouse lungs, while exosomes from p16^INK4A^ knocked down ECs inhibit the effects. These results suggest that EC senescence stimulates TWIST1-PDGFB signaling and mediates PH pathology. Modulation of EC senescence could be an effective strategy to manage PH.

Our microarray analysis of control vs. IPAH lungs suggests that among significantly differentially expressed 2075 genes (*p* adj value < 0.001) applied for the BP GO analysis regardless the ranking order, 48 genes were categorized as SASP/senescence-related genes curated from unbiased genomic and proteomic database [SASP Atlas (71), CellAge] (Supplementary Figure 1B). In a separate analysis, we confirmed the involvement of narrower categories of major senescence/SASP genes (e.g., pro-inflammatory cytokines, proteases and protease inhibitors, and growth factors) using a master list curated from the literature (32); 16 downregulated and 15 upregulated significantly differentially expressed senescence/SASP genes were identified (Supplementary Figure 1C). These transcriptome data suggest that senescence/SASP are directly or indirectly involved in the PH pathology.

In the microarray data, while PDGFB was significantly differentially expressed between control vs. IPAH lungs (adjusted *p*-value, 0.0019), TWIST1 was not (adjusted *p*-value, 0.485). We were not able to identify the direct link between TWIST1 and PDGFB in our microarray and RNAseq analyses, however the IPA network indicates that TWIST1 and PDGFB link indirectly through interaction between senescence/SASP genes (Figure 3A; Supplementary Figure 1A). Since TWIST1 phosphorylation is necessary for its nuclear translocation, transcriptional activity, and gene interaction (35, 36), even TWIST1 expression levels were not significantly changed, TWIST1-PDGFB may contribute the PH phenotype. In fact, (1) TWIST1 overexpression increases the expression of PDGFB in HPAE cells, (2) Twist1 knockdown suppresses hypoxia-induced upregulation of PDGFB expression and accumulation of αSMA–positive cells, and (3) IPAH patient-derived PAECs stimulate accumulation of αSMA–positive cells through endothelial TWIST1-PDGFB signaling (11). In addition, (4) PDGFB promoter region has putative TWIST1 binding site, (5) p16^INK4A^ knockdown decreased TWIST1 and PDGFB expression in a hypoxia-induced pulmonary hypertension model and IPAH patient cells (Figures 2–4), (6) significantly differentially expressed genes of healthy vs. IPAH patient lungs that interacted with TWIST1 also interacted with PDGFB (Supplementary Figure 1A). Therefore, although there is no direct link between TWIST1 and PDGFB in the IPA interactome of senescence/SASP related genes, endothelial senescence may control vascular remodeling in PH through TWIST1-PDGFB signaling, and we focused on this signaling in this study. Post-translational modification of TWIST1 or cell type-specific expression of TWIST1 may contribute to the direct interaction between TWIST1 and PDGFB, which is undetectable in the IPA network. Although p16^INK4A^ is a major senescence marker and our results reveal that (1) the levels of p16^INK4A^ are higher in IPAH patient PAECs (Figure 1), (2) knockdown of p16^INK4A^ in ECs prevents hypoxia-induced accumulation of αSMA-positive cells and increases in RVSP (Figure 2), and (3) knockdown of p16^INK4A^ in ECs also decreases TWIST1 and PDGFB expression under hypoxia (Figures 2, 3), p16^INK4A^ was not significantly differentially expressed in ECs isolated from hypoxia-treated mouse lungs in our bulk RNAseq analysis. This may be because of the spatiotemporal changes in the expression of p16^INK4A^ during PH pathology or differential expression of p16^INK4A^ in specific EC subpopulations. Given that there is a spatial and temporal heterogeneity in pulmonary ECs (76), the susceptibility and the level of senescence in response to hypoxia as well as SASP gene profiles (77) may be different among subpopulations of ECs, which directs spatiotemporal differences in vascular remodeling in PH. While the SASP reinforces the senescence through autocrine positive-feedback loop and induces neighbor cells to undergo senescence through paracrine signaling, paracrine signaling of the SASP also promotes proliferation and migration of neighbor cells (27, 77, 78). These functional complexities of senescent ECs drive an endothelial heterogeneity in PH pathology. Further investigation using scRNAseq analysis may elucidate the mechanism.

Our results suggest that EC senescence stimulates TWIST1-PDGFB signaling and mediates PH pathology; knockdown of endothelial p16^*INK*4*A*^ inhibits hypoxia-induced accumulation of PASMCs, RV hypertrophy, and expression of TWIST1 and PDGFB in the mouse lungs (Figures 2, 3). Although hypoxia-induced accumulation of PASMCs to PAs was inhibited in *p16*^*iΔEC*^ mouse lungs, the hypoxia-induced PH phenotype was further accelerated in p16-3MR mice (not shown), in which other types of p16^INK4A+^ senescent cells are also eliminated upon treatment with ganciclovir (GCV) (79); while inhibition of EC senescence in *p16*^*iΔEC*^ mice suppresses PASMC accumulation to PAs, PASMC senescence may also be inhibited in p16-3MR mice, which stimulates proliferation and migration of PASMCs to accumulate to PAs. It is important to note that treatment the gel with the senolytics ABT-263 suppressed both vascular network formation and PASMC accumulation to PAs in the gel (Figure 4A). This inconsistency may be because of the differences in the experimental condition (e.g., senolytics vs. gene knockdown, treatment timeline, and sensitivity of the senolytics to different cell types). The senolytic effects of ABT-263 on other recruited cells, which inhibits SASP factor secretion to inhibit not only SMC accumulation but also EC angiogenic activity to suppress vascular network formation in the gel. In addition to SMCs, EC senescence and subsequent stimulation of secretion of PDGFB and SASP factors may also alter behaviors of other αSMA-positive cells (e.g., pericytes and myofibroblasts) and other lung cells (e.g., epithelial cells and immune cells) (80) to indirectly change vascular structures and αSMA-positive cell accumulation in the hypoxia-treated mouse lungs. Furthermore, in *p16*^*iΔEC*^ mice p16^INK4A^ expression is knocked down in ECs not only in the lungs but also in other organs. Suppression of EC senescence in other organs (e.g., cardiac ECs) and associated changes in the systemic metabolism may affect hypoxia-induced PH phenotype in *p16*^*iΔEC*^ mice. Knockdown of p16^INK4A^ in these other cell types or other organs, or PA-specific knockdown of p16^INK4A^ will further elucidate the mechanism.

We have demonstrated that hypoxia-induced PH phenotype was attenuated in *p16*^*iΔEC*^ mice (Figures 2, 3) and knock down of p16^INK4A^ inhibits increases in the levels of TWIST1 in hypoxia-treated mouse lungs and IPAH patient PAECs (Figure 3). Senescence-related signaling molecules being up- or down-regulated in IPAH patient lungs and hypoxia-treated mouse lung ECs interact with TWIST1 (Figure 3; Supplementary Figure 1), which may directly or indirectly control TWIST1 expression and activity during PH progression. For example, HIF1α expression is significantly increased in IPAH patient lungs and interacts with TWIST1 (Supplementary Figure 1). Since the promoter region of TWIST1 has a HIF1α binding site [−651, −82, CACGT (81)], HIF1α may control TWIST1 transcription in PH ECs. Our IPA network analysis also reveals that HIF1α and other senescence-related genes differentially expressing in IPAH patient lungs interact with PDGFB (Supplementary Figure 1A). Since the promoter region of PDGFB also has HIF1α binding sites [−62, −486, −489, CACGT (81)] and HIF1α is known to control PDGFB expression in breast cancer cells (82), HIF1α may also directly control PDGFB expression in PAECs in PH. Recently it is reported that HIF1 mediates hypoxia-induced endothelial deficiency of iron-sulfur (Fe-S) biogenesis to induce endothelial senescence and PH phenotype (33). Thus, differential and reciprocal regulation of HIF1-3α genes is necessary for PH pathology (83). Although HIF1α controls multiple hypoxia-induced signaling pathways (81) to mediate PH pathology, HIF1α expression was not significantly differentially expressed in our RNAseq data from hypoxia-treated mouse lung ECs (3 weeks). Also while HIF3α was significantly increased in hypoxia-treated mouse lung ECs (Supplementary Figure 3B), HIF3α expression was significantly lower in IPAH patient lungs (Supplementary Figure 1C). In fact, the BP GO term categories in the microarray data of IPAH patient lungs and RNAseq data of hypoxia-treated mouse lung ECs are not identical; BP GO terms of transcription and cell cycle genes are altered more in the IPAH patient lungs, while inflammatory gene categories are changed more in the hypoxia-treated mouse lung ECs. This may be because of the differences in the sample conditions (whole lung samples vs. ECs, human vs. mouse, sample demographics, time course, contribution of factors other than hypoxia). Further investigation of the gene expression patterns and sample profiles will elucidate the mechanism.

In addition to PDGFB (11), TWIST1 is a bHLH transcription factor and controls expression of other angiogenic genes that contain an E-box in their promoter region [e.g., TGFβ2 (84), VEGFR2 (85), Tie2 (63), TGFβR2 (36)] (86), which mediates PH phenotype in a cooperative way. It is known that TWIST1 is also involved in endothelial to mesenchymal transition (EndMT) (36, 87–89). We have reported that hypoxia-treated HPAECs exhibit EndMT, which is inhibited by TWIST1 knockdown that attenuates the accumulation of PASMCs to distal PA in a mouse hypoxia-induced PH model (36). It has been demonstrated that senescent ECs show EndMT phenotype (90). Consistently, IPAH patient-derived PAECs exhibited EndMT phenotype; SLUG expression increased, while VE-cadherin-positive cell–cell junctional structures were disrupted in IPAH patient-derived PAECs compared with control healthy human PAECs when analyzed by ICC (Supplementary Figure 2F). These effects were attenuated when p16^INK4A^ expression was knocked down using siRNA transfection (Supplementary Figure 2F). Thus, cellular senescence may contribute to PH pathology through EndMT signaling as well. Other pathways known to mediate the PH pathology [e.g., eNOS (91), High Mobility Group AT-hook 1 (HMGA1) (92), SMAD (36, 93), PGC1α/TFAM (94)] may also be involved in the mechanism. For example, mitochondrial dysfunction that stimulates cellular senescence during aging (95, 96) contributes to PH pathology (94). TWIST1 controls expression of PGC1α that stimulates mitochondrial biogenesis (97–99) and angiogenesis (98, 100, 101) to mediate age-dependent inhibition of angiogenesis and lung regeneration (37). Inhibition of TWIST1 activity also increases the expression of PGC1α in fat cells (102). PGC1α controls age-dependent mitochondrial metabolism (97) and mediates aging-related cardiovascular diseases (98, 103–105). Thus, although our results suggest that cellular senescence induces TWIST1 expression, there may be a feedback mechanism; TWIST1 may control cellular senescence as reported in mesenchymal stem cells (106) and tumor cells (107) and contribute to PH phenotype through mitochondrial signaling.

We have demonstrated that exosomes isolated from hypoxia-treated *p16*^*fl*/*fl*^ mouse lung ECs induce SMC accumulation to ECs in the gel implanted on the mouse lungs, while these effects are inhibited when treated with exosomes from *p16*^*iΔEC*^ mouse lung ECs (Figure 5). This is consistent with the data demonstrating that senolytics prevent vascular remodeling of IPAH patient ECs (Figure 4). Although senolytics are known to attenuate tissue injury, extend lifespan and delay age-related conditions (28), given the beneficial effects of senescent cells on tissue regeneration/repair (18, 28), senolytics may have harmful side effects. In fact, although senolytic ABT-263 inhibits SMC accumulation, vascular network formation was also suppressed in the gel (Figure 4A). It is known that senescent cells can mediate paracrine effects on adjacent cells (45, 51, 52) through release of exosomes that contain SASP factors and other proteins/nucleic acids regulating cellular senescence (45, 50–52, 77, 108–110). Since exosomes contain multiple proteins and nucleic acids (e.g., ECM molecules, cytoskeleton remodeling molecules), they may reduce the adverse effects of senolytics alone. Furthermore, exosomes are small in size and protected from degradation due to their lipid bilayer structure, which facilitates delivery to their target with a low immune response (39, 40, 42, 44, 45, 111, 112). In fact, MSC-derived exosomes suppress PH and various lung diseases associated with PH (e.g., BPD, airway inflammation, and pulmonary fibrosis) (53–61). Understanding the effects of EC exosomes on PH pathology, combination of senolytic reagents with EC exosomes, and appropriate therapeutic timing will lead to the development of promising strategies for the management of PH. In our results, CD63 levels were lower in exosomes isolated from hypoxia-treated mouse lung ECs or *p16*^*iΔEC*^ mouse lung ECs (Figure 5A). This is consistent with others' reports demonstrating that the expression of CD63 is suppressed in exosomes isolated from hypoxia treated cells (113), while upregulated in the plasma exosomes collected from aged mice, in which senescence is upregulated (114). Given that CD63^+^ exosomes are key effectors in old exosomes (114), investigation of the role of exosome subfraction in PH pathology would further elucidate the mechanism.

We have been using a unique method to implant fibrin gel supplemented with fluorescently labeled control vs. IPAH ECs, in which gene expression is manipulated, or to implant the gel containing senolytic agent on the lung surface of a living mouse (Figure 4A) (35, 36, 41, 66, 67, 115). This method is important and significant to study vascular structures and function in the lung microenvironment, which is significantly different from the systemic vascular system (e.g., negative pleural pressure and low PA pressure) (116, 117). This method also enables us to clearly visualize the lung specific vascular structures and precisely analyze the process and mechanisms of blood vessel formation and interactions between ECs and other resident lung cells recruited from the host lung (e.g., SMCs, alveolar epithelial cells, immune cells, and fibroblasts) in the gel (35, 36, 41, 66, 67, 115), which cannot be done using the subcutaneous gel implantation. In fact, (1) recruited blood vessels are derived from PA (35); when PA is ligated, the blood vessel recruitment into the gel is significantly attenuated, and (2) the morphology of blood vessels is significantly different in the gel implanted on the lung compared to those in the gel implanted under the skin (66). The recruited host lung cells [e.g., alveolar capillaries, epithelial cells, and macrophages (35)] secrete angiogenic/growth factors, and may create lung-specific microenvironment in the gel. Further investigation using this system will enable elucidating the paracrine signaling mechanism by which senescent ECs control behaviors of SMCs in the gel in the lung-specific microenvironment by manipulating gene expression in ECs.

We have investigated the role of EC senescence in αSMA-positive cell accumulation using ECs isolated from IPAH patient PAs, the region >5 mm in diameter. We excluded the samples from >55 years old patients, which are more susceptible to cellular senescence and senescence-related lung diseases such as COPD or IPF that affect PH phenotype in different ways. However, the heterogeneity of the samples due to cardiopulmonary condition (e.g., chronic lung diseases, inflammation), obesity, sex, race, and type-2 bone morphogenetic protein receptor (BMPR2) mutations, which contribute to severity of PH phenotype (3, 118), may impact EC senescence and vascular remodeling. DNA damage inhibits BMPR2 expression and reduced BMPR2 signaling impairs DNA damage repair processes (32, 119), suggesting the involvement of reciprocal interaction of cellular senescence and BMPR2 mutant in severity of PH. While we investigated the effects of EC senescence on SMC accumulation in this study, it is also reported that EC senescence drives the transition from a reversible to irreversible pulmonary vascular phenotype at end-stage of PH progression (32). Obesity is also associated with PH (120) and cellular senescence is induced in an obese condition (121), in which angiogenesis is impaired through TWIST1 signaling (70). Further investigation in another cohort with a larger sample size including the patients with BMPR2 mutation and/or different stages will elucidate the mechanism of EC senescence in the PH pathology.

In summary, we have demonstrated that endothelial senescence increases TWIST1 and PDGFB expression and mediates PH pathology. Knockdown of p16^INK4A^ in ECs attenuates the levels of PDGFB and TWIST1 in IPAH patient PAECs or hypoxia-treated mouse lungs and suppresses accumulation of αSMA–positive cells to PAs in the mouse lungs. These findings suggest that modulation of endothelial senescence will lead to the development of better strategy for the management of PH.

## Data availability statement

The datasets presented in this study can be found in online repositories. The names of the repository/repositories and accession number(s) can be found in the article/Supplementary material.

## Ethics statement

The animal study was reviewed and approved by Animal Care and Use Committee of Medical College of Wisconsin.

## Author contributions

Conceived and designed the experiments: TM and AM. Performed the experiments: PK, KH, TH, KM, TM, and AM. Analyzed the data and contributed reagents/materials/analysis tools: PK, KH, TH, TM, and AM. Wrote the paper: PK, TM, and AM. All authors contributed to the article and approved the submitted version.

## Funding

This work was supported by funds fromNIHR21AG054830, R01HL139638, R21AG062893, and NIH R01HL142578 (to AM and TM), and American Heart Association (AHA) 18TPA34170129 (to AM) as well as 967800 (to AM). Proteomics analysis was performed by the Northwestern Proteomics Core Facility supported by NCI CCSG P30 CA060553 awarded to the Robert H. Lurie Comprehensive Cancer Center, instrumentation award (S10OD025194) from NIH Office of Director, and the National Resource for Translational and Developmental Proteomics supported by P41 GM108569 as well as the Mass Spectrometry Technology Access Center at McDonnell Genome Institute (MTAC@MGI) at Washington University School of Medicine.

## Conflict of interest

The authors declare that the research was conducted in the absence of any commercial or financial relationships that could be construed as a potential conflict of interest.

## Publisher's note

All claims expressed in this article are solely those of the authors and do not necessarily represent those of their affiliated organizations, or those of the publisher, the editors and the reviewers. Any product that may be evaluated in this article, or claim that may be made by its manufacturer, is not guaranteed or endorsed by the publisher.

## Supplementary material

Supplementary Figure 1 Gene networks of TWIST1, PDGFB and senescence-related genes differentially expressed in IPAH patient lungs. **(A)** Network of genes from 113 GO Term categories containing cellular senescence/SASP genes and their relationship to TWIST1 and PDGFB. Red: Transcription/Gene Expression, Gold: Protein/RNA processing, Green: Cell cycle, Blue: Inflammatory/Immune Response, Pink: Cell Signaling/Signal Transduction, Gray: Miscellaneous. **(B)** Heatmap of the 37 upregulated and 11 downregulated cellular senescence/SASP related genes in the 113 BP GO Term categories in control vs. IPAH patient lungs. **(C)** Heatmap of the 15 upregulated and 16 downregulated major cellular senescence/SASP genes in control vs. IPAH patient lungs.

Supplementary Figure 2 Cellular senescence mediates EndMT in IPAH patient derived ECs. **(A)** Representative IB showing the expression of p16^INK4^, p21, phospho gamma H2AX, and β-actin in PAECs from IPAH patients or healthy individuals. Graph showing the protein levels of p16^INK4^, p21, and phospho gamma H2AX in PAECs from IPAH patients or healthy individuals (*n* = 3, mean ± SEM, ^*^*p* < 0.05). **(B)** IF images of representative pulmonary arterioles in the lungs of tamoxifen-induced *Cdh5(PAC)-Cre*^*ERT*2^ mice treated with normoxia or hypoxia for 3 weeks stained for αSMA, ERG, and DAPI (*top*) or PDGFB, ERG, and DAPI (*bottom*). Scale bar: 25 μm. **(C)** Graphs showing integrated fluorescent density of αSMA and PDGFB in tamoxifen-induced *Cdh5(PAC)-Cre*^*ERT*2^ mouse lungs treated with normoxia or hypoxia for 3 weeks (*n* = 6, mean ± SEM, ^*^*p* < 0.05). **(D)** Graph showing Fulton's index (right ventricle/[left ventricle + septum], [RV/(LV + S)]) of tamoxifen-induced *Cdh5(PAC)-Cre*^*ERT*2^ mice treated with normoxia or hypoxia for 3 weeks (*n* = 5–6, mean ± SEM, ^*^*p* < 0.05). **(E)** Graph showing right ventricular systolic pressure (RVSP) of tamoxifen-induced *Cdh5(PAC)-Cre*^*ERT*2^ mice treated with normoxia or hypoxia for 3 weeks (*n* = 5, mean ± SEM, ^*^*p* < 0.05). **(F)** IF micrographs of VE-cadherin and SLUG expression and DAPI in healthy or IPAH patient PAECs or in combination with treatment with p16^INK4^ siRNA or scrambled control siRNA. Scale bar, 20 μm.

Supplementary Figure 3 BP GO term categories of senescence-related genes differentially expressed in hypoxia-treated mouse lung ECs. **(A)** Top 50 BP GO Term categories of senescence-related genes derived from significantly differentially expressing genes in control vs. hypoxia-treated mouse lung ECs. The color-coding corresponds to the network color key. Red: Transcription/Gene Expression, Gold: Protein/RNA processing, Green: Cell cycle, Blue: Inflammatory/Immune Response, Pink: Cell Signaling/Signal Transduction, Gray: Miscellaneous. **(B)** Heatmap of the 7 upregulated and 7 downregulated cellular senescence/SASP genes in normoxia- vs. hypoxia-treated mouse lung ECs.
