# Supplementary figures and images for: Endothelial senescence mediates hypoxia-induced vascular remodeling by modulating PDGFB expression
Source: Front Med (Lausanne). 2022 Sep 20;9:908639. doi: 10.3389/fmed.2022.908639 (PMC9530050; doi:10.3389/fmed.2022.908639)

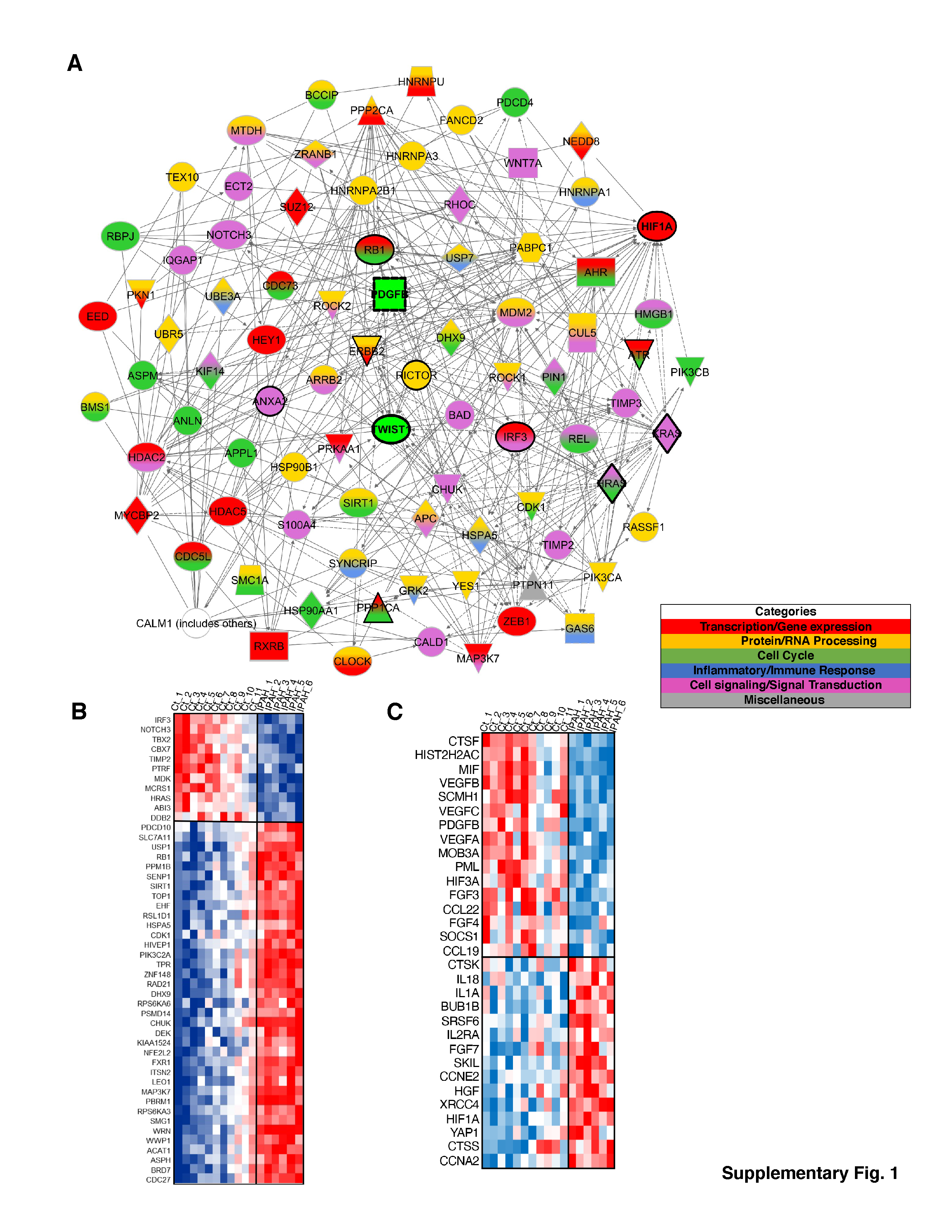

Supplement: Supplementary Figure 1 — Gene networks of TWIST1, PDGFB and senescence-related genes differentially expressed in IPAH patient lungs. (A) Network of genes from 113 GO Term categories containing cellular senescence/SASP genes and their relationship to TWIST1 and PDGFB. Red: Transcription/Gene Expression, Gold: Protein/RNA processing, Green: Cell cycle, Blue: Inflammatory/Immune Response, Pink: Cell Signaling/Signal Transduction, Gray: Miscellaneous. (B) Heatmap of the 37 upregulated and 11 downregulated cellular senescence/SASP related genes in the 113 BP GO Term categories in control vs. IPAH patient lungs. (C) Heatmap of the 15 upregulated and 16 downregulated major cellular senescence/SASP genes in control vs. IPAH patient lungs. [file Image_1.TIFF]

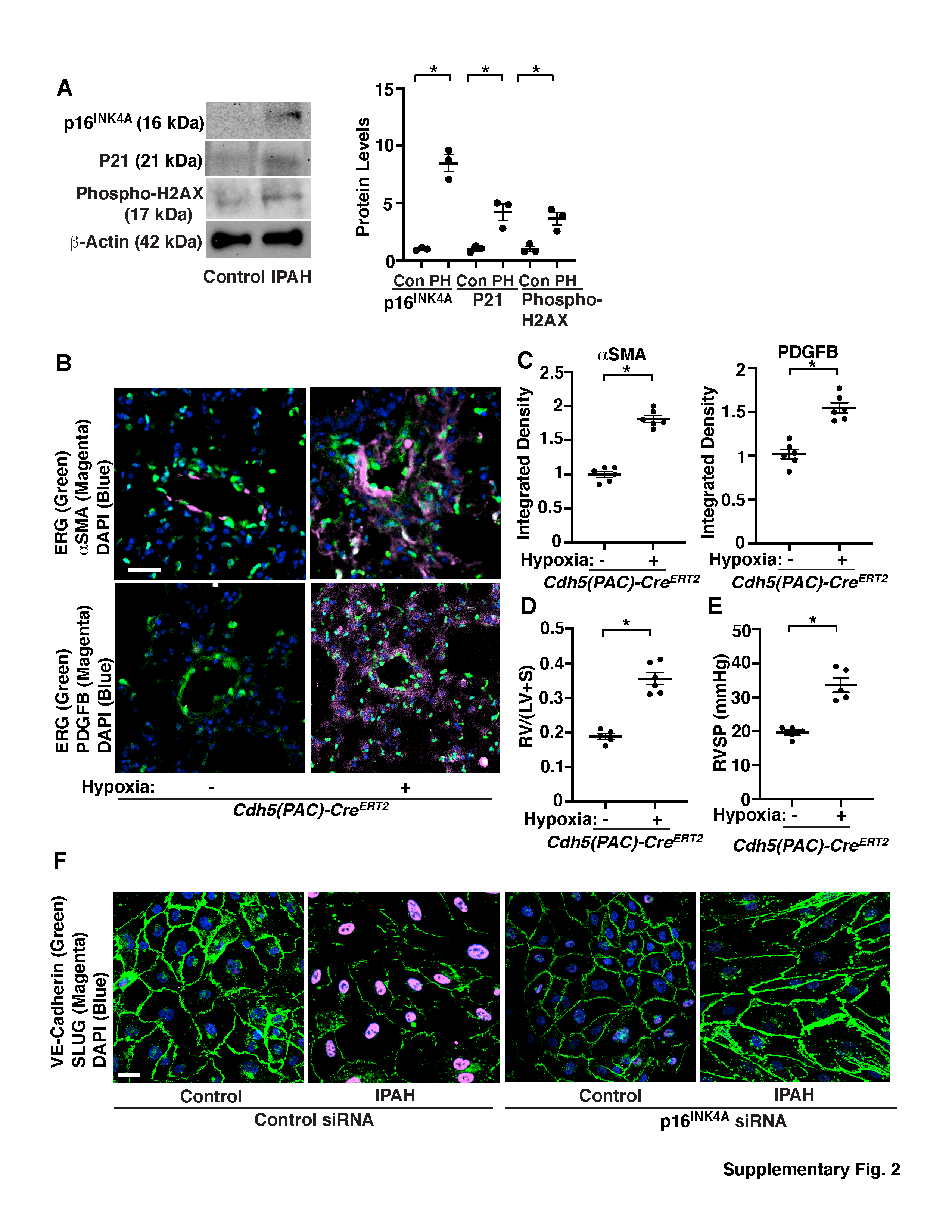

Supplement: Supplementary Figure 2 — Cellular senescence mediates EndMT in IPAH patient derived ECs. (A) Representative IB showing the expression of p16INK4, p21, phospho gamma H2AX, and β-actin in PAECs from IPAH patients or healthy individuals. Graph showing the protein levels of p16INK4, p21, and phospho gamma H2AX in PAECs from IPAH patients or healthy individuals (n = 3, mean ± SEM, *p < 0.05). (B) IF images of representative pulmonary arterioles in the lungs of tamoxifen-induced Cdh5(PAC)-CreERT2 mice treated with normoxia or hypoxia for 3 weeks stained for αSMA, ERG, and DAPI (top) or PDGFB, ERG, and DAPI (bottom). Scale bar: 25 μm. (C) Graphs showing integrated fluorescent density of αSMA and PDGFB in tamoxifen-induced Cdh5(PAC)-CreERT2 mouse lungs treated with normoxia or hypoxia for 3 weeks (n = 6, mean ± SEM, *p < 0.05). (D) Graph showing Fulton's index (right ventricle/[left ventricle + septum], [RV/(LV + S)]) of tamoxifen-induced Cdh5(PAC)-CreERT2 mice treated with normoxia or hypoxia for 3 weeks (n = 5–6, mean ± SEM, *p < 0.05). (E) Graph showing right ventricular systolic pressure (RVSP) of tamoxifen-induced Cdh5(PAC)-CreERT2 mice treated with normoxia or hypoxia for 3 weeks (n = 5, mean ± SEM, *p < 0.05). (F) IF micrographs of VE-cadherin and SLUG expression and DAPI in healthy or IPAH patient PAECs or in combination with treatment with p16INK4 siRNA or scrambled control siRNA. Scale bar, 20 μm. [file Image_2.TIFF]

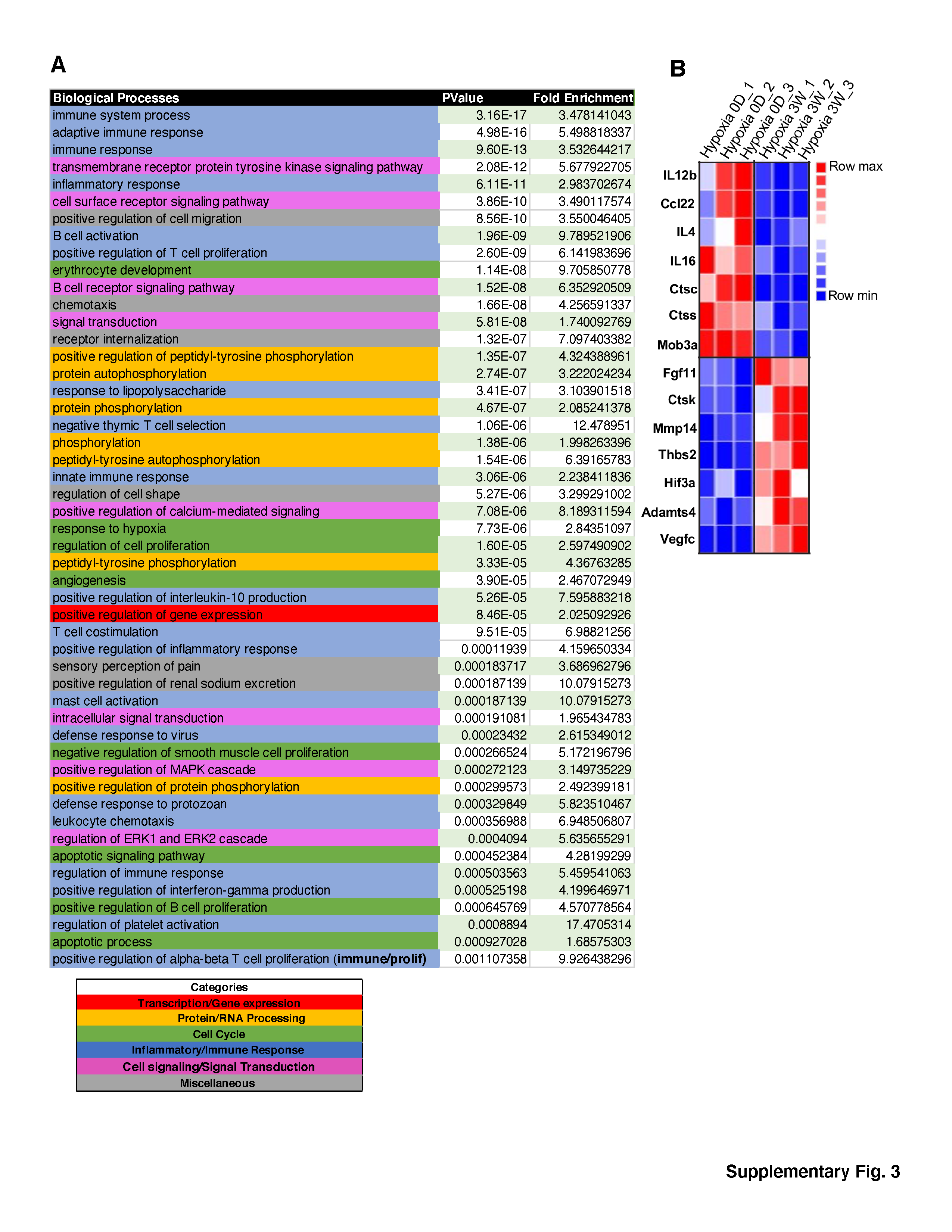

Supplement: Supplementary Figure 3 — BP GO term categories of senescence-related genes differentially expressed in hypoxia-treated mouse lung ECs. (A) Top 50 BP GO Term categories of senescence-related genes derived from significantly differentially expressing genes in control vs. hypoxia-treated mouse lung ECs. The color-coding corresponds to the network color key. Red: Transcription/Gene Expression, Gold: Protein/RNA processing, Green: Cell cycle, Blue: Inflammatory/Immune Response, Pink: Cell Signaling/Signal Transduction, Gray: Miscellaneous. (B) Heatmap of the 7 upregulated and 7 downregulated cellular senescence/SASP genes in normoxia- vs. hypoxia-treated mouse lung ECs. [file Image_3.TIFF]
