# Supplementary material for: Endothelial senescence mediates hypoxia-induced vascular remodeling by modulating PDGFB expression
Source: Front Med (Lausanne). 2022 Sep 20;9:908639. doi: 10.3389/fmed.2022.908639 (PMC9530050; doi:10.3389/fmed.2022.908639)
Supplement: Supplementary file 4 [file Data_Sheet_1.PDF]

Biological Processes	PValue	Fold Enrichment	Genes
DNA repair	3.15E-10	2.50640469	SMG1, BCCIP, MUM1, SMC3, ALKBH2, EPC2, TOPBP1, NBN, POLK, GEN1, PARP3, SUPT16H, USP3, USP45, ATRX, RECQL, SMC1A, SIRT1, UCHL5, DDB2, KIN, RRM2B, FANCD2, MORF4L2, PSME4, OTUB1, BLM, INO80E, BOD1L1, XPA, ZBTB1, PDS5B, BRCC3, PDS5A, TFPT, WRN, UBR5, RBBP8, FANCI, MBD4, POLM, ERCC6L2, MCRS1, APEX2, CDC5L, GTF2H1, RAD50, TAOK3, TAOK1, JMY, CDK1, SPRTN, TRIP12, RAD18, ATR
cell division	2.16E-08	2.050043732	RB1, USP37, SYCP2, CLTC, KIF14, CIB1, SMC5, KIF11, SMC3, SMC4, CDC73, CKS1B, SMC2, PPP1CB, MIS18BP1, CNTRL, CDC27, KNTC1, FBXO5, SPDL1, LZTS2, LRRCC1, NSMCE2, LIG4, PRPF40A, KNL1, SMC1A, TERF1, CKAP5, CD2AP, SGO2, CDC34, CCNE2, KIF2A, BIRC6, CEP63, KIF20B, USP16, RGS14, GNAI3, NCAPG, PDS5B, BRCC3, PDS5A, ASUN, KATNA1, RAD21, TPR, RBBP8, EVI5, UBE2I, SPAG5, USP9X, WAPL, PPP1CA, CENPE, TPX2, STAG1, CENPF, STAG2, CDK5, FAM175B, CENPJ, CDK1, CDK10, PKN2, SPG20
cellular response to DNA damage stimulus	1.78E-07	2.316890699	TOP2A, BLM, USP16, MCM8, STXBP4, FMR1, C9ORF142, BOD1L1, ZBTB1, CIB1, SMC5, SMC6, CHD2, TANK, CCAR2, NIPBL, RASSF1, WRN, CHCHD6, UBR5, TLK1, RBBP6, TOPBP1, APBB1, USP47, TIGAR, ARMT1, BAZ1B, SIRT1, WDR76, TOPORS, SETX, KIN, RAD50, APC, IRF3, TAOK3, TAOK1, PSME4, SPRTN, WAC, TRIP12, RAD18, ATR, OTUB1
transcription, DNA-templated	3.79E-07	1.347549455	RB1, ZNF175, JMJD1C, CCAR2, IKZF5, PNN, ZMIZ2, GPBP1, ZNF845, EPC2, ZNF600, TRIM27, ZNF721, SOX7, HOXA4, TSPYL2, SMARCC1, SFMBT1, ZNF160, ZNF281, CSNK2A2, DNTTIP2, EBF4, ZNF14, THAP7, HOXB4, VOPP1, SRFBP1, VPS25, CLOCK, ATF6, PRR13, ZNF277, ZNF28, URI1, ZNF273, MYCBP2, CTR9, SUPT4H1, BAZ2B, HIF1A, ZNF708, MIER3, DHX36, MIER1, ZSCAN12, ZNF267, ZSCAN18, ZNF140, MTERF2, NR2F1, SMARCA1, MOV10, ZZZ3, ZNF37A, TRIP6, ZNF813, ZNF702P, CREBRF, HBP1, RBAK, ZNF254, PHF3, ZNF253, ZNF131, ZNF493, ADIRF, ZNF43, ZNF808, ZMYM2, MECOM, HEY1, ZNF486, BATF2, ZNF121, PBRM1, ZNF480, PPHLN1, BAZ1A, BAZ1B, ZNF57, DYNLL1, SIRT1, ZFX, UCHL5, TOPORS, AES, MED22, GCFC2, ZEB1, SLTM, TFEC, WAC, ZNF117, NCOA7, ZNF350, ZNF518B, TXLNG, ARNTL2, RXRB, BCLAF1, ZNF107, PCGF1, ZNF347, ZNF468, APBB1, BRD7, TCEAL9, ZNF100, SUZ12, MCRS1, ATAD2, GTF2H1, LRPPRC, ZNF33A, RYBP, ZNF70, RLIM, TCEA1, PKN2, PKN1, ZNF354A, ZNF354B, ZNF451, ZNF570, ARID4B, RORA, AHR, BZW1, ZNF208, ZNF326, ZNF689, CIR1, KMT5B, ZNF688, ARID2, MAP3K7, ZNF440, ZBTB33, POLRMT, HIC1, ZNF91, ZNF92, ZNF93, DMTF1, TBL1XR1, MORF4L2, ZNF439, ZNF799, ZNF675, ZNF432, ZNF431, KMT2E, ZNF791, INO80E, DDX1, NPAT, ZNF876P, HNRNPDL, BPTF, ZNF782, ZNF660, CBX7, DENND4A, ZBTB11, CNOT2, TADA3, BRWD1, ZNF654, NFE2L2, ZNF512B, ZNF770, PHTF2, DDX3X, LDB1, CHD9, CHD7, CHD2, CHD1, MYSM1, PHTF1, LBH, VPS72, RB1CC1, ZNF407, ZNF644, LRRFIP1, IFT57, ZNF761, RFC1, EED, ATRX, EDF1, ZNF75A, DNAJC2, ELF2, ZNF638, ZNF517, LCOR, BDP1, ZNF510, BIRC2, HDAC5, GTF3C2, GTF3C3, PRKAA1, HDAC2, USP16, ZNF195, GTF3C5, ZBTB45, ZBTB41, IWS1, CAND2, TFPT, TAF1B, TGS1, NRIP1, ERBB2, POLR2E, POLR2B, POLR2F, POLR2G, ASCC3, POLR2I, E2F6, ZNF189, RBM39, ZNF184, MACC1, CCDC59, CDC5L, TBX4, TBX2, PNRC2, POLR3B, POLR3C, ZNF616, ZNF615, ZNF614, ESF1, TRIM37, NAA15, ZNF732, PAXBP1, TRIM33
cell-cell adhesion	3.8E-07	2.094415995	TES, DDX3X, RPL34, OLA1, FNBP1L, CLINT1, BZW1, SLK, SEPT7, RARS, ERC1, LRRFIP1, EIF2A, SH3GLB1, CAST, USP8, SWAP70, GAPVD1, CKAP5, EPN2, SHTN1, RAB1A, NOTCH3, TXNDC9, USO1, TWF1, CAPG, IQGAP1, KIAA1524, SDCBP, SNX2, CALD1, COBLL1, MYO6, EPS8L2, HIST1H3I, CCT8, ZC3H15, HSPA5, IDH1, RDX, MPP7, RAB11B, KTN1, ANLN, RSL1D1, EIF5, FMNL2, SERBP1, CHMP2B, PKN2, DHX29, PICALM
regulation of transcription, DNA-templated	4.53E-07	1.40273217	ZNF175, NUP107, JMJD1C, CCAR2, IKZF5, PNN, SOX17, GPBP1, ZNF845, ZNF600, ZNF721, ZNF720, HOXA4, PKNOX2, TSPYL2, SFMBT1, ZNF160, CSNK2A2, DNTTIP2, EBF4, ZNF14, CARHSP1, VOPP1, SRFBP1, VPS25, CLOCK, PRR13, ZNF277, ZNF28, ZNF273, MYCBP2, CTR9, SUPT4H1, BAZ2B, HIF1A, ZNF708, MIER3, ZSCAN12, ZNF146, ZNF267, ZRANB2, ZNF140, MTERF2, MOV10, ZZZ3, ZNF37A, GOLGB1, TRIP6, ZNF813, ZNF702P, CREBRF, HBP1, RBAK, ZNF254, ZNF253, ZNF131, SETD2, ZNF493, ZNF43, RSF1, ZNF808, MECOM, HEY1, ZNF486, ZNF121, PBRM1, ZNF480, PPHLN1, BAZ1A, BAZ1B, ZNF57, DYNLL1, ZFX, UCHL5, PHF20L1, GCFC2, TFEC, ZNF117, TMF1, ZNF350, TXLNG, ARNTL2, RXRB, PPP2CA, ZNF107, PCGF1, DMD, ZNF347, ZNF468, APBB1, SRSF10, ZNF100, SUZ12, BRF1, MCRS1, ATAD2, HIPK3, LRPPRC, ZNF33A, ZNF70, RLIM, PKN2, ZNF354A, ZNF354B, ZNF570, ARID4B, RORA, AHR, BZW1, ZNF208, ZNF689, KMT5B, ZNF688, ARID2, PDE8A, PTRF, ZNF440, HIC1, ZNF91, ZNF92, ZNF93, DMTF1, MORF4L2, ZNF439, ZNF799, ZNF675, ZNF432, ZNF431, ZNF791, INO80E, DDX1, SBNO1, ZNF876P, HNRNPDL, BPTF, ZNF782, ZNF660, DENND4A, ZBTB11, CNOT2, ZNF654, ZNF512B, PHTF2, LDB1, CHD9, USP34, CHD7, CHD2, AFF4, PHTF1, LBH, SNAPC2, RB1CC1, ZNF407, LRRFIP2, ERC1, ZNF644, LRRFIP1, IFT57, ZNF761, ATRX, EDF1, ZNF75A, IRF3, ZNF638, ZNF517, LCOR, PFDN5, ZNF510, IRF9, BIRC2, PRKAA1, ZNF195, ZBTB45, RNF6, ZBTB41, MLLT6, TFPT, TAF1D, TAF1B, TAF1C, TGS1, ASCC3, ZNF189, RNF20, RBM39, ZNF184, TAF10, MACC1, CCDC59, TBX4, PNRC2, ZNF616, ZNF615, ZNF614, ESF1, ZNF732
ubiquitin-dependent protein catabolic process	4.43E-06	2.235983404	USP14, USP37, USP15, USP38, USP16, CUL5, PSMD14, UBA6, USP32, CUL3, CUL2, USP12, USP34, UBR3, RNF6, UBE3A, USP19, UBR5, USP1, ARIH1, RNF20, USP25, USP8, USP47, USP7, UBE2I, USP9X, USP3, USP45, FBXO11, TOPORS, UCHL5, COPS3, ITCH, PSMC6, TTC3, RLIM, UBE2K
DNA duplex unwinding	5.6E-06	3.894248609	BLM, ANXA1, DDX3X, DHX9, DDX1, ATRX, RECQL, CHD2, CHD1, SETX, BRIP1, WRN, RAD50, DNA2, NBN, ASCC3
protein sumoylation	7.61E-06	2.562881563	TOP2A, TOP2B, ZNF451, BLM, NUP107, SMC5, SMC6, SMC3, SENP6, WRN, RAD21, TPR, NUP88, TRPM4, RANBP2, NOP58, UBE2I, XRCC4, NUP155, NSMCE2, NUP153, SMC1A, TOPORS, SENP1, STAG1, STAG2, MDM2, TOP1
mRNA splicing, via spliceosome	1.65E-05	2.026061776	SF3B5, DDX46, DHX9, CSTF3, HNRNPU, HNRNPR, CWC27, PNN, SYNCRIP, METTL14, FIP1L1, PCF11, POLR2B, RSRC1, CWC22, DHX15, PAPOLA, POLR2E, POLR2F, POLR2G, POLR2I, SRSF10, SF3B1, SRSF11, SF3A3, AQR, CPSF3, UPF3B, CDC5L, PRPF4B, PRPF40A, PLRG1, LSM4, LSM2, HNRNPH1, HNRNPA2B1, RBM41, SRSF4, PABPC1, HNRNPH3, SLU7, SKIV2L2
cilium morphogenesis	4.89E-05	2.28357593	RAB1A, RPGR, IFT74, PCM1, SEPT7, ARL13B, TBC1D32, TTC21B, IFT57, ATP6V1D, CEP290, C5ORF42, RAB8B, DYNC2H1, IQCB1, BBS1, WDR19, IFT80, RPGRIP1L, DYNLL1, RSG1, AHI1, IFT88, CFAP221, TMEM138, KIAA0586, TMEM216, CEP83, SSX2IP
RNA splicing	5.74E-05	2.128934104	RBM25, FMR1, CCAR2, IWS1, PPP2CA, LSM10, SYNCRIP, PPP4R2, RSRC1, DHX15, CIR1, ZNF326, SRSF11, SF3A3, RBM39, PRPF38B, ZRANB2, SCAF11, THOC1, SREK1IP1, PRPF4B, THOC2, SCAF1, LSM4, SRPK1, ZNF638, MPHOSPH10, LUC7L3, SRSF4, PPIG, SREK1, HNRNPH3, NSRP1
cilium assembly	6.5E-05	2.331838381	CBY1, FNBP1L, RPGR, IFT74, PCM1, ARL13B, IFT57, ATP6V1D, CEP290, C5ORF42, DYNC2H1, IQCB1, BBS1, CEP295, WDR19, RAB3IP, IFT80, RPGRIP1L, RSG1, AHI1, TMEM138, KIAA0586, TMEM216, ALMS1, CEP83, EXOC5, SSX2IP
double-strand break repair	8.45E-05	2.920686456	PARP3, XRCC4, DDX1, SMARCA5, LIG4, CIB1, ESCO2, BRCC3, BRCA2, BAZ1B, SETX, BRIP1, WRN, RAD50, TDP2, RAD21, APBB1, NBN
DNA replication	8.94E-05	2.141836735	BLM, INO80E, MCM8, NOL8, PTMS, ING4, POLD3, POLD4, BRIP1, WRN, CHTF8, RBBP8, RBBP6, TOPBP1, NBN, POLK, SUPT16H, RFC1, LIG4, NAP1L1, SIRT1, CCDC88A, KIN, DNAJC2, RAD50, NASP, CDK1, RBMS1, DNA2, TOP1, ATR
mitotic nuclear division	0.000106	1.856834266	USP37, USP16, RGS14, DCTN3, CLTC, SMC5, BRCC3, SNX33, KIF11, SMC3, SEPT7, MIS18BP1, ASUN, KATNA1, RAD21, TPR, RBBP8, KNTC1, NUP88, FBXO5, LZTS2, LRRCC1, PBRM1, UBE2I, PPP1R12A, USP9X, NSMCE2, KNL1, TERF1, CKAP5, CD2AP, ASPM, ANLN, TPX2, STAG1, CENPF, CLIP1, STAG2, TADA3, CDK1, BIRC6, CEP63, KIF20B
COPII vesicle coating	0.000107	2.984526598	ANKRD28, RAB1A, TRAPPC2L, SEC24A, CUL3, RAB1B, USO1, TRAPPC5, LMAN1, TRAPPC6A, SCFD1, SEC23IP, TRAPPC6B, PPP6R3, SEC24D, FOLR1, SEC31A
mitotic sister chromatid cohesion	0.000116	6.119533528	NIPBL, CHTF8, RAD21, PDS5B, SMC1A, SMC3, PDS5A, WAPL
transcription-coupled nucleotide-excision repair	0.000118	2.749655268	USP7, AQR, RFC1, GPS1, XPA, XRCC1, GTF2H1, COPS4, POLD3, POLD4, COPS3, POLR2B, COPS2, POLR2E, TCEA1, POLR2F, POLR2G, POLK, POLR2I
sister chromatid cohesion	0.000174	2.391371112	RANBP2, NUP107, ESCO1, SMC5, PDS5B, KNL1, SMC3, PDS5A, SMC1A, CKAP5, WAPL, CENPE, SGO2, STAG1, CENPF, CLIP1, KIF18A, STAG2, KIF2A, TAOK1, RAD21, KNTC1, SPDL1
DNA synthesis involved in DNA repair	0.000228	3.671720117	POLD3, POLD4, BLM, BRIP1, WRN, RAD50, RBBP8, DNA2, NBN, BRCA2, PALB2, SIRT1
regulation of DNA replication	0.000326	5.354591837	ANKRD17, PPP2CA, CCDC88A, ESCO1, NUCKS1, RBBP6, ESCO2, SMC3
endosome organization	0.000461	3.681281888	USP8, FAM160A2, ALS2CL, HOOK1, CHMP2B, KIAA1033, PIK3C3, SNX33, HOOK3, LAMTOR1, PLEKHJ1

RNA processing	0.000548	2.318483063	RBM39, SSB, RNGTT, DHX9, CSTF3, THOC1, DDX20, ZFC3H1, HNRNPU, TRMT2A, LSM4, U2SURP, SETX, SYNCRIP, HNRNPDL, HNRNPH1, DHX36, RBMS1, MPHOSPH10, DHX29, HNRNPH3
transcription from RNA polymerase III promoter	0.000606	3.569727891	GTF3C2, ZNF143, GTF3C3, GTF3C5, POLR3B, BRF1, SNAPC2, POLR3C, POLR2E, POLR3F, POLR2F
NLS-bearing protein import into nucleus	0.00077	4.190550133	RANBP2, DAG1, KPNA4, TNPO1, RANBP6, KPNA2, KPNA3, IPO5, KPNA1
chromatin remodeling	0.000844	2.365982439	RB1, HDAC5, SMARCC1, PBRM1, HDAC2, ATRX, CHD7, ZBTB1, SUPT4H1, SMARCA5, RSF1, BAZ1A, DEK, SMARCA1, CHD1, MYSM1, MORF4L2, TOP1, BPTF
I-kappaB kinase/NF-kappaB signaling	0.001022	2.677295918	RNF31, ROCK1, CHUK, ROCK2, HACD3, TANK, TBK1, NKIRAS1, REL, TAB3, TAB2, AZI2, ZNF675, MAP3K7, BIRC2
covalent chromatin modification	0.00165	2.084973813	RB1, TSPYL2, CBX7, SMARCC1, PBRM1, SFMBT1, CHD9, ATRX, CHD7, SMARCA5, RSF1, DEK, SMARCA1, CHD2, CHD1, DNAJC2, VPS72, MTF2, ANP32E, TLK1, ARID2, BPTF
protein transport	0.001704	1.518264014	DENND1B, KIAA1033, SNX12, MIA3, PHAX, ARRB2, NXT2, AP4M1, ERC1, CEP290, TMED5, TMED4, CADPS2, DENND2A, SCAMP3, LSG1, RABEP1, ATG4A, EXOC5, ARF5, RAMP2, RAB1B, GOSR1, NMD3, SDAD1, HSP90B1, MTM1, SNX4, LMAN1, SCFD1, RAP1A, AP5M1, VPS50, MVB12A, VPS54, STXBP5, SLC15A3, ECT2, SEC31A, ARFGEF1, DENND4C, LCA5, HOOK3, RSG1, KTN1, UEVLD, KIF18A, NECAP2, CENPF, FAM160A2, NASP, HOOK1, CHMP2B, RAB18, CCDC22, RAB5A
mRNA 3'-end processing	0.001771	2.784387755	POLDIP3, CSTF3, CPSF3, THOC1, UPF3B, THOC2, FIP1L1, PCF11, ZC3H11A, PAPOLA, SRSF4, SLU7, SRSF11
G2/M transition of mitotic cell cycle	0.001864	1.954230597	CEP57, DCTN3, CCP110, HMMR, PPP1CB, PCM1, CEP70, CNTRL, CEP290, PLK4, HSP90AA1, PPP1R12A, CEP135, CEP152, DYNLL1, TERF1, CKAP5, TPX2, MELK, CENPJ, AKAP9, ALMS1, CDK1, CEP63, TAF2
strand displacement	0.001904	3.707025118	BLM, BRIP1, WRN, RAD50, RBBP8, DNA2, NBN, BRCA2, PALB2
termination of RNA polymerase II transcription	0.001982	2.509964923	POLDIP3, CSTF3, CPSF3, THOC1, UPF3B, THOC2, LSM10, SETX, FIP1L1, PCF11, ZC3H11A, PAPOLA, SRSF4, SLU7, SRSF11
telomere maintenance via recombination	0.002026	3.346619898	POLD3, POLD4, WRN, RAD50, RFC1, NSMCE2, SMC5, DNA2, SMC6, BRCA2
mRNA export from nucleus	0.002062	2.141836735	UPF2, RANBP2, SMG1, NUP107, NUP155, POLDIP3, CPSF3, THOC1, UPF3B, NUP153, THOC2, FIP1L1, ZC3H11A, TPR, HNRNPA2B1, SRSF4, NUP88, SLU7, SRSF10, SRSF11
proteasome-mediated ubiquitin-dependent protein catabolic process	0.002244	1.740901779	PSMD12, UBXN2B, PSMD14, CUL3, KIF14, RNF6, ARRB2, PCNP, RMND5B, PSMD8, PSMB4, CDC27, PSMD1, EDEM3, WWP1, SIRT1, TOPORS, PSMB9, CD2AP, ABTB1, PSMC6, CDC34, APC, TBL1XR1, PSME4, CDK1, RLIM, PLAA, SPOPL, UBE2K, CLOCK, BIRC2, NFE2L2
mRNA processing	0.002326	1.794835253	TSEN34, RBM27, RBM25, FMR1, HNRNPR, CCAR2, TSEN54, IWS1, LSM10, PPP4R2, DHX15, CIR1, ZNF326, RBBP6, SMNDC1, SRSF11, SF3A3, RBM39, PRPF38B, ZRANB2, SCAF11, SREK1IP1, SCAF1, PTBP2, SRPK1, KIN, HNRNPA2B1, SRSF4, SREK1, NSRP1
protein folding	0.002532	1.784863946	ERO1A, HSP90AA2P, HSP90AA4P, FKBP14, GNAI3, LTBP4, CWC27, HSP90B1, DNAJB2, LMAN1, DNAJC21, SACS, CCT8, RANBP2, HSPA9, HSP90AB3P, CCT2, HSP90AA1, CSNK2A2, AIP, ERP44, DNAJC7, TMX1, GNB2, TCP1, FKBP1B, PPIG, PPIL4, ATF6, PFDN5
positive regulation of intracellular protein transport	0.002949	3.894248609	PCM1, ICE1, ASPH, RUFY3, TPR, PRR5L, KIF20B, CEP290
ER-associated ubiquitin-dependent protein catabolic process	0.003095	2.498809524	EDEM3, HSPA5, WFS1, CCDC47, YOD1, USP19, HSP90B1, DNAJB2, ERLEC1, PSMC6, KIAA0368, DNAJC10, MAN1B1, UBXN4
vesicle organization	0.003202	3.442237609	SNX4, SNX2, ZFYVE16, SNX12, SNX33, FBXO5, FNBP1L, SNX6, CD2AP
regulation of establishment of cell polarity	0.003278	4.409663866	ARFGEF1, SHTN1, ROCK1, ROCK2, RUFY3, RICTOR, KIF20B
cell cycle	0.003473	1.677936612	USP16, RIF1, GPS1, BCCIP, GNAI3, TXLNG, AHR, CCAR2, CDC73, CKS1B, PCNP, RPS6KA3, RB1CC1, GPER1, TLK1, MAPK6, BRD7, EVI5, APPL1, TSPYL2, ERBIN, LIG4, APEX2, PRPF40A, ESCO2, BLCAP, CTCFL, PPP1CA, DMTF1, NASP, CDK5, PIN1, PKN2, ATR
DNA recombination	0.003862	2.193447258	BLM, INO80E, XRCC4, POLM, MCRS1, ATRX, RECQL, APEX2, LIG4, RBPJ, UCHL5, SETX, KIN, TFPT, WRN, RAD50, RAD21
cytokinesis	0.003992	2.677295918	DIAPH2, SEPT7, ROCK2, PRC1, DCTN3, KIF23, PIK3C3, BRCA2, RHOC, ECT2, LZTS2, MAP9
retrograde transport, endosome to Golgi	0.004128	2.328083407	SPAG9, DENND2A, GOSR1, CLTC, GCC2, VPS26A, PIKFYVE, SNX2, VPS50, VPS54, TRIM27, ERC1, EVI5, SNX6, PLEKHJ1
early endosome to late endosome transport	0.004533	4.16468254	EEA1, FAM160A2, HOOK1, KIF16B, PIK3C3, HOOK3, RAB5A
microtubule anchoring	0.004775	4.942700157	CEP57, PCM1, CEP350, DAG1, GCC2, PEX14
regulation of translational initiation	0.004842	2.974773243	EIF5B, EIF3M, EIF5, EIF3K, DDX1, EIF3J, EIF3H, EIF4G3, EIF3A, EIF4B
protein K48-linked deubiquitination	0.005071	3.569727891	USP25, USP8, USP37, USP34, FAM63A, YOD1, USP19, OTUB1
regulation of signal transduction by p53 class mediator	0.005213	1.900016458	BLM, PRKAA1, HDAC2, SUPT16H, TAF10, CSNK2A2, TPX2, BRIP1, WRN, RAD50, CDK5, JMY, MDM2, TRIM24, RBBP8, PIN1, TOPBP1, DNA2, NBN, BRD7, TAF2, ATR
negative regulation of protein kinase B signaling	0.005879	2.894373966	DLG1, BANK1, GPER1, PRKCDBP, DAG1, CIB1, ARRB2, SIRT1, LEMD2, MTM1
RNA splicing, via transesterification reactions	0.006486	3.426938776	SF3A3, SCAF11, MPHOSPH10, SRSF4, SRSF10, SLU7, SF3B1, SMNDC1
viral entry into host cell	0.0066	2.141836735	SCARB1, CXADR, SIVA1, CD81, WWP1, NUP153, IDE, DPP4, ACE2, ITCH, GRK2, DAG1, ITGAV, TNFRSF14, GAS6, KPNA3
regulation of DNA-templated transcription, elongation	0.006863	4.589650146	LDB1, THOC1, TCEA1, ZNF326, CCAR2, IWS1
interkinetic nuclear migration	0.007021	8.567346939	PCM1, DOCK7, CEP120, HOOK3
intracellular protein transport	0.007142	1.588226392	COPB2, RAMP2, UNC93B1, STX19, COPB1, USO1, CLTC, SNX13, VPS26A, SNX33, IPO7, IPO5, RPGR, AP4M1, SNX2, GGA3, MYO6, TLK1, TNPO1, EVI5, ANKRD50, TBC1D15, SNX6, SEC24A, AP3B1, SYTL2, TOM1L1, SEC23IP, CDK5, SNX17, IFT27, RAB3GAP2, SEC24D, SLU7, STAM2
bicellular tight junction assembly	0.007751	3.011957908	RAMP2, DLG1, APC, STRN, ECT2, PTPN13, MPP5, MPP7, MTDH
small GTPase mediated signal transduction	0.007779	1.567197611	RASL12, RAB1A, RAB1B, DOCK7, RASEF, RAP1GDS1, ITSN1, HACD3, PLD2, DOCK10, DOCK11, RAP1A, RRAS, ARL13B, NKIRAS1, RAC2, HRAS, RAB8B, RALGPS2, SH2D3C, RAB27A, RHOC, RSG1, RAB11B, ARL4A, RAB18, RABL3, IFT27, RAPGEF2, KRAS, RAPGEF6, RGL2, RAB5A, SOS2, RHOQ, ARF5
Golgi organization	0.007861	2.170780474	ARFGEF1, DYNC2H1, RAB1A, UBXN2B, USO1, DNAJC28, SYNE1, ARHGAP21, LMAN1, SEC23IP, GOLGA5, GOLGB1, TRIP11, KIFC3, BLZF1
double-strand break repair via homologous recombination	0.007861	2.170780474	GEN1, BLM, PSMD14, MCM8, NSMCE2, RECQL, XRCC1, SMC5, NUCKS1, SMC6, BRCA2, PALB2, RAD50, RBBP8, NBN
mitochondrial electron transport, cytochrome c to oxygen	0.008049	3.748214286	COX8A, COX7A2L, COX4I1, COX4I2, COX5B, COX6A1, COX6B1
translational initiation	0.008157	1.79789215	EIF5B, EIF1AX, RPS5, RPL34, RPL12, EIF2S2, EIF2S1, RPS17, EIF3M, EIF5, EIF3K, RPL18A, EIF3J, RPL35, EIF3H, DHX29, PABPC1, ABCE1, EIF4G3, RPS21, EIF3A, EIF4B, EIF2A
Wnt signaling pathway	0.008291	1.660782495	PRKAA1, DDX3X, LDB1, NXN, CUL3, USP34, CTR9, VPS26A, RTF1, CCAR2, CDC73, TNKS2, LEO1, RSPO3, LRRFIP2, LZTS2, BRD7, ZRANB1, DST, CSNK2A2, WNT7A, ZBTB33, WNT9A, HIC1, AES, PPM1A, APC, HBP1, STRN
retrograde vesicle-mediated transport, Golgi to ER	0.008317	2.089596814	RAB1A, COPB2, COPB1, PITPNB, RAB1B, KIF23, KIF11, DNAJC28, CENPE, KIF18A, SCFD1, RACGAP1, RER1, KIF2A, ERGIC2, ARF5
chromosome segregation	0.00936	2.204831933	TOP2A, UBE2I, DDX3X, RGS14, SPAG5, RIOK3, USP9X, ESCO2, KIF11, SRPK1, CENPE, CENPF, FAM175B, TOP1
mitotic G2 DNA damage checkpoint	0.009515	4.283673469	FANCI, BLM, TAOK3, CDK1, TOPBP1, NBN
protein deubiquitination	0.009803	1.947124304	USP25, USP14, USP8, USP7, USP15, USP37, USP38, USP53, USP9X, USP32, USP3, USP12, USP34, USP45, UCHL5, USP19, USP1, OTUB1
striated muscle cell differentiation	0.010015	5.354591837	SPAG9, RB1, CHUK, BNIP2, KRAS
positive regulation of gene expression, epigenetic	0.011137	2.245473996	SMARCA5, DDX21, DEK, BAZ1B, TAF1D, MYO1C, TAF1B, TAF1C, POLR2E, POLR2F, HIST1H3I, HIST1H4C, SF3B1
gene expression	0.011849	2.454187925	METTL14, HNRNPH1, POLR2B, HNRNPA2B1, POLR2E, HNRNPU, POLR2F, POLR2G, HNRNPR, FMN1, POLR2I
mRNA polyadenylation	0.012484	3.059766764	CPSF4, PCF11, CSTF3, CPSF3, LEO1, PAPOLA, PABPC1, CDC73
protein dephosphorylation	0.012765	1.784863946	MTMR2, PPP1R12A, PPP2R3B, PTPRK, PPP4R3B, CTDSPL2, PTPN12, PTPN13, STYXL1, MTMR6, MTM1, PPP1CA, PPP2CA, PPP1CB, PPM1A, DLG1, PPM1B, UBLCP1, PHPT1, DLGAP5, LHPP

centriole replication	0.012793	4.015943878	PLK4, CEP135, CEP152, CENPJ, CCP110, CEP63
spindle organization	0.012793	4.015943878	ASPM, SPAG5, RGS14, TTK, KIF11, CKAP5
transcription elongation from RNA polymerase II promoter	0.012808	1.992406265	SETD2, SUPT16H, TAF10, SUPT4H1, GTF2H1, RTF1, CDC73, POLR2B, LEO1, POLR2E, TCEA1, TCEB2, POLR2F, POLR2G, POLR2I, TAF2
viral process	0.012878	1.46848338	ANKRD17, RB1, NCKAP1, NUP107, CUL5, COPB1, CUL2, UBE3A, IPO7, HSPD1, IPO5, SYNCRIP, PSMB4, TPR, POLR2E, NUP88, TNPO1, MPDZ, MAP3K5, RANBP2, USP7, ACOT8, RNGTT, UBE2I, NUP155, NUP153, CFLAR, VRK2, DYNLL1, SIRT1, WAPL, PSMB9, SRPK1, KIN, DLG1, RAD50, MDM2, TOP1, ABCE1, IL6ST, EIF4G3
positive regulation of mitotic metaphase/anaphase transition	0.013071	7.139455782	RB1, CUL3, NSMCE2, DLGAP5
positive regulation of nonmotile primary cilium assembly	0.013071	7.139455782	WRAP73, SEPT7, CEP135, CENPJ
endodermal cell fate commitment	0.013071	7.139455782	CTR9, LEO1, RTF1, CDC73
natural killer cell degranulation	0.013071	7.139455782	VAMP7, RAB27A, CORO1A, UNC13D
regulation of cytokinesis	0.013188	3.407467532	SH3GLB1, PIN1, CCP110, BIRC6, PIK3C3, PRPF40A, BRCA2
response to X-ray	0.013188	3.407467532	BLM, ANXA1, XRCC4, CASP3, LIG4, BRCC3, BRCA2
ER to Golgi vesicle-mediated transport	0.013486	1.673309949	RAB1A, COPB2, CUL3, RAB1B, COPB1, USO1, GOSR1, DCTN3, MIA3, LMAN1, SEC31A, DYNC2H1, TRAPPC2L, SEC24A, F10, DYNLL1, TRAPPC6A, SEC23IP, VAMP7, TRAPPC6B, GOLGB1, MPPE1, SEC24D, GAS6, FOLR1
histone monoubiquitination	0.01459	4.867810761	RNF20, CTR9, LEO1, WAC, CDC73
nuclear export	0.01459	4.867810761	LSG1, NEMF, PHAX, LZTS2, MALT1
circadian regulation of gene expression	0.015059	2.254564984	PPP1CB, HDAC2, GFPT1, NRIP1, PRKCDBP, HNRNPU, RORA, AHR, TOP1, SIRT1, CLOCK, PPP1CA
mitotic cytokinesis	0.015163	2.954257565	ANLN, USP8, APC, RACGAP1, NUSAP1, CKAP2, KIF23, SNX33
telomere maintenance	0.015987	2.677295918	POLD3, POLD4, PARP3, SMG1, WRN, RAD50, DNA2, NBN, TERF1
mitotic spindle assembly	0.015987	2.677295918	WRAP73, TPX2, KIF2A, FAM175B, CLTC, KIF11, PIBF1, BIRC2, MAP9
cellular senescence	0.016466	3.25931677	C2ORF40, OPA1, NSMCE2, SMC5, SMC6, HRAS, TBX2
formation of translation preinitiation complex	0.016466	3.25931677	EIF3M, EIF5, EIF3K, EIF3J, EIF3H, EIF3A, EIF4B
protein K63-linked deubiquitination	0.016466	3.25931677	USP25, USP8, ZRANB1, PSMD14, FAM175B, YOD1, BRCC3
microtubule-based movement	0.017087	1.983182162	DYNC2H1, KIF14, KIF23, KIF11, KTN1, CENPE, KIF18A, RACGAP1, KIF2A, KIF16B, KIF13B, KIF21A, KIFC3, KIF20B, DNAL4
intracellular transport of virus	0.017951	2.30982393	RANBP2, NUP107, NUP155, TPR, KPNA4, NUP153, NUP88, KPNA2, KPNA3, VPS28, KPNA1
intrinsic apoptotic signaling pathway	0.018226	2.855782313	DDX3X, CUL5, SIVA1, BAD, CUL3, CUL2, NBN, HRAS
mitotic spindle organization	0.018226	2.855782313	GPSM2, STIL, KIF2A, ASUN, TTK, KIF11, SMC1A, SMC3
RNA secondary structure unwinding	0.018585	2.43390538	DDX18, DDX3X, DDX46, AGO3, DDX1, DHX36, DDX20, DDX21, DDX10, DDX52
fibroblast growth factor receptor signaling pathway	0.018892	1.958997013	CEP57, GALNT3, FRS2, PTPN11, IQGAP1, SETX, HNRNPH1, POLR2B, KIF16B, POLR2E, POLR2F, SHOC2, POLR2G, FGFR4, POLR2I
cortical actin cytoskeleton organization	0.020264	3.123511905	DLG1, FMNL2, CDK5, ROCK1, ROCK2, PRKCDBP, RHOQ
response to endoplasmic reticulum stress	0.020617	1.999047619	ERO1A, WFS1, EIF2AK3, EIF2S1, USP19, HSP90B1, UFL1, ERP44, COL4A3BP, UFM1, TMX1, DNAJC10, CREBRF, MAP3K5
modulation by virus of host process	0.0213	6.119533528	KPNA4, KPNA2, KPNA3, KPNA1
intraciliary transport involved in cilium morphogenesis	0.0213	6.119533528	IFT74, PCM1, TTC21B, SSX2IP
atrioventricular canal development	0.0213	6.119533528	CHD7, PTPN11, RBPJ, TBX2
protein localization to cytoplasmic stress granule	0.0213	6.119533528	SSB, DDX3X, DHX9, DDX1
regulation of attachment of spindle microtubules to kinetochore	0.0213	6.119533528	APC, RACGAP1, SPAG5, ECT2
protein polyubiquitination	0.02134	1.5714563	RNF31, PSMD12, LRSAM1, PSMD14, CUL3, PSMD8, PSMB4, TNKS2, RNF19A, RNF217, UBR5, PSMD1, RBBP6, ARIH1, RNF20, ANKIB1, TOPORS, PSMB9, DDB2, RNF167, PSMC6, CDC34, PSME4, RLIM, TPP2, TRIP12, BIRC2
endoplasmic reticulum unfolded protein response	0.021366	2.379818594	ERO1A, EDEM3, HSPA5, WFS1, EIF2AK3, YOD1, UGGT2, UGGT1, ATF6, NFE2L2
protein localization to centrosome	0.02146	3.569727891	PCM1, STIL, SPAG5, CEP83, HOOK3, PIBF1
peptidyl-serine phosphorylation	0.022809	1.713469388	SMG1, DMPK, DYRK1A, EIF2AK3, TTK, VRK2, VRK3, HIPK3, TBK1, GRK2, CDK5, PRKD3, CDK1, STK38L, PRKD2, RICTOR, PKN2, PKN1, GAS6, ATR
resolution of recombination intermediates	0.024487	10.70918367	GEN1, SMC5, SMC6
negative regulation of histone H2A K63-linked ubiquitination	0.024487	10.70918367	UBR5, TRIP12, OTUB1
regulation of Rap protein signal transduction	0.024487	10.70918367	RDX, TIMP2, KIF14
cell aging	0.024615	2.998571429	WRN, PDCD4, CDK1, PRELP, BRCA2, SIRT1, TBX2
mitotic cell cycle checkpoint	0.025603	2.677295918	RB1, DLG1, KNTC1, TTK, NBN, WNT9A, SMC1A, HRAS
positive regulation of I-kappaB kinase/NF-kappaB signaling	0.025948	1.596400051	RNF31, ANKRD17, UBE2I, CHUK, CFLAR, RHOC, ATP2C1, ZDHHC17, MALT1, MTDH, BST2, PPM1A, MAVS, TBK1, IRF3, MIER1, REL, S100A4, TAB2, CCDC22, ECT2, MAP3K7, BIRC2, TMED4
positive regulation of protein export from nucleus	0.026943	3.381847476	PPM1A, TPR, MDM2, CTDSPL2, ANP32B, GAS6
centrosome duplication	0.026943	3.381847476	STIL, ROCK2, CEP152, CCP110, BRCA2, CKAP5
protein ubiquitination involved in ubiquitin-dependent protein catabolic process	0.027085	1.609877284	ANKIB1, CUL5, UBA6, CUL3, CUL2, WWP1, RCHY1, FBXL14, RMND5B, ABTB1, ITCH, HECTD1, RNF19A, CDC27, RNF217, UBR5, KLHL8, MDM2, CDK1, SPOPL, TRIP12, RBBP6, ARIH1
regulation of transcription from RNA polymerase III promoter	0.027193	4.118916797	ZNF143, GTF3C3, POLR3C, POLR3F, BDP1
regulation of embryonic development	0.027193	4.118916797	NIPBL, SOX17, LAMA2, CDK1, NFE2L2
regulation of mRNA stability	0.028977	1.767535169	PSMD12, PSMD14, DIS3, FMR1, PSMB9, PSMD8, EXOSC6, CARHSP1, PSMB4, PSMC6, EXOSC4, XRN1, SERBP1, PSME4, PSMD1, PABPC1, TNPO1
positive regulation of viral genome replication	0.029547	2.883241758	TOP2A, DDX3X, PKN2, PABPC1, HACD3, TARBP2, SRPK1
7-methylguanosine mRNA capping	0.029962	2.596165739	RNGTT, POLR2B, RNMT, POLR2E, POLR2F, GTF2H1, POLR2G, POLR2I
double-strand break repair via nonhomologous end joining	0.030166	2.039844509	RAD50, PSMD14, XRCC4, RIF1, POLM, C9ORF142, NSMCE2, LIG4, SMC5, BRCC3, NBN, HIST1H4C
morphogenesis of an epithelial sheet	0.031747	5.354591837	DAG1, HOXB4, MPP5, ARHGAP12
cellular response to antibiotic	0.031747	5.354591837	HSPA5, MDM2, PLA2G4A, CRIP1
amyloid precursor protein catabolic process	0.031747	5.354591837	APH1A, PSENEN, CLN3, PSEN2
regulation of mast cell degranulation	0.031747	5.354591837	C12ORF4, FER, RAC2, UNC13D
histone H2B ubiquitination	0.031747	5.354591837	RNF20, CTR9, LEO1, CDC73
embryonic cleavage	0.031747	5.354591837	TOP2A, CUL3, PIK3CB, TOP1
N-glycan processing	0.03324	3.212755102	EDEM3, MAN1A2, MAN2A1, MAN1C1, MAN1B1, USF3
regulation of transcription from RNA polymerase II promoter	0.033253	1.311328613	RB1, ZNF770, ZNF131, ARID4A, ARID4B, NUCKS1, AHR, CHD2, CHD1, ZMYM2, EPC2, CIR1, LRRFIP1, SOX7, PKNOX2, BATF2, SMARCC1, WDR75, MED4, MED22, ZEB1, ELF2, DMTF1, SLTM, LCORL, ATF6, CLOCK, BLZF1, URI1, USP16, ZNF518B, SUPT4H1, ARNTL2, BRIP1, TTC21B, RAD21, NRIP1, RBBP8, BRD7, ZSCAN18, TCEAL9, ABCA2, ZNF143, SMARCA5, CDC5L, DEK, CNOT2, TADA3, JMY, TCEA1, PKN1, ZNF354A, BRWD1, BRWD3
mitotic cell cycle	0.033409	2.350796416	CENPE, FER, CENPF, CUL3, FAM175B, USP3, CNTRL, CDC5L, AZI2
protein processing	0.033611	1.933602608	DYNC2H1, PSENEN, NRDC, PSEN2, IDE, APH1A, CLN3, ADAMTS3, CPD, PMPCB, ATG4A, PIK3C3, METAP2

endocytosis	0.033679	1.617934224	ITSN2, RAB1A, SNX33, PIK3C2A, GAPVD1, FNBP1L, CLINT1, EPN2, MARCH2, EEA1, SNX4, NECAP2, RABEP1, SNX2, VAMP7, MYO6, DNM1L, HRAS, RAB5A, SNX6, PICALM
positive regulation of interferon-beta production	0.035086	2.776455026	MAVS, TBK1, DDX3X, POLR3B, IRF3, POLR3C, POLR3F
nonmotile primary cilium assembly	0.035086	2.776455026	BBS1, PCM1, FAM179B, ARL13B, CENPJ, IFT80, PIBF1
barbed-end actin filament capping	0.035324	3.824708455	EPS8, RDX, CAPZA2, TWF1, CAPG
positive regulation of transcription elongation from RNA polymerase II promoter	0.035324	3.824708455	SUPT16H, CTR9, LEO1, RTF1, CDC73
cellular response to hydrogen peroxide	0.036602	2.066684569	SETX, PRKAA1, HDAC2, ANXA1, MDM2, CDK1, ECT2, SIRT1, MAP3K5, ZNF277, NFE2L2
stem cell population maintenance	0.039835	2.141836735	METTL14, NIPBL, RIF1, MTF2, CTR9, LEO1, RTF1, SMC1A, SMC3, CDC73
positive regulation of exocytosis	0.04038	3.059766764	CADPS2, ATP6AP1, RAB27A, STXBP5, RAB5A, UNC13D
protein import into nucleus	0.040673	2.031052076	NUP107, NUP155, CSE1L, CFL1, TPR, NUP153, NUP88, KPNA2, IPO7, KPNA3, KPNA1
regulation of cell motility	0.04125	2.677295918	ROCK1, ROCK2, CD81, ERBB2, PKN2, PKN1, SSX2IP
rRNA processing	0.042309	1.451244516	LTV1, RPL34, RPL12, CHD7, DDX21, NOL8, WDR43, EXOSC6, RPS17, EXOSC4, RPL18A, BMS1, RPL35, RIOK2, RIOK1, NOP58, RRP7BP, UTP11, RIOK3, KRR1, DIS3, TEX10, RPS5, WDR75, DDX52, SIRT1, MPHOSPH10, RPS21, SKIV2L2
regulation of cellular response to heat	0.044279	1.856258503	RANBP2, HSP90AA1, NUP107, NUP155, NUP153, SIRT1, CCAR2, DNAJC2, DNAJC7, TPR, CHORDC1, NUP88, ATR
nucleic acid phosphodiester bond hydrolysis	0.044279	1.856258503	GEN1, DDX1, APEX2, ZC3H12C, WRN, RAD50, XRN1, TDP2, RBBP8, REV3L, DXO, DNA2, N4BP2
cullin deneddylation	0.044379	4.759637188	COPS4, COPS3, COPS2, GPS1
protein maturation by protein folding	0.044379	4.759637188	ERO1A, WFS1, FKBP1B, AIP
nuclear mRNA surveillance	0.044379	4.759637188	EXOSC6, EXOSC4, XRN1, DXO
DNA strand renaturation	0.044379	4.759637188	BLM, ANXA1, RECQL, SMARCA1
positive regulation of type I interferon production	0.044524	2.099839936	TBK1, POLR3B, IRF3, DHX9, POLR3C, DHX36, POLR2E, POLR3F, POLR2F, LRRFIP1
DNA double-strand break processing	0.04471	3.569727891	BLM, RAD50, RBBP8, DNA2, NBN
mitotic chromosome condensation	0.04471	3.569727891	NUSAP1, NCAPG, SMC4, SMC2, TTN
IRE1-mediated unfolded protein response	0.045043	1.996627465	DNAJC3, TSPYL2, HSPA5, FKBP14, WFS1, GFPT1, PPP2R5B, SSR1, SEC62, SEC31A, SEC63
antigen processing and presentation of exogenous peptide antigen via MHC class II	0.045671	1.746062555	DYNC2H1, SEC24A, DCTN3, CLTC, KIF23, KIF11, DYNLL1, CENPE, HLA-DMA, KIF18A, RACGAP1, KIF2A, CTSF, SEC24D, SEC31A
translesion synthesis	0.045938	2.379818594	POLD3, POLD4, UBA7, RFC1, ZBTB1, REV3L, SPRTN, POLK
mitochondrial tRNA processing	0.045956	8.031887755	TRMT10C, HSD17B10, KIAA0391
positive regulation of chromosome segregation	0.045956	8.031887755	SMC5, SMC6, RAD18
meiotic chromosome segregation	0.045956	8.031887755	SMC4, WAPL, SMC2
positive regulation of viral transcription	0.048055	2.584975369	POLR2B, POLR2E, SUPT4H1, POLR2F, POLR2G, RSF1, POLR2I
establishment or maintenance of cell polarity	0.048055	2.584975369	DLG1, FAT1, MAP7, FSCN2, RPGRIP1L, SPINT2, SYNE2
positive regulation of G1/S transition of mitotic cell cycle	0.048377	2.920686456	LSM10, ANKRD17, DDX3X, ANXA1, RDX, PLRG1
negative regulation of proteasomal ubiquitin-dependent protein catabolic process	0.048377	2.920686456	SDCBP, SMARCC1, WAC, CCAR2, SENP1, MTM1
regulation of macroautophagy	0.048465	2.190514842	SH3GLB1, ATP6V0B, CDK5, CASP3, VPS26A, CAPN1, ATP6V1D, ATP6V1C1, SNX6
gene silencing by RNA	0.052557	1.640145247	RANBP2, NUP107, NUP155, FMR1, NUP153, TARBP2, POLR2B, CNOT2, TPR, POLR2E, POLR2F, POLR2G, NUP88, HIST1H3I, PABPC1, HIST1H4C, POLR2I
protein phosphorylation	0.054877	1.268192803	FASTKD2, GMFG, CCNC, RPS6KA3, RPS6KA6, COL4A3BP, RSRC1, TLK1, RIOK2, RIOK1, SCYL2, EIF2A, MAP3K5, CHUK, CSNK2A2, DYRK1A, CASK, PRPF4B, IRAK3, VRK2, PIK3CA, PRKD3, PRKD2, PIK3C3, BIRC6, GAS6, PRKAA1, ROCK1, ROCK2, DGUOK, ERBB2, STK38L, MAP4K5, MAPK6, CDK17, PLK4, MAP3K2, DMPK, RIOK3, EIF2AK3, PHKB, CDC42BPA, HIPK3, SRPK1, FER, TEC, TAOK3, STK17B, TAOK1, WNK3, CDK10, PKN2, PKN1, FRK
mitochondrial translational termination	0.055215	1.743355482	MRPS26, MRPS11, MRPS18A, MRPL37, MRPL34, MRPL43, MRPL10, MRPL21, MRPL11, MRPL52, MRPL3, MRPL53, MRPL1, MTRF1L
centrosome localization	0.055349	3.346619898	RANBP2, NIN, ASUN, CCDC141, SYNE2
Notch receptor processing	0.055349	3.346619898	APH1A, DLL4, PSENEN, NOTCH3, PSEN2
negative regulation of type I interferon production	0.055512	2.498809524	MAVS, ITCH, TBK1, UBA7, IRF3, TAX1BP1, PIN1
positive regulation of establishment of protein localization to plasma membrane	0.055512	2.498809524	DLG1, RER1, ITGA3, GPER1, WNK3, CIB1, PLS1
nucleotide-excision repair, DNA damage recognition	0.057241	2.793700089	COPS4, COPS3, COPS2, GPS1, XPA, DDB2
snRNA transcription from RNA polymerase II promoter	0.058731	1.835860058	ZNF143, INTS12, ICE1, ICE2, POLR2B, SNAPC2, ASUN, POLR2E, POLR2F, PHAX, POLR2G, POLR2I
microtubule polymerization	0.059106	4.283673469	MAP7D3, CENPJ, FBXO5, CKAP5
intraciliary retrograde transport	0.059106	4.283673469	DYNC2H1, TTC21B, WDR19, DYNLL1
DNA ligation involved in DNA repair	0.059106	4.283673469	PARP3, XRCC4, C9ORF142, LIG4
protein localization to kinetochore	0.059106	4.283673469	CDK1, TTK, KNL1, SPDL1
positive regulation of protein import into nucleus, translocation	0.059106	4.283673469	MAVS, HSP90AA1, UBR5, CDK1
regulation of dendritic spine morphogenesis	0.059106	4.283673469	EPHA4, CDK5, CFL1, PPP1R9A
Sertoli cell development	0.059106	4.283673469	ATRX, SDC1, HSD17B4, FNDC3A
endoplasmic reticulum mannose trimming	0.059106	4.283673469	EDEM3, MAN1B1, UGGT2, UGGT1
ER overload response	0.059106	4.283673469	HSPA5, WFS1, CCDC47, EIF2AK3
ERBB2 signaling pathway	0.059136	2.254564984	USP8, HSP90AA1, CUL5, PIK3CA, ERBB2, ERBIN, KRAS, HRAS
cellular protein modification process	0.063043	1.631875607	UBL4A, ERO1A, USP25, ATG3, UBE2I, YES1, UBA7, UBA6, MCRS1, PLOD2, FBXO11, TTLL7, UEVLD, CDC34, PPP4R2, TTLL11
stimulatory C-type lectin receptor signaling pathway	0.063043	1.631875607	PSMD12, PSMD14, CHUK, ICAM2, MALT1, PSMB9, PSMD8, PSMB4, PSMC6, PSME4, PSMD1, TAB3, KRAS, TAB2, HRAS, MAP3K7
termination of RNA polymerase I transcription	0.063628	2.418202765	TAF1D, TAF1B, TAF1C, POLR2E, POLR2F, GTF2H1, PTRF
cell cycle arrest	0.06561	1.519033145	KMT2E, RB1, PRKAA1, CUL5, CUL3, CUL2, PPP2R3B, ING4, PPM1A, RASSF1, APC, JMY, HBP1, APBB1, NBN, LAMTOR1, KIF20B, GAS6, HRAS, SKIL
RNA export from nucleus	0.066898	1.947124304	NUP155, POLDIP3, ZC3H11A, TPR, THOC1, UPF3B, SRSF4, THOC2, SLU7, SRSF11
nucleotide-excision repair, DNA gap filling	0.066972	2.677295918	POLD3, POLD4, RFC1, XRCC1, LIG4, POLK
mRNA splice site selection	0.067223	3.149759904	SETX, YTHDC1, LUC7L3, SRSF10, PTBP2
actin cytoskeleton reorganization	0.067348	2.050694746	EPS8, MKLN1, FER, CXADR, ANXA1, RICTOR, PARVB, ATP2C1, CDC42BPA
de novo centriole assembly	0.071913	6.425510204	PLK4, CEP152, CEP63
CRD-mediated mRNA stabilization	0.071913	6.425510204	SYNCRIP, DHX9, HNRNPU
negative regulation of double-strand break repair	0.071913	6.425510204	UBR5, TRIP12, OTUB1
negative regulation of DNA endoreduplication	0.071913	6.425510204	STAG2, SMC1A, SMC3

receptor-mediated virion attachment to host cell	0.071913	6.425510204	ACE2, CD81, GAS6
blood vessel remodeling	0.072403	2.342633929	DLL4, ACE, CHD7, MDM2, RSPO3, ATP7A, RBPJ
regulation of mitotic cell cycle	0.074443	2.141836735	RB1, RNF20, SDCBP, ASUN, FBXO5, SIRT1, TMEM8B, CKS1B
protein ubiquitination	0.074949	1.282715593	UBA7, UBA6, CUL3, MYCBP2, UBR3, UBR1, ARRB2, RCHY1, MALT1, DCAF6, PCNP, CAND2, CAND1, TRIM24, TCEB2, FEM1C, ARIH1, SOCS4, ATG3, ZER1, KLHL2, WWP1, LMO7, MARCH7, KLHL4, FBXO11, SIRT1, PJA2, FBXO30, UBAC1, MARCH2, ITCH, CDC34, KLHL8, KBTBD2, G2E3, MDM2, RLIM, BIRC6, FBXL5, TRIM33, SPSB4, NFE2L2
establishment of endothelial intestinal barrier	0.075799	3.894248609	RAB1A, RAB1B, RAPGEF2, RAPGEF6
histone exchange	0.075799	3.894248609	NASP, VPS72, ANP32E, ANP32B
rRNA catabolic process	0.075799	3.894248609	EXOSC6, EXOSC4, XRN1, DIS3
regulation of ventricular cardiac muscle cell action potential	0.075799	3.894248609	DLG1, PKP2, DSG2, TRPM4
positive regulation of telomere maintenance	0.075799	3.894248609	RAD50, ATRX, DHX36, NBN
positive regulation of transcription from RNA polymerase III promoter	0.075799	3.894248609	ICE1, BRF1, ICE2, ERBB2
nucleocytoplasmic transport	0.077561	2.570204082	CDK5, RGS14, NUP155, RSRC1, ANP32E, NSRP1
regulation of release of sequestered calcium ion into cytosol by sarcoplasmic reticulum	0.080298	2.974773243	ASPH, CHD7, FKBP1B, DMD, CLIC2
DNA-dependent DNA replication	0.080298	2.974773243	POLD3, POLD4, RFC1, REV3L, BAZ1A
transcription initiation from RNA polymerase I promoter	0.081834	2.271645022	TAF1D, TAF1B, TAF1C, POLR2E, POLR2F, GTF2H1, PTRF
mitochondrial electron transport, NADH to ubiquinone	0.082136	1.96699292	NDUFA8, NDUFA7, NDUFB7, NDUFS7, NDUFB10, NDUFA3, NDUFB1, NDUFV1, DLD
negative regulation of protein ubiquitination	0.082891	2.089596814	UFL1, GCLC, CDK5, NXN, GLMN, SPOPL, ARRB2, VPS28
endosomal transport	0.084521	1.784863946	AP5M1, ZFYVE16, SNX17, ALMS1, CHMP2B, KIAA1033, SNX33, VPS25, STAM2, VPS28, PICALM
regulation of catalytic activity	0.084521	1.784863946	GPSM2, DMPK, PPP4R2, FXYD3, PPP2R5B, PPP2R3B, SHOC2, BRCC3, COX6A1, IPO7, TTN
NIK/NF-kappaB signaling	0.084521	1.784863946	PSMD8, PSMD12, PSMB4, PSMC6, PSMD14, CHUK, PSME4, REL, PSMD1, BIRC2, PSMB9
negative regulation of transcription, DNA-templated	0.085092	1.223293526	RB1, ZNF253, LDB1, CBY1, ARID4A, RSF1, PTPRK, AHR, RBPJ, CCAR2, ING4, NIPBL, HEY1, MECOM, TRIM24, CIR1, LRRFIP1, SOX7, USP47, SFMBT1, EED, ZNF281, ZBTB33, SIRT1, AES, GCFC2, ZNF91, ZEB1, ELF2, ZNF93, THAP7, GAS6, PFDN5, CLOCK, HDAC5, HDAC2, ZNF350, STRN3, BCLAF1, BTAF1, ZNF148, BRD7, SNX6, FZD1, UBE2I, WWP1, PEX14, TBX2, CENPF, GCLC, CDK5, COPS2, RLIM, MDM2, PDCD4, RBAK, TRIM33
negative regulation of ERK1 and ERK2 cascade	0.08761	1.846410978	RPS6KA6, DLG1, RGS14, GPER1, RANBP9, PIN1, TIMP3, DMD, VRK3, EIF3A
phosphatidylinositol biosynthetic process	0.08761	1.846410978	MTMR2, PIKFYVE, PIK3CA, IMPA1, PIK3C3, PIP5K1B, PIK3CB, PIK3C2A, MTMR6, MTM1
glycogen biosynthetic process	0.088993	2.471350078	UGP2, AGL, GBE1, PGM2, ACADM, PGM1
protein targeting to membrane	0.088993	2.471350078	SDCBP, ATG3, MYO1C, RAB3IP, ATG4A, SEC63
positive regulation of canonical Wnt signaling pathway	0.092136	1.517134354	PSMD12, PSMD14, USP34, WNT7A, CCAR2, PSMB9, PSMD8, ASPM, PSMB4, PSMC6, TNKS2, UBR5, PSME4, RSPO3, PSMD1, PIN1, TRPM4
cell proliferation	0.093625	1.258182781	EHF, CTF1, USPL1, CUL5, CD81, CSE1L, PDS5B, CRIP1, CKS1B, EPS8, COL4A3BP, ERBB2, CDC27, UBR5, NBN, ASCC3, TRIM27, HRAS, EVI5, DLGAP5, APPL1, USP8, CDV3, STIL, TXNRD1, NRDC, LIG4, BST2, TPX2, FER, CENPF, ZEB1, MELK, NASP, CDK5, COPS2, HOXB4, CDK1, YME1L1, GAS6, MET, BLZF1, PICALM
positive regulation of interferon-alpha production	0.0943	3.569727891	MAVS, TBK1, IRF3, HSPD1
histone H2A monoubiquitination	0.0943	3.569727891	RYBP, PCGF1, TRIM37, DDB2
vasculature development	0.0943	3.569727891	PIK3CA, ZFAND5, TCF21, ANP32B
PERK-mediated unfolded protein response	0.0943	3.569727891	HSPA5, EIF2AK3, EIF2S1, NFE2L2
receptor catabolic process	0.0943	3.569727891	SH3GLB1, CDK5, KIF16B, CAPN1
regulation of mRNA splicing, via spliceosome	0.0943	3.569727891	YTHDC1, CWC22, SRSF10, SRPK1
negative regulation of DNA replication	0.094526	2.81820623	TSPYL2, TERF1, PDS5A, WAPL, ATR
nuclear envelope organization	0.094526	2.81820623	DMPK, NUP155, LEMD2, SYNE2, ZMPSTE24
response to toxic substance	0.095915	1.63787515	CHUK, PON1, XPA, AHR, SLC7A11, BPHL, WAPL, SCFD1, SCN9A, MDM2, DHX15, CDK1, SDC1
mitochondrial translational elongation	0.095915	1.63787515	MRPS26, MRPS11, MRPS18A, MRPL37, MRPL34, MRPL43, MRPL10, MRPL21, MRPL11, MRPL52, MRPL3, MRPL53, MRPL1
negative regulation of apoptotic process	0.09691	1.223906706	DDX3X, SYCP2, KIF14, CIB1, RPS6KA3, CASP3, CFL1, BNIP3L, USP47, ANXA1, SIRT1, TOPORS, DNAJC3, HAX1, DAD1, TAX1BP1, BIRC6, GAS6, IL6ST, BIRC2, PRKAA1, HDAC2, TMF1, PSEN2, MALT1, HSPD1, HSP90B1, MTDH, CLN3, PRDX5, PDCD10, BNIP2, HSPA9, PRELID1, STIL, HSPA5, API5, WNT7A, CFLAR, PALB2, HIPK3, HIGD2A, SETX, AHI1, ITCH, GCLC, ALMS1, WNK3, CDK1, MDM2, PDCD4, NAA15
