# Supplementary material for: Endothelial senescence mediates hypoxia-induced vascular remodeling by modulating PDGFB expression
Source: Front Med (Lausanne). 2022 Sep 20;9:908639. doi: 10.3389/fmed.2022.908639 (PMC9530050; doi:10.3389/fmed.2022.908639)
Supplement: Supplementary file 6 [file Data_Sheet_3.PDF]

ID	FPKM Hypox 3W rep1	FPKM Hypox 3W rep2	FPKM Hypox 3W rep3	FPKM Hypox 0D rep1	FPKM Hypox 0D rep2	FPKM Hypox 0D rep 3
1300017J02Rik	0.07489225	3.818185288	0.875129176	0.024292108	0.034301095	0
1700018L02Rik	10.88500097	14.03406882	14.94967152	7.179515837	6.320396265	6.770613713
2510009E07Rik	21.91886641	14.5204928	15.21123147	11.53663424	7.419670959	8.075732425
2810408A11Rik	1.967748384	2.518186982	3.135475584	1.221498301	0.74585416	0.917014804
4631405J19Rik	5.444035882	4.462106081	5.587099719	0.62851635	0.824090081	0.623510472
4732471J01Rik	18.50243491	10.68441643	11.41658656	7.878517585	5.030444231	5.083838541
4930452B06Rik	5.863065882	4.22552125	5.263326066	2.45084495	1.276472053	0.837013512
4930455H04Rik	22.99683538	17.05115261	28.34336411	7.830174125	3.957031635	5.151284503
4930578C19Rik	20.33067176	10.7219869	6.841299892	7.710617416	3.123134735	2.70210977
5031414D18Rik	1.542480894	0.578230514	0.614460063	3.898328882	2.914169808	4.535954404
8430408G22Rik	155.4161091	419.400939	483.3341401	32.77267232	152.2778498	109.9540439
9430020K01Rik	199.9198044	115.9364566	134.5815018	91.94883919	58.43059831	52.15009104
AA467197	13.39966676	15.36880992	22.62407814	2.763227237	5.364905667	8.394983964
Aard	4.423809648	3.506237417	5.236433515	8.855446339	12.38834435	11.76744961
Aass	0.276217026	0.586758412	0.048903708	0.881007333	1.707872011	1.797358927
AB124611	7.778116935	7.916603964	13.5709849	20.42293429	18.38927115	32.88624169
Abcb1a	83.21009998	47.3541688	57.78526849	29.14796664	15.47529368	14.2908383
Abcb4	0.139370036	2.915310546	0.71064589	0.149180366	0.076598765	0.251138287
Abcc9	2.23014244	2.575828494	2.329037495	0.872443728	0.766063859	0.88246556
Abcg2	35.58985348	45.79385657	48.46330026	19.58996106	26.00122158	19.00109157
Abhd6	7.232842132	7.406178723	10.04518038	2.985052219	3.828277582	4.690953331
Abi3bp	13.46175822	9.185912781	6.984742807	34.61317965	20.7676422	15.80835284
Acap1	7.230733081	3.345346195	3.500260276	16.61053601	13.55079828	16.8022088
Ace	1118.635522	1067.373455	1009.546532	504.9488108	377.4815411	342.3715618
Ache	1.617530399	16.86270401	6.382199257	0.499679428	0.211668151	0.346989779
Ackr2	92.56250283	56.83021761	70.96689583	35.13665444	28.52474291	24.80324105
Ackr3	106.0925099	158.4778612	123.0031812	46.54232682	57.97942664	46.3371258
Ackr4	2.181250098	2.119927228	2.402936701	10.71749588	6.993178471	9.09840435
Acoxl	1.548366511	0.848330793	0.885668211	5.56315585	14.78328046	20.29964998
Adam15	307.1283816	230.1385014	249.4174047	93.42647862	79.47089802	87.99292404
Adam23	10.48821837	8.19160596	9.237808842	0.859867135	0.805654267	0.63245619
Adamts4	1.747247951	3.269161784	2.710208108	0.902769585	0.254946882	0.696561749
Adap1	3.574733942	3.727325155	3.319581286	10.52767693	5.594838908	7.785260739
Add2	0.366746335	1.427197888	0.575109817	0.11329345	0.063989333	0.026224593
Adipoq	0.921968987	3.317941761	1.469095983	0	0	0.207668125
Adora1	0.80031788	1.756188456	1.617394141	0.484360162	0.335259458	0.527610511
Adora2a	41.46084619	37.72374913	55.04844819	3.289556235	3.775886686	5.403837238
Agt	1.266190795	10.21092517	13.7786664	0.240411288	0.381900448	0.695614819
Aif1	2.14191835	2.043852125	3.30957943	6.315947971	6.867079253	13.15785242
Aim1	3.997585917	2.704328341	2.050078278	8.078042948	4.73513298	6.140883117
Ajap1	0.592561222	1.166201897	1.534335915	0.25226724	0.228990846	0
Ak4	2.530503023	1.880724059	1.823250001	0.406829272	0.544219236	0.396508532
Akap12	149.5608143	66.88650852	90.88744436	55.02568726	43.50822402	24.7778956
Akr1b7	0.045507444	0.204712753	0	0.664237317	1.438144876	1.23003428
Alas2	31.28079113	276.7823999	274.9185604	2.485368612	9.897313224	8.66182806
Aldh1a3	0.701564165	1.089553522	1.117895626	0.211305612	0.48198083	0.225747468
Aldoa	519.5153709	580.7162081	669.3948733	249.5388106	302.9845449	322.8947884
Amd1	125.2074145	93.23561237	111.4973283	12.32506222	12.85167671	13.88565211
Amd2	125.5421262	93.49808359	111.8112088	12.35975899	12.88785596	13.92474216
Ank1	0.233017489	2.691366598	0.702455298	0.177719206	0.112492226	0.085112152
Ank3	1.031155655	1.482438368	0.81306935	1.856805949	2.928594441	2.753619091
Ankrd36	0.012646023	1.166192623	0.2821083	0	0	0
Ankrd37	154.7994088	210.2635482	248.6646464	5.196334406	6.474141995	11.88670296
Ankrd63	0.05841729	0.315344393	0.186168098	5.513948612	3.499614925	1.684243606
Anpep	6.172101584	5.597754505	3.701280677	11.0916571	20.24395924	18.83537156
Ap1s3	0.809640081	1.199235209	0.88104894	2.344323525	2.550517803	3.825252644
Aplnr	24.04631705	8.06444178	6.601863429	61.74618888	39.60866994	29.64782964
Apol10b	8.350272184	9.841055359	5.10351813	4.981044063	2.200382439	2.318856166
Apol11b	0.717853928	9.625579708	1.935750001	0.26866575	0.151745244	0
Aqp1	1136.997542	1349.655159	1302.49113	727.9902367	602.848583	601.3334545
Aqp11	5.627776804	8.793047624	9.857389566	1.630345874	3.364594944	1.935303558
Aqp3	0.322140043	0.362282353	0.102661727	2.852565838	1.372141087	1.451203235
Arg1	9.074621761	3.978385145	15.07250703	0.673501561	1.003835368	1.558987253
Arhgap15	2.515422196	2.027631076	1.992494337	6.013167311	5.973492694	8.056630064
Arhgap4	7.131226063	2.269408223	2.343475724	19.08715231	13.11157039	15.09968008
Arhgap9	9.909006905	7.687145764	9.722140201	27.23051074	20.10756243	26.03936868
Arhgdib	60.32780831	74.25947872	76.92826569	132.7677924	142.7698183	185.4620287
Arl5c	2.946993245	1.227489665	3.478397664	12.53276671	12.29763479	16.38995069
Arsg	3.429344437	5.041903253	5.384463957	3.011470038	1.838821597	1.657919548
Aspa	7.094542355	4.903516402	4.710283021	2.516927867	2.233922984	2.330422788
Aspn	7.754421929	14.7790243	21.78686214	1.01079346	2.32345785	2.50297065
Atoh8	8.27180396	13.66907472	15.67834205	3.707769881	6.660311378	5.649278461
Atp1a3	3.835677979	2.343215679	4.124901406	14.29996975	5.682370144	7.75078855
Atp8b1	10.45913687	9.302859535	6.632648994	5.646594789	2.556736523	2.145302024
AU015791	9.757694814	4.468298112	5.586192133	0.22742611	0.080282913	0
B3gnt5	1.375535339	0.524387347	0.891589286	2.45015223	6.823256647	4.253616083
B4galnt1	22.32706702	15.06944786	13.75742058	41.15015813	36.22968442	42.70601233
Bank1	2.063938457	0.078682327	0.278707461	6.774034126	4.854656078	7.564313061
Batf	3.455343946	4.23918444	5.005327021	8.130735012	11.04920234	9.905585798
BC051537	0.749072165	0	0.325526304	3.015032117	5.380110326	7.209121778
Bcl11b	0.465930055	0.161227764	0.182751609	1.248561594	1.566018383	1.227084558
Bcl2a1a	10.63090471	5.616741863	8.18559611	20.89777383	14.99918807	18.32071159
Bcl2a1b	29.06567569	16.0511947	24.16393686	65.82557031	46.16729864	60.27811971

Bcl2a1d	15.48259276	7.54248193	12.50577183	29.22911223	21.56745997	27.64177539
Bco2	0.32844312	0.369370866	0.174450723	1.091974137	1.654717892	1.109698397
Bdkrb2	11.59529662	7.983797362	8.726430138	4.440900917	4.226895374	3.04579917
Bean1	1.105383991	1.130115817	1.120862615	0.228163853	0.253136215	0.075448874
Bex2	1.88891207	8.355548459	5.618396344	7.658612076	25.17536866	31.20086955
Blk	2.730667256	0.361286992	0.365641667	9.690833301	8.544437455	14.47216091
Blnk	1.733319281	0.609159551	0.776792382	6.298629563	8.149577082	10.61454475
Bmp2	5.908668097	9.63608072	8.983649647	1.208815056	1.68499561	1.578417517
Bnip3	141.4980876	195.6121285	219.5411683	12.39990355	33.19597874	29.13976427
Bpgm	19.00433094	80.98072218	76.05541161	13.97647819	14.5655255	17.07297098
Bpifb1	8.525628167	2.611522502	2.537279791	76.05331041	16.86503905	14.49601147
Bpifb5	0.277548147	0.624267601	0.221127215	0.911510512	3.098518295	1.875482935
Brinp1	0.457191061	3.123059672	3.453645798	0.197726457	0.395526194	0.305124783
Btk	8.06531923	3.275404046	4.069656659	17.26608262	10.86641105	13.82684688
Btla	4.037853664	0.631793547	0.951120441	11.15540888	10.13128174	9.648639845
C920009B18Rik	0.37736405	0.16975516	0.240521761	1.13833946	1.313545815	0.849990466
Cacna1i	0.34212825	0.013042757	0.055439848	1.100325324	1.115468533	1.123282275
Calcrl	484.7695801	354.5777031	337.6414364	276.7311399	165.4124324	156.5551063
Car14	108.55382	91.42307773	112.4938778	31.52629722	30.68575974	34.63776534
Car3	3.565831131	25.73195121	7.220765292	0.216865447	0.867622535	0
Carhsp1	125.8263404	140.250295	137.9203191	67.31062487	68.85923786	67.80083657
Cass4	2.356822041	2.099753766	2.389824476	4.914389589	3.869156141	5.860147151
Ccdc88b	4.821541541	2.112269034	2.153370615	12.50389579	6.57766212	7.429317759
Ccl22	5.770273486	1.622328785	2.398577031	12.38951141	48.21709305	55.80321175
Ccl3	64.12609405	36.22016424	51.54853246	113.2573392	122.880417	135.3722791
Ccl4	19.1031974	13.35471559	22.76119376	52.08264671	90.21317958	116.6817973
Ccnyl1	18.4651003	13.53833672	16.33574462	6.102717249	5.282368656	6.122837307
Ccr3	0.416449419	0.390286458	0.165896082	1.722268122	1.025207649	0.78168998
Ccr7	12.79646271	5.691740094	10.63045292	51.0952196	72.14779469	146.6425071
Ccr9	0.336207998	0.174509232	0.412096141	1.195385189	1.190424506	0.932047834
Cd180	3.46717417	1.922152417	1.711882095	13.87553488	5.70333128	8.359579494
Cd19	8.55629609	0.367071143	1.411683036	27.90451151	24.82640329	34.13395622
Cd2	29.14131205	14.5159952	20.7255541	61.73783932	55.388473	71.11834566
Cd209a	2.832010397	1.032944133	2.073365651	11.50724011	21.61211692	13.27508695
Cd22	7.977523824	1.878115643	1.965086181	18.86319792	12.0732717	13.25242996
Cd226	1.141803168	0.667724106	0.727754825	2.133251426	1.819875154	3.394835264
Cd247	3.984411932	2.219648268	1.611793577	6.842048328	5.186513337	7.779886673
Cd28	1.341884535	0.680573878	0.964287787	3.059584479	4.519052091	4.622672264
Cd300lg	4.802068043	6.031682927	7.618650031	14.74665195	12.22510116	12.92346412
Cd34	306.8051076	270.0052645	308.3296723	157.9499064	144.2203353	142.6145202
Cd37	18.08179987	9.243180738	11.52485601	44.10766487	36.87181582	56.46409171
Cd3d	2.604805011	2.977416159	4.899048499	6.689918574	19.18604679	20.96792271
Cd3e	2.768475119	1.245383433	2.52078865	6.580945091	11.90087963	12.29349025
Cd3g	3.830632435	3.746063278	6.546165326	11.34462795	23.95208928	26.25996939
Cd4	1.724901202	0.742918785	0.99414366	4.874707824	4.361891287	6.117185022
Cd48	19.24606223	9.507849748	15.2147916	39.00457361	29.58769818	36.96565368
Cd5	1.399931256	0.987845396	1.574609595	3.846348208	5.280279754	7.172128351
Cd52	59.50310856	60.69590679	90.67622569	154.4770376	223.4003733	260.424912
Cd6	1.179293752	0.715752433	1.014131386	3.406219468	4.39814883	4.637967095
Cd69	9.290260332	4.652286858	7.485497458	25.2101843	57.41833944	40.90395479
Cd7	2.512010885	2.456553035	3.132564665	8.183403741	10.65477769	12.3003428
Cd72	5.138980294	5.014440925	5.900624504	11.91331503	16.51039741	17.70332771
Cd79a	11.46993339	1.347679434	3.546200642	43.85347823	70.15504512	111.0534643
Cd79b	10.1924518	1.919310217	1.510789859	36.76614538	42.86062138	80.72639003
Cd83	28.6751733	19.5698125	36.85790964	80.27684921	110.8745652	147.2226364
Cd86	2.594730835	1.76090056	2.637545147	3.678498646	5.747358572	8.061351093
Cd8a	0.943530689	1.387599286	1.329977756	2.56017859	5.459956275	5.558340211
Cd8b1	2.165845789	3.608495983	4.218048466	5.776237727	15.37553858	17.52625116
Cd96	1.275961114	0.468558488	1.244791094	3.471454668	3.899960603	3.519217749
Cdh16	0.3594512	0.461991891	0.872779386	1.016014837	4.068732228	4.240986187
Cdh4	2.641340127	2.215274059	1.626451148	1.115223353	0.676639529	0.645365613
Cdk19	293.7465907	177.8955585	185.5743223	19.57623016	12.02709099	13.92245448
Cebpa	17.36725452	18.00665476	16.94019644	29.86253915	42.34411059	43.33598607
Cecr6	0.023328523	0	0	0.181604583	0.30451116	0.157638404
Cenpa	4.927465663	4.490512802	4.061165858	9.879002389	8.1712033	15.30872881
Ces2b	0.419581031	0.269637483	0.28653186	1.837290933	2.347226658	2.160186852
Cfd	7.290616461	42.71805277	18.15783617	0.298082766	0.336720567	0
Cgn	3.348076023	6.896682438	4.598462884	8.534302463	13.15039853	11.42628113
Chil1	39.24083418	69.20236435	55.18721251	90.14435806	346.4217681	362.4879709
Chrm1	0.072279083	0	0.057585945	0.395625917	0.372422804	0.366309923
Chst10	0.236714577	0.040955677	0	0.726465641	1.100846305	1.476514378
Chst2	2.001685951	2.476230646	3.479512177	6.507439985	5.78824112	6.55808088
Ciita	44.59265842	20.05976418	14.81513495	14.02727335	5.592530571	6.291691243
Clca1	3.025717942	14.83686499	4.072633523	1.883602948	1.988317123	0.677292205
Clcf1	8.67868117	6.402654119	6.19534658	18.30891397	13.73721122	14.38749144
Cldn13	0.106155681	7.282417033	2.029823833	0	0.072929899	0
Clec12a	4.881871426	3.449345395	6.679295796	13.92805808	11.20304334	12.66569952
Clec2i	1.675944954	0.530925843	0.52657871	4.938436043	3.146049118	3.402788101
Clec4a1	4.009795798	4.181501058	5.808492461	13.30179992	8.464642854	14.28671395
Clmp	3.722878661	10.51059154	7.476991341	3.056625554	2.770788364	3.406636423
Cnr2	4.958377872	1.919420713	1.68801412	10.60907452	8.652584551	7.158417118
Col12a1	1.59441866	1.787651189	1.776103071	5.432609911	4.308271995	3.62408257
Col15a1	10.16492041	9.966612988	9.90202179	4.571611146	3.609063341	3.367042512
Col4a1	514.2303975	357.9830942	353.25711	232.2029866	191.1768817	201.7382663

Col4a2	360.3183353	309.2336178	294.6129565	163.7725997	147.4237157	151.6529286
Col8a1	3.000819606	6.449533368	5.489991564	2.000771189	1.481290446	1.552105879
Coro1a	68.92009718	67.42984272	96.33295637	189.8096606	188.1732951	284.5237968
Cr2	0.722840322	0.020580112	0.058318874	2.234799538	1.571511832	1.813641652
Crmp1	1.719005272	0.78072058	1.158858484	0.546855216	0.431508975	0.223382244
Cry1	5.799845222	15.12050747	12.00216347	4.580947011	3.156587853	5.344286873
Csf1r	25.3847684	23.47133408	23.12034824	68.04313367	43.67945719	58.30013318
Cspg4	6.137908315	7.331208708	5.05880008	3.748377817	2.278040125	2.256539907
Cthrc1	14.24711388	15.03477991	22.70182411	2.468862824	3.419058907	4.835598683
Ctla4	1.43966435	1.58602079	1.498128299	3.202084331	5.73252395	7.279664856
Ctsw	2.489316264	2.799513361	3.085101564	5.607198127	8.487572129	12.46008752
Cx3cl1	13.04637451	12.43673938	11.517189	41.61984646	38.13609512	33.13069744
Cx3cr1	2.451508903	1.429552845	1.205652978	10.17527323	5.115003441	8.316906448
Cxcl12	518.2774306	284.6651324	333.4753101	68.9938598	34.42431758	33.76281778
Cxcl15	53.08183342	128.0410417	88.92209366	108.7363045	461.532766	444.2141289
Cxcr1	8.539204572	5.455121329	4.669738548	2.556722711	1.110818385	0.910488008
Cxcr3	0.600426569	0.317763079	0.675345616	2.440163356	1.89263972	2.386633678
Cxcr4	57.20029772	36.97017522	56.47289184	148.9461738	133.7435439	176.300393
Cxcr5	3.170868175	0.53734162	1.107411855	10.39776521	11.39937543	22.60064516
Cybrd1	1.221361066	0.760361654	1.042584246	3.5654545	2.18761689	1.522899585
Cyfip2	4.468158932	3.107649678	3.038430753	11.95865309	7.160701313	9.354490325
Cyp1a1	0.489070309	1.787545588	0.714359123	22.16927199	15.73579664	15.88143292
Cyp4b1	103.2053365	135.387452	120.3392975	411.7223352	504.9126709	470.8079475
Cyp4f18	14.04816387	8.856292256	14.24971314	29.41814191	25.3086459	27.20818483
Cytip	20.91498301	10.11234595	16.4423989	38.42981769	41.20138698	53.17694496
D330041H03Rik	20.66602181	17.12965687	14.63552224	26.06818148	55.31860584	43.96295029
Dapl1	1.090272575	1.783466077	0.947605367	3.954356605	6.400775918	6.697568856
Dcst1	1.473874297	0.939270278	0.626272059	0.358550247	0.101256477	0
Dennd2d	2.295325996	2.480403064	1.7980753	5.053542701	5.356206236	5.14120447
Dgkg	0.784067213	0.672930423	0.558923638	2.158379132	1.219075253	1.4407377
Dhh	7.222129346	4.7491283	6.881848863	3.87575599	2.406655843	2.486051541
Dhrs3	38.36786508	23.81366381	19.33811019	67.87359502	65.87594999	66.77368064
Dmpk	16.97625504	24.02081641	25.70619893	10.07176305	10.40466554	11.77267592
Dntt	0.106654065	0.059972187	0	0.622699096	0.696086779	1.081044213
Dock2	11.18870293	4.579235395	5.13414242	28.8984644	15.33698945	19.14068307
Dock8	10.52966496	5.920890438	5.260605108	23.77578365	12.86927901	13.9225391
Dok2	3.406935834	3.34789406	3.794836636	7.209818301	10.58948924	10.13255962
Dok3	17.74331104	12.3113667	11.82422712	35.60380761	23.26916629	31.93951886
Dok7	0.503202727	0.926030463	0.947605367	0.178057333	0.25142164	0.103039521
Dusp2	8.424220951	5.684384772	7.606612179	25.44628296	29.13049127	38.89888384
E130006D01Rik	1.159046726	1.097664943	0.972033432	0.17808124	0	0
E330020D12Rik	0.341523142	0.406673796	0.06402286	1.133832188	1.435380106	1.357519262
Ear6	3.031102534	24.57933121	14.74378126	1.086662397	3.178391508	2.874686857
Ear7	4.490067766	34.66469282	20.88376443	1.535126243	4.835501611	3.826773317
Ebi3	2.085492273	0.763608287	1.39106202	4.058711228	4.131656254	4.806692457
Edn1	147.2409645	118.8032645	147.7128378	32.6306015	64.48540041	83.82704611
Ednrb	21.30889441	11.29281096	14.50434585	53.93210451	38.59641142	36.59994539
Egfl6	2.60828441	6.245103715	3.083578057	8.59670696	21.01164401	17.81454093
Ehf	1.087448444	1.776199276	0.41256582	2.960375659	4.695973959	2.857649735
Eif4h	268.8310009	293.7135979	340.7229368	116.3790102	155.4221209	152.0851352
Eln	144.0936331	209.6016031	240.3980331	61.15494716	65.42473502	65.83952864
Eltd1	244.6426967	157.9181896	158.1150868	101.5650888	89.22900272	68.47929767
Emr4	1.675654204	0.87819484	1.29613739	12.47449477	4.828289363	10.04040586
Eogt	52.91256246	41.25169558	36.48088481	26.26143577	16.21569558	12.77239964
Eomes	0.152875611	0.20631078	0.389755316	0.595042205	1.470376691	0.96416146
Epb4.2	0.147217629	6.401754567	2.189426916	0.015917208	0	0
Epcam	6.916601496	13.94549758	8.167061685	15.98140159	28.35915024	23.58610129
Epha7	0.135137445	0.207968673	0.181332624	0.394499568	0.762270424	0.736942266
Ephb1	3.748089391	4.516225635	4.964014573	0.94732453	0.501617687	0.219281877
Epsti1	2.568172009	3.245781529	2.961980945	9.615364374	10.11564705	13.99336156
Ermap	0.311982474	5.585675852	0.954476599	0.133577234	0.18861457	0.168653349
Etv4	0.437619526	0.76556961	0.852276916	0.141946605	0.100216171	0
Etv5	6.122144245	9.258029804	6.345633638	11.6544278	22.70405823	25.5713249
Eva1a	6.103351256	5.346615428	7.063626206	2.62569104	2.868933512	3.907167714
Evi2a	6.381824351	3.44944555	5.045090595	13.79206626	13.42500943	15.71186645
Evi2a-evi2b	9.756869375	4.447079863	4.520249719	20.04803616	12.69741915	12.32764932
Faim3	4.83752366	0.514047315	0.849730634	20.12652434	29.04723024	51.82148448
Fam107a	48.98963147	56.71949678	55.49519421	3.339778467	22.58646383	15.94975749
Fam107b	21.41475055	17.97622481	19.72060686	45.81971467	49.62708164	79.89127354
Fam124a	12.27833559	10.8814871	12.16115755	7.193557553	5.067777071	5.61036721
Fam129c	1.350041936	0.514668645	0.218766267	4.186033204	2.703871584	4.020160388
Fam13c	18.12207531	28.35044169	17.583614	8.76990069	4.802153324	4.810800741
Fam169b	1.207939516	1.26789834	1.026544488	4.13357637	3.37478517	5.078851222
Fam181b	15.539532	10.9571334	18.07961076	3.360276336	2.330035034	3.750202236
Fam188b	4.723872362	5.659569624	5.635925003	2.26370967	2.772403042	3.368520211
Fam198b	34.08282973	35.42892905	39.51959084	11.85014608	11.29252405	11.00293227
Fam46a	2.290301596	1.972382273	1.052091554	5.00944738	3.869855165	2.92795081
Fam65b	3.892062863	1.872167437	2.824555827	9.411996696	7.899864794	11.31851872
Fasl	0.117462853	0.462350209	0.467923022	0.857257674	1.533262501	2.513493007
Fat2	0.027563824	0.00885674	0	0.153267822	0.081156824	0.106432974
Fbln1	6.002267877	4.867763322	5.98100071	12.306603	25.94971287	24.06897683
Fbln2	30.05211146	18.00411395	19.00078932	10.41842211	8.192192542	8.514903377
Fcer1a	0.055354083	0.124503663	0	0.861825259	1.292977442	1.994909398
Fcer2a	3.757316868	0.162520048	0.537297869	15.67938102	11.74827056	19.31331179

Fcho1	2.055185236	1.728765772	0.958478156	4.796422683	3.673315282	3.76356473
Fcrl1	1.446254191	0.062556688	0.44317489	5.602233478	5.655817211	8.895762122
Fcrla	2.236215224	0.295867418	0.733612262	10.68810535	16.76374166	27.40690163
Fgd2	4.175659005	1.459894375	2.34428543	11.78976955	9.542564677	9.649091772
Fgd3	2.976625788	0.739335576	1.687712581	7.019130281	5.244152163	5.34728729
Fgfr2	4.961179617	8.721544444	6.060172071	12.00189608	18.17313214	18.42520486
Fhl5	0.770949632	0.80921747	1.310353847	0.050013163	0.070619902	0
Fibin	18.28145522	14.01505656	15.57113933	46.02262474	58.00764473	53.04901832
Flt1	128.1285552	128.1475755	98.01845827	90.41209119	42.44705908	40.90294886
Fmnl1	28.40397674	14.18242341	19.01988082	61.5001776	36.38812153	42.01362636
Foxs1	2.166029351	4.287256555	7.455073802	1.728334351	1.488081455	1.170925082
Frem2	0.547327621	0.144830791	0.249179999	0.102938494	0.056876816	0.082878889
Fst	3.475156358	16.39804336	14.71484757	1.016848705	1.602772611	0.875813882
Fyb	5.557718352	3.578888551	4.708633432	18.22612963	10.74406127	11.26403215
Fzd6	40.19998675	22.65870378	25.19375352	14.42380578	8.259866187	8.717845668
Galnt12	1.332694521	1.037605433	1.06177984	2.543765146	2.852362054	3.579088917
Gapt	0.746189206	0.745931432	1.321114052	2.58170136	2.696099464	2.489996402
Garnl3	9.758988643	7.35442804	6.252181026	5.058166186	3.432894519	2.492753263
Gata1	0.298597737	5.91019279	1.237068415	0.261504155	0.369250748	0.40354437
Gfi1	0.255907967	0.287797008	0.233012713	0.782632755	2.210196091	1.646907823
Ggn	1.434282096	1.107439664	1.091549944	0.520774074	0.470621886	0.385747652
Gimap3	11.43808909	5.65751608	6.075276926	24.34731692	19.17223335	30.53466917
Gjc1	25.36740877	23.8689716	28.02707546	13.00789697	9.452277018	9.244304433
Gkn3	0.143236544	0.32217089	0.228237863	1.115047561	2.558524312	3.226319424
Glb1l2	16.27067656	37.28948871	34.37190384	13.49378466	12.17311995	9.995113067
Gm11651	650.0387215	634.7986009	590.5921861	312.3358221	203.2174316	195.9504972
Gm11978	3.2179965	3.618995435	3.880393187	0.846317684	0.896267131	1.371309274
Gm12250	23.58329789	22.45627681	8.841978724	5.788255427	1.328500966	1.230944742
Gm15987	1.049783541	0.708359101	0.501827981	3.983950545	2.163631927	3.546864099
Gm16897	51.16183935	45.02464285	49.77115544	28.4864213	21.89741432	21.67151525
Gm5483	3.162822422	12.68128588	23.66489529	1.605749484	1.700520396	3.097422885
Gm694	0.753060193	4.375650267	2.399902211	0.061065795	0	0
Gm8369	0.702682622	0	0.263453603	1.64908758	1.930996879	3.724121256
Gp1bb	1.716056232	15.37790418	15.45164857	1.245771512	2.245611267	2.33146066
Gpr128	0.693883212	1.276934639	1.206170771	0.061382316	0	0.094723114
Gpr132	8.732055593	4.24231299	7.643394992	22.25083351	24.44392518	30.63032474
Gpr171	3.369309764	1.435289413	2.359005001	7.699779268	14.37948158	23.11248233
Gpr174	0.485232415	0.071177985	0.16808379	0.903283792	1.695782691	1.615676529
Gpr176	0.551814115	1.371800518	1.712280012	0.155435948	0.279337735	0.261669272
Gpr18	1.907560871	0.975120289	1.519785402	6.20144128	11.3776209	14.25713016
Gpr183	2.548169349	1.563109205	2.276249874	6.424362441	9.946676349	10.17474991
Gpr65	2.486525021	1.30894116	1.938905625	5.328253732	6.215176315	5.719917212
Gpr97	16.02698246	18.02412661	9.205531383	8.636501865	6.625612487	6.611308125
Gprasp2	0.089250848	0.066915012	0.047405088	0.434242101	0.47009031	0.402064636
Gprc5b	11.52182096	20.83374127	16.95930896	4.237966194	6.035855697	6.276029053
Gpx3	646.5150413	2036.189363	2387.145974	383.7916802	663.8889918	732.1241877
Gramd3	5.188791908	4.854639164	4.933004369	10.96804976	14.49863182	21.70526518
Grap2	3.991699178	1.591077054	1.964510699	9.656545007	5.942878357	6.009220854
Grb14	8.501643145	10.73178464	12.34878399	16.88326214	27.41555201	27.48663162
Gria2	1.275265503	0.280241693	0.443781117	0.085581951	0.040281322	0.09905038
Gxylt2	3.545692778	0.839479241	1.387676206	0.544776454	0.384619345	0.420340302
Gypa	0.808224154	25.38034761	8.618696433	0.090746379	0.213560458	0.280073487
Gzma	2.781448123	4.946680826	8.864103507	14.28821373	30.12968351	41.37846527
Gzmb	2.205954044	4.14977038	4.090228816	6.244581327	13.88758324	10.29893109
H2-DMb2	11.77774326	1.950240577	2.9935167	32.13218441	50.53411028	83.32934524
H2-Eb2	0.672109936	0.226758742	0.107096228	1.438840218	2.401076662	4.617359452
H2-Oa	2.544599247	0.608869198	1.207767756	10.22051638	19.49015041	28.29196103
H2-Ob	12.2733064	2.200777101	3.118225002	21.67115294	22.82596568	31.42990964
H2-T24	51.09313815	53.97661718	34.09164478	32.74842179	14.55556441	15.1257011
Haao	2.551986051	0.939270278	2.070177084	8.939852826	8.543046457	8.98809363
Hapln1	2.157133847	0.859186106	1.396382271	0.218652996	0.262432217	0.404901492
Hba-a1	279.4961353	3299.664888	3539.717127	31.78452797	177.4283975	140.9676496
Hba-a2	277.5103723	3276.221443	3514.56816	31.55870539	176.167805	139.9661033
Hbb-b1	920.393311	9358.680653	10187.7909	95.64006792	514.9567427	401.4083344
Hbb-b2	163.1680224	1897.457161	1743.849578	14.82493343	87.82033172	64.16483586
Hbb-bs	908.87039	9241.514072	10060.24422	94.44269618	508.50971	396.382878
Hbb-bt	163.1680224	1897.457161	1743.849578	14.82493343	87.82033172	64.16483586
Hbq1b	0.303707431	6.831056567	3.387562502	0	0	0
Hc	9.882532768	8.487913789	8.139352634	28.86984111	72.06204888	71.75741834
Hck	14.93131418	19.35651062	23.01340262	40.23322258	35.77225471	48.67454965
Hcls1	27.04576377	18.35719512	20.18004987	57.92096736	46.40336463	58.06639528
Hemgn	0.098343359	2.986147585	0.626814286	0.047848091	0.135125526	0
Hepacam2	1.396972686	0.685549684	0.566613764	4.967877233	5.025509293	3.89034098
Hes2	0.1126404	0.050670669	0.071793981	0.438433516	0.711941612	0.9133773
Hey2	2.534613634	3.531537538	4.217442686	1.113148413	1.849170415	1.313591111
Hfe2	0.084304735	2.022614671	3.492683018	0	0.038612069	0
Hif3a	1.189430627	1.728931755	0.722010757	0.212586224	0.611472365	0.109351958
Higd1b	54.00778764	61.60794041	77.67394674	20.47950432	24.66501094	18.12540978
Hmha1	29.33572435	14.62776693	16.92897456	71.19469979	53.15315315	57.45826471
Hsh2d	0.834513643	0.417112679	0.443247245	1.894784391	3.758419999	1.670838402
Htr2a	1.758661273	2.321777489	0.852876725	0.669647799	0.499045722	0.4305741
Ifi30	66.92461035	55.34130689	66.23481387	127.992911	147.319882	145.2669231
Igf2	6.623202326	6.423225073	4.999088087	2.648418929	2.339575997	2.295134786
Igfbp6	64.81220246	77.10031774	57.56941527	126.0923399	158.3099324	158.9864634

Igtp	104.7433334	89.15963915	48.75975318	47.94329018	18.74691343	19.34978297
Ikzf1	8.327042458	5.344180454	5.118133501	19.36346338	13.25521742	15.21306779
Ikzf3	2.882626704	0.966316128	1.192484353	9.210757457	7.064661262	6.867541321
Il10ra	12.58459952	5.558716056	8.445977066	38.90510035	23.9935817	28.27282917
Il12b	0.261988522	0	0.185538314	1.10472437	2.7998203	3.147273771
Il16	6.226885027	4.224739434	4.861297206	13.28165748	9.150224501	10.87183637
Il21r	2.200277042	1.273977638	1.18023576	6.253563751	7.183888747	10.59896052
Il27ra	3.696478216	1.105356444	1.634244922	6.590993485	6.165292783	7.604184836
Il2rb	2.907074912	3.743145351	4.967898463	8.404474535	11.83839448	20.11854289
Il33	5.694601836	5.839857925	4.450068385	8.895829523	19.18647506	18.18352636
Il4	0.202111351	0.454593444	1.073503114	1.573367822	2.12906857	7.283904066
Il4i1	1.636934091	0.624038875	0.618929348	2.45680194	3.316582443	4.124550709
Il5ra	0.207799821	0.071905859	0	0.871042642	0.658893821	0.504061332
Il7r	6.33395566	1.392812001	3.044735749	10.76023067	13.00583192	12.274286
Inha	4.61954987	2.619427706	2.103126881	0.982145019	0.960102425	0.349756843
Ip6k3	8.648349085	10.6404449	9.893740027	2.637353545	3.046919408	2.996907817
Ipcef1	0.951149753	0.449573055	0.483232737	2.293746881	1.325837234	1.800865723
Iqck	4.653349492	6.87252843	7.95081127	2.073102369	3.466509988	3.283329412
Irf4	2.571039374	1.036546828	1.159465888	7.470095414	9.814778988	8.959839712
Irf5	10.23323905	8.384322314	10.06355315	29.48058491	17.81233502	24.60068561
Irf8	15.62970615	13.78172307	15.74962355	34.93376069	31.96481081	31.22294303
Irs2	7.615849898	8.819588711	7.03759958	14.0640828	22.50410897	15.62376544
Itgal	20.3151499	8.361213238	9.422008426	59.30493825	28.71986386	34.12927269
Itgb7	11.93636079	7.88228311	11.16820611	28.60363872	30.1459036	34.63599894
Itih4	3.141221769	4.0776917	4.461891516	5.467067795	15.83390157	20.78151469
Itk	2.516850983	1.695552769	1.704918755	5.193991681	6.097788889	8.928084402
Jag2	62.80323561	36.67741541	38.35043536	18.75777345	13.62821263	11.61220325
Jakmip1	1.263662614	0.321765133	0.987785108	3.990554868	4.226076367	5.155609679
Jam2	73.9008385	54.36341156	54.03101409	23.66841516	17.9009063	14.71240504
Kcnab3	2.507232335	2.970715298	1.783530007	1.154521431	0.399863969	0.605077959
Kcnh6	1.274017011	0.850709568	0.697833465	0.193706781	0.054703815	0.089676527
Kcnj8	5.788347155	6.538832209	9.347425336	2.424769788	4.458123373	3.157162943
Kcnt1	0.251297741	0.314013662	0.148305986	0.027170376	0.012788429	0
Kctd11	17.69313356	14.92342431	15.3597709	8.080561649	8.657813166	8.459309966
Kctd14	0.266530886	0.699401881	0.566265546	1.534525881	2.07523638	1.801035724
Kel	0.383738443	9.900416443	1.942289697	0	0.062030995	0
Kif21b	4.390752182	1.400819803	1.389350121	11.70258097	5.793364322	6.929952976
Kif26b	0.27606784	0.172482795	0.21994783	3.029625326	1.348700907	1.278199274
Klhl14	0.070673581	0	0.090090903	0.632695136	0.271898925	1.018803904
Klhl2	27.97741141	32.13725	35.06425367	14.53629032	14.10695839	11.48575547
Klhl4	2.865984747	1.743115387	2.423176237	1.422877053	0.582650555	0.526976581
Klk1	0.064832522	0	0.206612586	1.009398078	1.425296649	3.796818144
Klk14	6.305396717	9.354244469	12.39870739	0.174061558	0.368669251	0.201454433
Klra12	0.660387088	0.445607295	0.420913082	2.827488336	3.811011214	4.462449947
Klra13-ps	0.696850179	0.626948505	0.888307362	2.983607324	3.734189778	2.825305734
Klra18	0.415560655	0.207708549	0.147148476	1.033402056	2.157064806	2.704075361
Klra21	0.649351596	0.224697903	0.636737467	2.478884593	3.01982377	4.725408739
Klra23	0.607414862	0.546484525	0.7743	2.718897388	3.338395359	2.46269965
Klra4	0.526405362	0.263111757	0.186398172	1.195216725	2.491333406	2.898371198
Klra8	0.626751073	0.216877348	0.614575977	2.392607625	2.914719547	4.560942051
Klrb1b	2.38778297	1.790218577	2.536515089	5.196666052	9.383954676	11.68198922
Klrd1	1.967434974	1.475066487	1.880985711	5.615796695	7.479104268	7.681322619
Klri1	0.067772422	0	0	0.527585153	1.396807724	1.221227352
Klri2	0.07257001	0.228516561	0.370033478	0.706165918	0.877469876	1.373061522
Klrk1	0.798231044	0.534799823	1.082491777	2.759921941	2.613612156	3.442918919
Kmo	1.386865758	0.051137213	0.507185098	4.97779126	4.810804121	4.91619786
Krt15	0.132077418	0.445607295	0.420913082	4.594668546	1.542552158	0.446244995
Krt23	0.885665335	3.237095227	1.646456629	3.770485509	11.1043945	10.97200169
Krt5	0	0.058329113	0	1.589801972	0.28505933	0.116825174
Krt80	466.7038193	416.6336103	433.9129845	155.5444599	104.0759808	103.4060837
Lama1	2.362906354	5.073125283	1.197996993	0.865141539	0.188569532	0.349444494
Lamc3	9.519819535	12.36478836	15.84066989	3.056791008	2.947696138	2.804395189
Lamp3	12.45273146	28.81140634	17.86645716	32.765279	126.284566	111.6604736
Lat	5.522848257	5.175881596	6.893560275	9.539179927	23.58756226	25.91644351
Lck	3.26227773	4.224672582	3.780524806	10.12461851	15.58662363	17.25687781
Lcp2	11.99308714	7.058554418	6.973730178	24.11633807	15.10354674	18.72238558
Ldha	287.7624761	321.6684367	406.2487316	112.3330617	154.290947	177.104542
Lef1	2.048686605	1.316557851	1.865397275	3.807213009	4.571618705	7.286126575
Lgi3	4.802701948	13.41257878	8.364010025	13.32533977	55.14701695	53.00322505
Limd2	15.61334164	22.30050517	22.02588217	30.91067757	49.67786917	59.49529878
Limk1	16.83460434	18.33654573	19.49905501	6.85193874	7.632071093	8.2382314
Lipe	65.71497382	38.84537103	43.47805725	25.31832291	19.67569315	19.23545051
Lox	24.126213	48.75067834	52.5296796	6.014807565	13.15871766	12.80791269
Loxl2	14.16543053	15.19079282	15.72329755	6.913402509	4.956276488	4.544003087
Lrmp	3.871216065	2.971927834	3.619852502	13.15070595	8.727178643	10.96487701
Lrrc32	78.76050536	53.39269096	56.25190078	35.07618642	37.74963164	24.60584298
Lrrc3b	7.830020056	3.076237394	5.557269043	0.365990363	0.281884376	1.078223909
Lrrc8c	57.13457128	45.69588084	42.80101315	30.61136148	20.43970615	20.891017
Lrrn3	0.855126235	1.08869964	1.542550782	4.129140701	2.37208639	2.83466991
Lrrn4cl	7.013960269	4.549806317	6.516575352	4.086538152	2.386668343	1.584806072
Lsp1	69.21545959	48.67481677	65.21568494	148.3617633	126.3052932	151.7803774
Lst1	8.600906835	9.21207388	15.66282333	30.02352671	31.88929942	46.74130265
Ltb	6.223922145	3.849721468	5.950442469	18.32057511	26.79557699	28.03804168
Ltbp1	31.24084603	30.61256946	25.24599109	16.11005286	14.49070569	14.11765534

Lxn	79.36498159	81.65114372	127.1257724	41.60023423	42.51906589	43.81264961
Ly6a	1443.86453	2454.101385	1899.700038	769.6773672	893.9757162	893.166928
Ly6c1	569.4865116	1126.836557	841.9182779	214.0031307	268.1177946	282.4427895
Ly6d	7.068339947	1.478908918	1.047714183	24.63311112	40.31619677	66.64614104
Ly86	8.259806408	9.096884457	17.42757473	27.32740277	34.203903	45.93443965
Ly9	4.844197992	1.626956776	2.584611011	10.43008969	8.463077042	9.380732296
Lyz1	21.28064764	70.78756618	56.0862522	54.69660871	265.5657221	315.1065842
Lyz2	2648.598313	2700.321185	3193.997574	4901.342935	7543.628603	8019.851437
Maf	13.03657059	8.2073963	6.361080728	4.005996654	4.649531345	4.70668801
Mal	29.66416961	43.77581045	36.22458587	3.322238651	7.303490926	7.644100726
Map4k1	4.483680756	1.60977975	2.549191903	12.94558646	10.78838096	14.03460908
Mark1	2.973405522	3.49783017	4.638803316	0.714262462	1.111122552	0.952771351
Mark4	37.86219305	47.21714598	35.81394574	22.38470936	16.47750817	17.10177808
Mcpt4	1.262073102	9.290236931	16.81951668	0.390759407	2.049403818	1.292157224
Mcpt8	0.881041485	0.914611631	0.863926611	4.088784933	3.631700082	5.495523086
Mctp1	39.93671284	37.82484551	37.852736	7.640912808	4.857855615	4.652850586
Met	2.12590636	1.97794619	1.496481416	5.067855125	5.185127217	3.73078347
Mgl2	5.202998142	6.18100441	7.006166133	9.769345329	32.06986425	32.84747028
Mgll	125.4655559	129.9488954	128.8223222	44.02773908	32.5089593	32.34022715
Mir147	67.57682557	63.06189309	109.9702026	14.69057519	24.69461757	42.10142651
Mir8119	3.962322529	6.931669028	7.015218027	15.85107097	26.61658625	21.81642197
Mmel1	0.058011532	1.652757506	0.061625	0	0.026569626	0
Mmp14	46.31813143	67.23974428	66.41739381	29.15380296	31.79092069	31.32822004
Mmrn1	62.41626626	34.27728704	28.10929467	23.8533982	12.54013561	10.59591221
Mn1	2.075994919	1.751015609	1.742585653	0.964965125	0.942328553	0.542756641
Mob3b	1.836598917	1.207499701	1.140583707	3.610983521	2.911753415	3.012462065
Ms4a1	4.389952865	0.186301543	0.527931818	12.22429162	13.46739037	16.41799767
Ms4a4b	6.365543585	6.803363094	7.481412166	14.27740623	21.33866223	36.20094531
Ms4a4c	2.538063434	1.631048562	3.571531778	10.07142603	9.012747134	10.09727502
Ms4a6b	7.642013476	7.647786922	6.329913978	22.43996037	19.10400207	22.4453914
Ms4a6c	7.721894636	6.909953273	8.820303367	22.70370917	15.32558557	21.32059419
Msx1	9.293982137	8.46753858	13.30965964	3.319879436	1.939761571	2.782387228
Msx1os	7.371706691	6.013390335	8.768381289	2.601575918	1.604924414	2.338639217
Mtcl1	1.865017341	3.475222595	2.710661739	4.836976505	7.323151856	7.558001239
Mtmr11	11.45156301	12.03815818	9.332834509	4.932930559	4.745371106	3.730345783
Mx1	0.897238524	0.687009118	0.121675714	3.752416147	1.967268694	1.977977814
Mxi1	16.91973069	15.70899869	18.29574854	8.07478043	7.527133903	7.968187899
Myb	1.50366136	1.555751882	0.958393567	2.531312216	3.594940038	3.251432951
Mybph	0.714185708	2.095255732	2.37497157	0.241725729	0.255992318	0.419650843
Mycn	8.816523767	6.396879243	20.00958529	1.979816125	4.719373524	2.365304749
Myo1g	16.93322755	9.116062546	8.373713831	50.45166634	29.92088577	31.25559137
Mzb1	1.943258253	0	1.198626656	4.452882744	9.474447093	14.1195988
Nccrp1	0.185477758	0.083436158	0.118218566	0.758037518	0.81551918	1.336888989
Ncoa7	14.46962792	11.74985239	10.64662501	5.87017551	4.444105924	3.834351897
Ncr1	0.388197468	0.174628514	0.247426692	1.850965613	1.386814613	2.448297898
Nde1	44.30347901	86.07924924	65.93839205	26.60036179	28.65600311	23.6738216
Ndrg1	62.98356162	51.58251362	62.37669709	22.08669522	28.24380662	24.78795726
Ndrg4	1.45525624	2.110349674	2.440898518	0.819838155	1.052393682	0.60381993
Ndufa4l2	45.24610622	75.13382746	124.6671194	21.26882376	27.92746066	24.15154446
Neurl3	20.44822273	11.67124229	14.64477687	45.12308357	43.71530496	42.78976396
Nfatc2	0.637745696	0.610396645	0.389184954	1.861737142	1.519496311	1.222540343
Nfkbid	25.7406402	10.99460254	16.92866344	55.59446232	33.03869541	42.13203987
Nkd2	11.36923395	15.10528514	14.15584777	6.106200475	7.193169609	5.399629069
Nkg7	2.165549787	5.970662726	5.788202033	8.497008724	16.7012159	23.28743868
Nlgn2	44.46271931	28.62802669	20.6249093	13.16979955	5.912687282	4.756853882
Nnat	2.465576943	1.529829435	1.49020874	3.019694436	6.016170753	8.426103094
Nos2	9.47978217	6.371030276	5.035133179	3.199526275	2.210013544	2.372517249
Nos3	42.35735277	32.90339189	29.19235889	17.18388221	10.76932092	11.29376127
Notch3	29.1009302	30.54006031	30.98954679	18.51428347	9.952089438	8.857857338
Nppb	5.453901048	3.761891715	7.41583099	0	0.099916763	0
Npr3	8.272672295	7.143168398	5.554192669	23.85601256	22.54202292	16.85408674
Nrip3	0.116112015	2.25252039	0.416287663	0.056493273	0.159539978	0
Nrn1	1.680689211	0.945061093	1.450619067	2.18059856	7.793877081	8.044515195
Nrros	14.76082969	9.296110219	15.05305339	29.67421888	27.25414286	27.17342379
Ntrk2	118.6925941	62.04577094	69.76925941	2.905552043	0.828326119	1.239410477
Ntsr2	0.367118873	1.238598169	0.584979396	0.035723688	0.050442787	0.16538276
Nuak2	6.092766698	5.383710099	5.200937503	14.80426342	12.45769341	12.66165556
Nxpe4	43.38699462	35.46788768	35.53684382	21.40564938	9.419288292	11.2079266
Oas2	13.24294873	14.41381222	3.513055611	5.363406905	3.170676415	2.780947069
Olfr56	6.944671679	10.92084199	5.90804942	4.925130412	2.102497181	1.723322639
P2ry10	5.122847132	2.575600705	4.417577337	12.92177718	14.79545647	35.65750596
P2ry2	35.76084134	28.18812722	30.04477577	9.118649883	7.413324194	5.28750736
P4ha2	21.66092188	33.62459367	26.17598119	9.666579925	16.41264967	15.71757115
Pacsin1	0.218435729	0.115602496	0.327588462	0.962753381	0.97102365	0.868259492
Padi2	1.945629719	0.912817521	0.836872163	5.726233829	3.608175312	3.065003453
Pak1	2.950059798	2.921219551	1.95124359	7.041219356	7.24009722	7.271708702
Palmd	116.1724056	81.47875403	120.4349423	38.73596745	22.360904	25.22135453
Pamr1	5.512172815	5.952880444	5.897797524	9.527034343	14.10863897	13.62605599
Paqr9	0.147835027	1.496312564	0.157043492	0.023975941	0.03385466	0
Pax5	1.457443689	0.10574566	0	7.13681207	5.426274244	9.742523174
Pck1	0.868398923	0.537136214	1.037801749	0.042251181	0	0
Pcp4l1	102.3000397	109.6542886	139.0483025	44.56252179	42.65912869	48.89522935
Pde4b	198.9365211	124.3958722	131.6200926	46.61858348	34.47817159	34.30234147
Peg3	19.83135247	19.8437862	9.372051692	2.21925859	1.083819327	0.386242648

Peg3os	25.08096719	28.64937227	13.39136186	2.300027296	2.165132713	0.295777029
Pfkl	66.38871219	50.27837736	62.86292144	15.47764086	16.12345265	18.44687407
Pgf	11.17632512	20.73319077	23.13520475	3.088506068	4.453840182	4.259048543
Pglyrp2	0.552732745	0.266404083	0.377461158	1.15254525	1.600300486	1.689640416
Phactr4	78.76976933	41.07051285	42.06919848	14.538845	11.76758791	10.13533171
Phf11a	0.251668935	0.094343248	0.401017633	1.142841398	1.325557463	1.700608695
Phospho1	26.88017475	31.03230975	36.95660931	13.65699744	10.99026844	14.33279735
Phyhipl	5.339701312	8.105808883	5.892783143	2.27667892	1.944393476	1.359984746
Pik3cd	10.30981071	5.17676276	6.798945761	24.95290325	14.18030379	17.70666668
Pik3r3	19.08999062	12.94255879	13.74444395	7.222824193	3.571145695	3.885767767
Pkn3	72.72098799	30.20277897	40.26175079	27.0006237	19.5420279	20.33858115
Pla1a	2.387965063	15.40136796	14.76180985	0.531928242	1.146410716	0.907259327
Plac8	37.37259573	85.52757634	67.35214066	144.116593	117.5009642	196.6641016
Plat	66.61772119	67.04876268	80.84623182	21.65182796	33.59326254	35.46696001
Plbd1	16.14034217	8.671358432	17.05401635	48.06951536	44.43329811	50.15847996
Plcb4	39.6722356	27.09128206	22.84468128	20.31256425	10.08198563	9.424488007
Plcg2	10.96752684	5.082287013	5.516527196	26.35931055	14.30702031	17.85810589
Plcl2	3.743225948	3.563245034	1.787140213	7.898832734	5.778942842	6.37881021
Plcxd2	0.433477473	0.400441247	0.320690218	1.144910836	1.074217547	1.604057243
Pld4	26.01350878	16.2097726	21.68617399	66.83154248	40.79281154	57.30044425
Pld5	1.254294531	0.492067634	0.604238348	0.198691224	0.040079605	0.262811639
Plin1	0.444623875	1.066728666	0.566782751	0.144218541	0.08145615	0.133531905
Plvap	79.47116562	122.4815897	123.624427	460.1815043	561.2635798	580.9861918
Podxl	534.8798406	389.3576644	333.1215107	261.6615481	155.8210909	139.5398889
Pou2af1	4.116738839	0.199128227	0.564279424	8.873340777	7.998130463	16.05276227
Pou2f2	1.404956232	0.632011898	0.84280617	5.516804088	2.180255875	2.159361694
Ppp1r14c	2.677655095	6.073253144	3.585432351	5.233053167	20.55993239	17.23217597
Ppp1r2	89.45570428	133.4787447	132.0793015	47.70873929	56.85023437	58.84358397
Pradc1	14.81183015	28.09113887	28.34992507	6.822783302	10.11564705	10.26547128
Prf1	0.857152861	0.497529729	1.409874392	3.01345132	3.799171934	6.103461239
Prg2	0.948813907	4.725493427	2.159816529	7.386192138	13.40935415	18.01310345
Prg3	0	0	0	0.465876036	0.493371917	1.078386218
Prkcb	1.949073139	0.737884092	1.12748832	6.685075749	4.383888979	5.91152051
Prss34	0.560460095	0.525249826	0.297685239	2.317838636	2.246072947	2.524807207
Psd4	4.390171007	3.02780784	2.202985187	10.77440302	7.465230877	8.795940205
Pstpip1	5.246705844	4.002681768	5.964640805	13.73403057	9.949356022	12.30167185
Ptk2b	18.44303653	7.821508968	9.825826697	38.94821535	22.90367624	27.84256079
Ptpn22	2.69787818	2.657574711	3.451662406	7.185923814	9.443208058	12.95203919
Ptpn6	24.820771	15.98073678	17.27995433	60.90471227	41.29753049	50.32713098
Ptprc	35.1625238	14.04562162	15.93987291	85.6278571	49.29178161	52.10864603
Ptprcap	4.095024426	6.039752142	5.990299648	12.67295707	23.24446556	38.10489175
Ptpro	1.153035073	0.624695466	1.3142618	3.578927987	1.942779872	2.27487075
Ptprr	10.34142261	13.15118196	20.07038041	0.438716307	0.871142302	1.269397818
Ptprt	0.265912396	0.440702467	0.416280064	0.108949324	0.096149571	0.021015864
Ptx3	11.67187793	4.120669606	8.757707666	1.696466254	1.989445673	1.730495679
Pyhin1	4.514879755	2.009076831	2.536070526	12.75335648	8.635842641	11.34009299
Qsox1	163.8573428	319.4469114	261.9842669	48.27631757	75.50425243	67.90111284
Rac2	16.12510396	13.84278963	18.06349322	41.2481747	45.34048914	59.83249754
Rag1	0.008516013	0	0	0.157448845	0.14041384	0.191818211
Ralgps2	2.014398186	1.504307256	1.497065033	4.145034549	4.299418431	4.232435354
Ramp3	32.14100172	37.66312269	54.54978381	8.961818067	10.92873346	18.22989381
Rap1gap2	1.869482699	1.534483362	1.242383074	6.871426234	3.019138517	4.310684879
Rasal3	4.293863068	1.945467076	2.264253673	10.28037765	9.146880974	10.22831878
Rasgef1b	25.09823612	21.78755916	18.84836416	50.72680175	69.75223874	59.3503857
Rasgrp1	1.802255223	0.600544209	0.709079824	5.499390545	5.869821105	5.462749697
Rasl10b	0.179467448	3.816443209	1.455844155	0.158760542	0.358678502	0.22049451
Rassf6	0.435086488	0.260961712	0.4621875	1.15722489	1.434759803	1.960013766
Rassf9	19.54783197	13.87175368	13.75817405	11.53301642	7.700336745	4.952659274
Rbpjl	1.61870125	3.588807935	2.431904165	4.185344187	11.3748074	9.062988737
Rcor2	2.707128004	1.649086248	2.516282772	0.905526304	1.007403068	0.889240274
Reep1	18.2533842	11.93681733	13.6733044	9.222880065	6.737424906	6.263170076
Reep2	3.862887297	4.510057908	5.168532908	1.176451887	1.499114583	1.461224524
Rel	6.220008108	3.064987223	3.011874179	14.71439891	9.059790037	8.614048118
Ret	4.579979217	1.896709249	2.6134339	0.67753835	0.659061364	0.627332563
Retn	0.264976468	2.483296225	0.985185362	0	0.060680398	0
Rgs1	37.23590114	20.16959334	23.27048037	50.07050858	97.2210383	58.28697781
Rgs12	80.24322565	57.30874448	63.44604796	40.87818746	33.23841697	28.61538388
Rgs4	25.6448448	23.71521719	26.64733311	5.299082495	3.688154988	2.174831098
Rgs5	19.21365021	46.53463758	39.72032734	2.932778494	1.380386804	1.555731583
Rgs7bp	0.974490212	0.832901303	0.714281122	0.312925528	0.374910735	0.438996449
Rhag	0.220699312	5.7913556	1.094083226	0.071586198	0.033693865	0
Rhoh	1.16792652	0.746871366	0.291923589	3.052920415	4.169208216	4.797133693
Rhpn2	15.77132642	13.03780343	13.94680013	7.135056082	5.94509095	6.174850775
Rin1	3.848778963	2.844801359	2.29708073	1.733633947	1.364117428	0.980257204
Rltpr	2.517593907	0.918264233	0.505969799	4.833440615	4.799276635	4.904417834
Rnase6	5.143743314	2.981810406	3.548875002	19.55673432	15.66528775	13.61651394
Rnf43	0.592831833	0.859309203	0.419839075	1.435775693	1.864440762	2.373900536
Rprml	1.059576298	0.238322309	1.181854828	5.413038431	5.313949982	4.295940621
Rps6kl1	6.477214163	1.71396439	2.71417512	1.155888268	0.708289189	0.908691465
Rsad2	5.806870031	27.53935492	5.690389006	3.504224766	3.58734175	2.906585628
Ryr1	0.081333965	0.041576864	0.07069104	0.255421343	0.294625017	0.333090913
S1pr5	0.614596721	0.051198701	0.145084269	3.123166657	1.469997836	1.94833486
Saa3	7.914695723	12.50945274	25.5639301	4.57935964	3.086129608	3.372749364
Sash3	9.253086266	4.152563334	5.253268917	24.03922148	13.61988435	18.81228899

Satb1	1.999645876	1.58076365	1.733135037	5.308262106	6.023915079	8.405113444
Sbk1	3.414259344	2.653461489	2.786543348	7.779173454	7.34200189	12.00455743
Sbk3	0.486490742	2.228978959	1.722645543	0.245464348	0.420874002	0.243509325
Scml4	1.125093619	0.673028229	1.106171997	2.399261584	2.401076662	2.857723479
Scn8a	0.119230139	0.145261534	0.079160525	0.024170987	0.006826014	0
Sdcbp2	16.94048766	7.949532138	9.062577794	2.450931169	3.070046161	3.843197237
Sell	14.60818349	8.035394502	14.894266	35.45407459	36.27736142	48.61205707
Selplg	25.97573268	16.77701197	24.32696574	56.43180663	47.82789803	54.23487555
Sema3c	351.5682283	227.3037864	253.7914751	196.7105501	103.2644681	105.4885343
Sema4d	4.361984609	2.389516816	2.947503138	16.46747218	8.912887455	14.1324009
Sema4f	0.679616549	0.840734944	0.866338852	1.565130705	3.206064669	3.214672758
Sema6b	9.268002247	12.88007138	10.23971351	4.933093634	6.200416726	4.374554906
Sema7a	186.4900874	315.821959	221.4465492	70.95798798	99.55120444	92.729937
Sept5	4.886305591	21.86066565	11.4875432	2.936010134	4.769416501	5.051988469
Sh2d2a	1.062337699	0.832274799	1.027070382	2.764388745	2.738933078	5.162111078
Sh3gl3	2.992916213	2.027631076	2.413235001	1.263189594	0.594552316	0.64977049
Shc4	0.377050885	0.424035711	0.488154383	0.022931346	0.14570849	0.053080317
Shisa9	0.96140581	0.448305715	0.672557236	0.114088656	0.144986566	0.052817327
Shroom2	10.16934939	9.373468576	8.162758567	3.118903488	3.557421602	2.432481249
Sidt1	1.344929015	0.778724432	0.721424297	2.708157058	2.305371379	2.699442172
Siglecg	3.200910041	0.102850852	0.364316878	10.4121606	5.843199848	9.269823562
Siglech	1.208367863	0.266459653	0.377539894	4.818644077	3.483414306	5.016610398
Skap1	1.659712572	1.808202508	2.066125857	3.482423367	5.84371626	8.878713198
Slain1	0.91200903	1.165517863	0.726612728	2.158140981	2.819512467	3.828369468
Slamf6	3.277877131	1.359500492	2.222586473	6.718612275	4.024727356	7.016683476
Slamf7	1.556567404	0.321198788	1.001216433	3.863098677	4.100908531	2.830594351
Slamf8	0.751675891	0.125236037	0.266165625	3.413398352	1.874369894	2.006644159
Slamf9	11.95387143	11.49109	11.06505132	21.23703029	24.12610218	29.0481458
Slc16a12	1.643630086	2.706651716	1.683652326	0.885375135	1.169516669	0.793324435
Slc16a9	7.794532305	11.36509128	11.17551993	47.18348165	39.48837769	42.86857614
Slc22a23	5.087864688	5.72187045	3.445548294	13.8802328	11.54733117	11.82847875
Slc24a3	6.937345133	29.303476	16.39617586	3.795198134	5.290507899	6.429822679
Slc26a10	76.89529365	32.93001025	36.60708625	3.692619694	1.972893691	1.963612287
Slc26a9	1.685075625	2.105616887	0.546955735	3.567903301	6.067006208	4.498411082
Slc28a2	1.512935993	1.014334911	1.066298765	4.232607295	2.978282207	2.555849919
Slc2a1	27.07176245	49.2685971	46.7963047	11.25918408	19.11432529	16.61215815
Slc2a4	0.362253442	2.036971687	2.052361447	0.097917337	0.276523712	0.090661634
Slc36a4	19.49795435	16.28908992	14.9503459	11.75477152	7.332146854	6.340042465
Slc44a1	53.43212479	64.61079208	64.98728485	23.26573269	17.23530624	17.63924693
Slc46a3	21.06582984	25.9597079	29.75426055	8.423220574	5.866323195	7.291801731
Slc4a1	1.922234416	103.1752275	20.00863526	0.218628968	0.291559309	0.843452078
Slc5a12	0.579858283	0.073824364	0.10459986	3.183220743	1.683667704	2.168613703
Slc6a6	249.0103577	177.3130344	155.1449184	67.22404665	29.99205137	36.7865788
Slfn1	2.599585145	2.476395951	2.826486875	7.707871346	5.168715108	7.439819621
Slfn14	0.306659231	6.518089423	1.221602491	0.179042802	0.021067762	0.138146399
Slfn8	1.2460258	0.700647388	0.360992521	3.885465375	2.35408476	1.849804732
Snca	5.019121445	22.49140467	22.39337714	1.314927985	1.591468216	1.565346141
Snph	1.817385266	0.403391867	1.143111316	0.162884974	0.065713677	0.053862554
Sox17	65.73875277	72.27724928	75.31414729	27.11269118	33.1767815	25.74090786
Sp5	0.027083114	0	0.086310265	0.316249183	0.558190211	1.220062615
Spag9	77.05574504	43.91601779	42.97934926	39.61170442	21.2795342	19.44017151
Sparcl1	841.656138	831.175823	891.0808285	211.3389972	283.5905789	256.1755605
Spib	2.385073539	0.183403816	0.649650485	10.414173	9.999458712	12.39747401
Spin4	3.780779335	1.865944977	2.080375001	0.886667744	0.934327006	0.551394677
Spn	5.310384265	3.4553943	4.780209907	19.97527847	12.33268662	19.23621661
Spns3	0.312222593	0.210677446	0.099501168	1.032982145	0.900898748	1.265873652
Spta1	0.060813754	2.994042747	0.710619468	0.013150397	0.074274828	0
Ssu2	2.10663981	2.997701356	2.192189252	0.334683087	0.413508661	0.774707435
St6galnac3	22.77579026	14.56606324	15.79317887	11.38008643	6.086126751	6.322091251
St8sia1	0.170474577	0.071006536	0.120728822	0.466937788	0.459794836	0.654194997
Stab2	7.108762915	2.293145289	2.091885744	1.291068917	0.546906986	0.597700169
Stap1	3.649568784	1.686717753	1.274595951	10.16750867	9.342217294	12.16174739
Stat4	1.828968735	0.985587866	0.910731089	3.5594742	4.947538878	5.407034287
Stfa1	0.407607341	7.639997476	9.092930926	0.13221183	0.186686583	1.224148949
Stfa2	0.134263096	9.059628213	8.557570926	0	0	0
Stfa2l1	3.978273908	28.07828251	43.71802538	4.405144871	2.45037413	4.9439059
Stk10	13.89325298	5.690796622	6.172230266	33.98883887	16.53609617	18.50894871
Stk17b	31.35932097	23.49837613	26.99981503	58.75440465	67.75094138	96.15731072
Susd1	1.430604866	0.930433441	0.823942148	3.589260266	2.415650724	3.571765704
Susd3	2.059449831	1.870680554	2.776734834	5.048574171	4.679922783	5.709280453
Syk	22.89183239	10.12815568	11.70684468	45.36892065	27.23527777	34.84412738
Synm	4.924106724	3.670151444	3.704527414	12.92737889	8.055480945	7.545961637
Sytl3	0.693729107	0.572128801	0.442164394	1.620133234	1.874619096	2.083445687
Tagap	5.428227287	3.696010222	4.001137356	15.81040292	22.98429854	17.88268304
Tbc1d10c	8.782134602	3.921546857	4.733178371	18.41104219	10.46973166	15.12683431
Tbc1d24	10.95948988	5.440839429	5.461468757	2.930331629	1.627949655	1.366127729
Tcf23	7.533662407	4.703451458	5.163652069	1.724911418	1.617404116	1.247730458
Tec	4.353938773	1.968196569	2.176536641	8.245029787	7.947193799	6.634142049
Tekt5	0.702729117	1.580594303	1.017956272	2.424428847	3.028354564	5.899736976
Tespa1	1.139888929	0.452446604	0.213686688	3.784348991	2.165079458	3.020627276
Tex15	0.087810747	0.197505929	0.02152624	0.30235122	0.491895165	0.486864187
Tgif1	9.217759041	6.879083442	9.137623182	19.13798371	22.3540776	23.63285011
Tgm3	0.041114833	0.061650945	0.13102748	0.386745125	0.301293404	0.493913379
Thbs2	0.959022114	0.905962821	1.161384626	0.326622605	0.316251232	0.345622601

Thbs3	3.175999229	5.738903036	6.027401276	9.792398126	24.54069322	26.28401245
Themis2	16.19792877	6.382012683	10.18172517	35.54584212	24.62000912	23.35032818
Thsd7a	1.01195316	1.358697151	1.349217751	0.783751725	0.368892687	0.32562362
Tiam1	20.7931318	11.02456143	18.56810722	6.272008476	2.521777105	3.215314026
Timp3	372.5110809	469.9200703	419.6353258	116.7627318	231.537705	191.1338207
Timp4	1.082922894	15.51653273	13.54888295	0.546400866	2.920801236	1.626147015
Tinag	0.266010724	0.373948354	0.211935158	1.391320506	6.39631078	5.242769064
Tlr1	1.004203549	0.292791521	0.888961813	2.895326509	2.325207671	2.597007544
Tlr12	0.250506003	0.120737956	0.171070534	0.992463469	0.88508497	0.56425013
Tlr9	0.916284707	0.404825219	0.521442308	3.327654812	1.348919473	1.474198388
Tmc7	6.408679683	6.937178923	7.089851328	0.639607462	0.851038189	0.683321959
Tmeff1	38.23430306	26.10263642	35.65006252	18.28572979	16.23332338	12.99141857
Tmeff2	3.418823362	1.262487274	1.788786052	8.40802298	6.006287158	6.589653551
Tmem163	0.518969273	2.182301385	1.366253427	1.88826017	8.060825504	7.725221257
Tmem200b	8.042261444	18.36856152	24.04427574	4.679333716	5.809904478	7.096489746
Tmem252	35.61691272	57.79983923	65.50139275	10.55009587	15.49923268	24.62866364
Tmem8	2.148403389	3.762505716	2.560969861	5.266329704	7.300992564	6.427577343
Tnfaip8l1	4.082392908	3.402819341	3.979541863	1.799310759	2.012741853	1.730889677
Tnfrsf13c	0.595942178	0.804244016	0.094959405	2.928498961	3.07063188	3.624277292
Tnip3	0.25616501	0.440602242	0.192085567	0.513202193	2.256782721	1.561285221
Tnk1	33.74085806	39.86939801	33.16324497	16.09880216	17.08530094	20.89683083
Tnni1	0.917665361	0.91734835	3.086947825	0.049609107	0.210148092	0
Tpi1	110.4294256	142.2621931	173.6443923	54.78128202	73.59952354	80.22189845
Traf1	4.763308158	7.911685644	8.329553069	11.31437561	19.9366817	21.23806286
Traf3ip3	4.311041783	2.469293291	1.706672561	9.901238568	5.81307339	7.961296842
Treml2	1.91452207	1.189867338	1.60561211	3.419562649	3.85934866	5.220212018
Treml4	2.571157522	1.171261963	2.074414041	17.67198623	9.167434533	15.24819573
Trib3	9.747068713	8.221248186	9.883556319	3.206475292	4.451532914	3.991764101
Trim10	0.733632694	18.09423652	5.159700671	0.073850171	0.069518923	0
Trim58	0.10363739	2.097932154	0.660557026	0.100847709	0.035599905	0
Trim7	2.037240988	1.122173568	0.529993046	5.421265736	3.941738224	5.0570077
Trim9	0.060725249	0.443899987	0.290284884	0.014772666	0.020859394	0.034195019
Trpv4	24.78764778	18.25433644	21.99290423	6.047236297	4.136386717	3.410237633
Tspan8	157.8047448	171.3001883	174.0954028	60.09383808	90.80155714	94.30370572
Tspo2	0.485931889	11.20293277	3.097200002	0	0.083459884	0
Ttc7	7.285293051	7.279700824	7.059300654	18.08222374	13.37501155	17.2967074
Tubb2a	89.52462469	117.4737436	113.1062947	47.58604413	52.80124659	65.83480674
Tubb2b	14.36084221	22.53075801	18.26876653	5.031891431	5.399923971	4.126566618
Txk	1.162367777	1.15034657	1.185379452	2.035943114	3.034516657	4.084338143
Uaca	46.81470347	36.03166362	38.58560029	24.39977077	13.97510124	14.27150885
Ube2l6	37.17974545	74.32356997	65.8985405	23.94664123	24.02375009	25.08666887
Uhrf1bp1	13.5171609	3.951359965	4.478865008	3.535073015	1.69423467	1.821719966
Unc5b	30.59887308	16.13361199	17.95427097	11.03030201	5.213860009	5.625409888
Upp1	29.69414161	21.51247749	37.92338946	13.49147502	12.47948624	16.1991982
Vav1	8.448138085	3.892292764	5.210920108	17.97953999	11.77639387	11.66289702
Vcan	0.680483284	1.060955303	1.663421907	0.526723144	0.403748339	0.435440007
Vegfc	88.64704848	105.1942764	126.531261	20.33124134	16.96773606	17.54613493
Vil1	4.937754724	3.483466286	4.529170599	0.709201729	0.325458739	0.328324838
Vipr1	0.926859071	0.703590465	0.553833002	2.029297849	1.464549004	1.617964308
Vnn1	1.363415064	1.586184906	1.423369154	2.287439766	3.843610923	5.930231497
Vstm5	4.028095488	2.63449249	3.004404743	0.889570137	0.431783152	1.029565917
Vwf	227.5331792	255.5971966	187.0571278	130.7293629	72.74373501	81.90351874
Wars	160.9832683	146.0843838	95.42448496	76.33139687	55.21726767	63.00779137
Was	8.028086779	6.58834854	6.914713471	19.74110767	12.63317198	17.71625305
Wbscr27	4.335984292	8.248689676	7.683953759	3.568634534	3.229418129	3.103390093
Wdfy4	7.761338923	2.243149347	2.000434964	18.63273154	9.745305727	10.62395893
Wfdc1	139.3354615	148.8305493	191.5208531	45.43133233	44.52467009	61.4996096
Wnt9b	1.043116405	1.158966822	1.001286928	0.220128746	0.103609194	0.056615873
Xkrx	0.296415916	0.222235139	0.251903445	1.172977463	1.357602497	1.157276512
Ypel4	1.05732188	17.32653896	1.925453458	0.073490086	0.103769936	1.360888991
Zap70	9.914300827	7.886970713	8.780237601	3.290281605	4.244461133	4.889397566
Zbtb7c	2.732835456	1.816086889	2.018946605	1.293374079	0.699788857	0.895353037
Zc3h12d	0.985175558	0.343305237	0.442200403	3.024494997	1.944678508	2.625355356
Zcchc18	0.204845048	0.115185535	0.108802299	0.431884029	1.219663228	0.692102246
Zfp365	0.560328438	1.590382937	0.680263566	1.596790933	3.537879578	3.846421503
Zfp46	13.48488502	21.29471766	19.00871435	10.45223328	8.010492983	7.558994361
Zfp831	0.486551384	0.091196869	0.129214521	1.324545438	1.352977925	1.04374148
