# Supplementary material for: Endothelial senescence mediates hypoxia-induced vascular remodeling by modulating PDGFB expression
Source: Front Med (Lausanne). 2022 Sep 20;9:908639. doi: 10.3389/fmed.2022.908639 (PMC9530050; doi:10.3389/fmed.2022.908639)
Supplement: Supplementary file 7 [file Data_Sheet_4.PDF]

Biological Processes	PValue	Fold Enrichment	Genes
immune system process	3.16E-17	3.478141043	CD86, ITK, H2-T23, PGLYRP2, NRROS, LST1, PIK3CD, PTPN22, LRMP, TNFRSF13C, IFI30, CD3E, PSTPIP1, LAMP3, CTLA4, EOMES, CR2, RSAD2, SYK, PRKCB, CD180, CD8B1, TLR1, LAT2, HCK, ZAP70, OAS2, CD8A, TLR12, BTK, TLR9, IRF5, SKAP1, CFD, CSF1R, UNC93B1, TXK, LY9, CD79B, CD79A, KLRK1, BTLA, PTK2B, SLAMF7, SLAMF6, CD300LG, CD55, SEMA4A, MX1, PRG2, LY86, CD4, TEC, BPIFB1, THEMIS2, BCL6, GPR183, CD7, HC, LAT, MYO1G
adaptive immune response	4.98E-16	5.498818337	CD86, H2-T23, ITK, UNC93B1, TXK, PIK3CD, TNFRSF13C, LY9, CTSS, CD79B, CD79A, KLRK1, LAMP3, BTLA, SLAMF7, PTK2B, CTLA4, SLAMF6, RAG1, EOMES, SEMA4A, SYK, PRKCB, CD8B1, LAT2, ZAP70, CD4, TEC, CD8A, GPR183, CD7, BTK, SKAP1, LAT, MYO1G
immune response	9.6E-13	3.532644217	MCPT8, NRROS, MCPT4, LST1, WAS, CXCR5, CTSW, FYB, CXCL15, CX3CL1, CTSS, H2-DMB2, CCL4, CCL3, H2-OB, CCR9, BLNK, CTLA4, CCR7, H2-OA, TINAG, RELT, H2-EB2, CCL22, PRG2, PRG3, TNFRSF1B, VAV1, TLR1, IL4, ZAP70, CD4, CXCL12, FASL, OAS2, CD28, TLR9, TLR12, FAS, IRF8, LCP2, LTB, MAP3K14, LAT
transmembrane receptor protein tyrosine kinase signaling pathway	2.08E-12	5.677922705	RET, PTPRT, SHC4, BLK, ITK, CSF1R, FLT1, TXK, STAP1, EPHB1, NTRK2, EPHA7, YES1, SYK, TNK1, PILRA, ZAP70, HCK, DOK2, TEC, LCK, BTK, LCP2, CSPG4, MET, LAT
inflammatory response	6.11E-11	2.983702674	CSF1R, SEMA7A, CIITA, LXN, NRROS, NCF1, PIK3CD, TNFAIP3, PTGS2, AIF1, CXCL15, CX3CL1, NPPB, PSTPIP1, CNR2, MECOM, CXCR3, CCL4, CCL3, CCR7, RELT, CCR3, CCL22, NOS2, CD180, LY86, TNFRSF1B, TLR1, ZAP70, HCK, BMP2, VNN1, CXCL12, THEMIS2, BCL6, TLR9, CHIL1, REL, TLR12, FAS, ACKR2, CHIL3, CSPG4, HC, NFKBID, LAT, CHST2
cell surface receptor signaling pathway	3.86E-10	3.490117574	VIPR1, CALCRL, LAMA1, PIK3CD, CD3G, CD3E, ITGAL, CD3D, SPN, CD79B, CD79A, UPK1B, CXCR1, TSPAN8, CXCR3, TSPAN7, BTLA, PTK2B, IL12B, FCER1A, CD37, PDK1, CD53, EDN1, FCER1G, SYK, FZD6, TNFRSF1B, AGT, CD4, CD8A, LCK, CLCF1, CD247, CD22
T cell receptor signaling pathway	5.06E-10	7.279388084	ITK, TXK, RFTN1, PTPN22, CD3E, FYB, ZAP70, PTPRC, THEMIS2, CD28, PDE4B, PLCG2, CLEC2I, LCP2, BCL2A1D, CD247, SKAP1
positive regulation of cell migration	8.56E-10	3.550046405	RET, CSF1R, SEMA7A, FLT1, SEMA3C, LEF1, PIK3CD, CXCR4, IRS2, SEMA3F, AIF1, CORO1A, CX3CL1, AQP1, PAK1, PODXL, CCL3, PTK2B, SEMA6B, SPAG9, SEMA4A, EDN1, SEMA4D, MCAM, RDX, SEMA4F, VIL1, TIAM1, MMP14, BMP2, CXCL12, NUMB, SNAI2
B cell activation	1.96E-09	9.789521906	CD86, CR2, PRKCB, CXCR5, PIK3CD, IKZF3, LAT2, IL4, CD79A, BANK1, BLNK, MS4A1, GAPT
positive regulation of T cell proliferation	2.6E-09	6.141983696	CD86, SASH3, SLC4A1, TNFRSF13C, CD3E, ITGAL, AIF1, CORO1A, SPN, SPTA1, CCDC88B, IL4, CD4, PTPRC, CD6, CD28, IL12B, CCR7
cell adhesion	3.82E-09	2.43146571	RET, CYFIP2, ACHE, LAMC3, COL12A1, ICAM2, STAB2, ITGAL, HAPLN1, CX3CL1, PSTPIP1, CDH4, ITGB7, TINAG, CD34, EPHB1, KLRA4, CD96, EGFL6, VWF, GP1BB, ITGA3, KLRA8, AJAP1, VCAN, TMEM8, ADAM15, COL8A1, CD226, CDH16, FREM2, CX3CR1, COL15A1, NLGN2, SELPLG, LAMA1, THBS2, LY9, THBS3, PODXL, 9430020K01RIK, ADAM23, PTK2B, MCAM, MYBPH, LYVE1, CD2, CD4, CD6, SELL, CASS4, FAT2, ACKR3, CD22
erythrocyte development	1.14E-08	9.705850778	BCL6, TMOD3, HBB-B2, HBB-B1, HBA-A2, HBB-BT, RHAG, HBA-A1, BPGM, HBB-BS, ANK1, GATA1
B cell receptor signaling pathway	1.52E-08	6.352920509	BLK, SYK, PRKCB, RFTN1, NFATC2, LAT2, CD79B, CD79A, ZAP70, PTPRC, TEC, LCK, CD19, PLCG2, CTLA4, PTPN6
chemotaxis	1.66E-08	4.256591337	CX3CR1, CCL22, FLT1, CXCR5, PIK3CD, CXCR4, IL16, LSP1, CXCL15, CX3CL1, CXCL12, CXCR1, CXCR3, CCL4, CCR9, ACKR4, CCL3, RAC2, ACKR3, CCR7, ACKR2, DOCK2, CCR3
signal transduction	5.81E-08	1.740092769	VIPR1, CHRM1, CD83, GPR65, IRS2, GPR174, RGS5, GPR176, EDNRB, RASSF2, GRB14, RGS1, RASSF5, RASSF6, GPR171, ADORA1, GRB10, UNC5B, HMHA1, ANK3, ANK1, SIGIRR, TIAM1, ACAP1, APLNR, SPARCL1, RIN1, PDE9A, NDRG4, CALCRL, ARHGAP15, STK4, RASAL3, NDRG1, PLCG2, RHPN2, NTSR2, PACSIN1, GPR17, BCL11B, RASL10B, FZD6, RASSF9, PILRA, ICK, GPR183, CSPG4, GPR18, LAT, ARHGAP9, LSP1, HTR2A, ARHGAP4, AKAP12, GPR132, TAGAP, BDKRB2, CCR9, FCER1A, CCR7, CD34, CCR3, RPS6KL1, FCER1G, WNT9B, TRAF1, TLR1, GPRC5B, ADORA2A, RASA3, TLR12, TLR9, CD48, GPRC5C, PTGER4, CX3CR1, PKN3, CXCR5, CXCR4, FYB, GNG2, CNR2, OLFR56, CXCR1, P2RY2, CXCR3, STAT4, PTK2B, S1PR5, ZFP831, P2RY10, PPP1R14C, NOS3, PLCL2, ACKR4, FAS, ACKR3, KRAS, RGS12, ACKR2
receptor internalization	1.32E-07	7.097403382	GRIA2, ACHE, CALCRL, FCER1G, RAMP3, SYK, CAV1, DNM3, CXCR1, ACKR3, EZR, SNCA, PICALM
positive regulation of peptidyl-tyrosine phosphorylation	1.35E-07	4.324388961	YES1, SEMA4D, SYK, ADIPOQ, IGF2, VEGFC, LRP4, HTR2A, CD3E, GATA1, AGT, IL4, CD4, TEC, EHD4, FCER1A, HCLS1, PTK2B, CSPG4, FGFR3
T cell activation	1.86E-07	7.707587383	CD2, HSH2D, CD4, SATB1, CD8A, TREML2, CD28, WAS, CD48, DOCK2, CD3D, VAV1
protein autophosphorylation	2.74E-07	3.222024234	CSF1R, FLT1, TXK, STK4, HK1, STK10, PAK1, TRIM24, EPHB1, MAP4K1, NTRK2, YES1, RIPK3, SYK, NEK6, TNK1, VRK1, ZAP70, HCK, TAOK3, LCK, STK17B, BTK, SIK1, MET, FGFR3, FGFR2
response to lipopolysaccharide	3.41E-07	3.103901518	PTGER4, NCF2, PTPN22, PTGS2, CXCL15, NPPB, EDNRB, CNR2, PLCG2, CCR7, PCK1, RELT, TIMP4, SNCA, CD96, EDN1, ACE, KCNJ8, NOS2, IL10RA, FMO1, TNFRSF1B, FASL, CD6, TRPV4, CYP1A1, FAS, ALDOA
protein phosphorylation	4.67E-07	2.085241378	RET, ITK, FLT1, HK1, STK10, TRIM24, EPHB1, PDK1, MAP4K1, EPHA7, RPS6KL1, RIPK3, SYK, PRKCB, VRK1, HCK, ZAP70, LCK, BTK, SIK1, TRIB3, MET, BLK, CSF1R, LAMA1, PKN3, TXK, STK4, PAK1, NUAK2, CCL3, STAT4, PTK2B, MARK4, IP6K3, MARK1, NTRK2, CDK19, YES1, MLKL, DMPK, NEK6, TNK1, LIMK1, ICK, SBK3, BMP2, TEC, SBK1, TAOK3, STK17B, POMK, MAP3K14, FGFR3, FGFR2
negative thymic T cell selection	1.06E-06	12.478951	SPN, ZAP70, PTPRC, CD28, FAS, CCR7, DOCK2, CD3E
phosphorylation	1.38E-06	1.998263396	RET, DGKG, ITK, CIITA, FLT1, PIK3CD, HK1, STK10, EPHB1, PDK1, MAP4K1, EPHA7, RPS6KL1, RIPK3, SYK, PRKCB, VRK1, HCK, ZAP70, PKM, LCK, BTK, SIK1, MET, PRPS2, BLK, CSF1R, PKN3, TXK, AK3, AK4, STK4, PAK1, NUAK2, PTK2B, MARK4, IP6K3, MARK1, NTRK2, CDK19, YES1, DMPK, NEK6, TNK1, LIMK1, ICK, SBK3, PFKL, TEC, SBK1, TAOK3, STK17B, POMK, MAP3K14, FGFR3, FGFR2
peptidyl-tyrosine autophosphorylation	1.54E-06	6.39165783	BLK, HCK, ITK, ZAP70, TEC, YES1, SYK, LCK, TXK, TNK1, BTK, PTK2B
innate immune response	3.06E-06	2.238411836	CFD, BLK, ITK, CSF1R, NRROS, UNC93B1, TXK, PIK3CD, LY9, PSTPIP1, KLRK1, SLAMF7, PTK2B, SLAMF6, CD55, CR2, YES1, FCER1G, RSAD2, SYK, MX1, TNK1, CD180, LY86, TLR1, ZAP70, HCK, BPIFB1, VNN1, TEC, ADAM15, OAS2, LCK, BTK, TLR9, REL, TLR12, PTX3, IRF5, HC, TRIM10
regulation of cell shape	5.27E-06	3.299291002	CSF1R, SEMA4A, SEMA4D, SH3KBP1, RDX, LST1, VRK1, ARHGAP15, CORO1A, SPTA1, VIL1, HCK, FMNL1, PALMD, RHOJ, CDC42EP3, CCL3, PTK2B, PLEKHO1, EZR, ALDOA
positive regulation of calcium-mediated signaling	7.08E-06	8.189311594	ZAP70, CD4, SYK, CD8A, CCL4, CCL3, FCER1A, CD3E, ITGAL
response to hypoxia	7.73E-06	2.84351097	RYR1, ALAS2, FLT1, CXCR4, PLAT, PLOD1, HIF3A, LOXL2, NPPB, PAK1, ADORA1, PTK2B, EDN1, NOS2, PRKCB, BNIP3, CAV1, ADIPOQ, VEGFC, PGF, MMP14, BMP2, CXCL12, CYP1A1, ALDOA
cytokine production	9.99E-06	7.86173913	ITK, CD4, MAF, TXK, REL, NFATC2, CD226, RASGRP1, BATF
T cell differentiation	1.17E-05	5.859019677	JAG2, CD4, PTPRC, LCK, RHOH, PTPN22, IKZF1, BCL2A1D, IL7R, CD3D, VAV1
regulation of cell proliferation	1.6E-05	2.597490902	BLK, ITK, CEBPA, TXK, TCF7, PKD2, PTGS2, NDRG1, CXCL15, MECOM, PTK2B, RELT, JAG2, YES1, NOS2, TNK1, TNFRSF1B, HCK, TEC, BCL6, LCK, BTK, MZB1, FAS, KCTD11, INHA, FGFR2
positive regulation of urine volume	1.97E-05	10.91908213	PTGER4, NPPB, EDN1, EDNRB, ADORA2A, HYAL2, NPR3
peptidyl-tyrosine phosphorylation	3.33E-05	4.36763285	CSF1R, FLT1, SYK, HK1, ZAP70, HCK, TEC, LCK, BTK, PTK2B, PTPN6, FGFR3, FGFR2
angiogenesis	3.9E-05	2.467072949	CALCRL, FLT1, HIF3A, PTGS2, SOX17, ANPEP, CXCR3, PTK2B, THSD7A, EPHB1, SEMA4A, WARS, SYK, NOS3, UNC5B, MCAM, CAV1, VEGFC, PGF, MMP14, COL4A2, ADAM15, COL4A1, COL8A1, ACKR3, CSPG4, FGFR2
positive regulation of interleukin-10 production	5.26E-05	7.595883218	SASH3, IL4, CD83, FCER1G, CD28, TLR9, IL12B, CD34
neural crest cell migration	6.15E-05	4.902445036	RET, SEMA6B, SEMA4A, SEMA7A, EDNRB, SEMA4D, SEMA3C, LEF1, SEMA4F, SEMA3F, PHACTR4
positive regulation of gene expression	8.46E-05	2.025092926	RET, PTGER4, LEF1, PIK3CD, PTPN22, IKZF1, PKD2, CD3E, LRRC32, SOX17, LAMP3, CCL3, TRIM24, STAP1, CD34, IL33, NTRK2, ITGA3, CAV1, RDX, NFATC2, ANK3, ETV4, AGT, BMP2, MAF, MYCN, LCK, TRPV4, CD28, TLR9, KRAS, SLC26A9, IL7R, EZR, MET, FGFR2
T cell costimulation	9.51E-05	6.98821256	SPN, CD5, CAV1, CD28, BTLA, TNFRSF13C, CD3E, ICOS
microglial cell activation	0.000104	8.492619431	TLR1, CX3CR1, IL4, TLR9, AIF1, CX3CL1, SNCA
positive regulation of inflammatory response	0.000119	4.159650334	PTGER4, IL33, GPRC5B, CDK19, ACE, TRPV4, HYAL2, CCL4, TLR9, CCL3, CTSS, CX3CL1
sensory perception of pain	0.000184	3.686962796	EDN1, IL1RN, NLGN2, ACE, HTR2A, PTGS2, AQP1, EDNRB, CNR2, P2RY2, POMK, IL12B, SCN3B

positive regulation of renal sodium excretion	0.000187	10.07915273	PTGER4, NPPB, EDN1, EDNRB, ADORA2A, AGT
mast cell activation	0.000187	10.07915273	FCER1G, RHOH, LCP2, CD48, NDRG1, FYB
intracellular signal transduction	0.000191	1.965434783	SHC4, DGKG, ITK, DGKD, PKN3, RASGRP2, STK4, RASGRP1, NUAK2, RASSF5, PLCG2, BLNK, MARK1, PDK1, MAP4K1, EDN1, SYK, PRKCB, PLCL2, HMHA1, VAV1, ICK, LAT2, TIAM1, ZAP70, PLCB4, TEC, RASA3, STK17B, BTK, PTPN6, SIK1, LCP2, CSPG4, MYZAP, LAT
defense response to virus	0.000234	2.615349012	CD86, IL33, SLFN8, RSAD2, KCNJ8, IFNGR1, UNC93B1, BNIP3, MX1, PRF1, ABCC9, NCR1, PTPRC, OAS2, CD8A, HYAL2, TAGAP, TLR9, IL12B, IRF5
negative regulation of smooth muscle cell proliferation	0.000267	5.172196796	NPPB, NDRG4, NOS3, CAV1, NPR3, ADIPOQ, IL12B, TNFAIP3, AIF1
positive regulation of MAPK cascade	0.000272	3.149735229	SPAG9, NTRK2, FLT1, IGF2, TNFRSF1B, AGT, BMP2, PTPRC, BANK1, BNIP2, FAS, RELT, FGFR3, FGFR2, NTSR2
positive regulation of protein phosphorylation	0.0003	2.492399181	PTGER4, CSF1R, SEMA7A, EPHA7, SEMA4D, LRRN3, ADIPOQ, IGF2, CXCR4, STK4, AIF1, RASGRP1, SPN, IL4, GPRC5B, PAK1, BMP2, EDNRB, CD6, DOK7, KRAS
defense response to protozoan	0.00033	5.823510467	CCDC88B, IL4, IRF4, TLR12, IL12B, IRF8, CD37, BATF
leukocyte chemotaxis	0.000357	6.948506807	CNR2, GPR183, CCL4, CXCR5, CCL3, IL16, CORO1A
regulation of ERK1 and ERK2 cascade	0.000409	5.635655291	TIAM1, EPHA7, SYK, CLCF1, PTPN6, EPHB1, RASGRP1, FGFR2
apoptotic signaling pathway	0.000452	4.28199299	SPN, FASL, DAPL1, CD5, CAV1, CD28, FAS, CD3E, RELT, UACA
regulation of immune response	0.000504	5.459541063	SPN, IL4, FCER1G, SYK, CLEC12A, PHF11A, TNFRSF13C, CD200
hemopoiesis	0.000525	3.301117852	CSF1R, GFI1, HBB-B1, HBB-BT, HBB-BS, IKZF1, ZFP36L2, CXCL15, ADD2, SPTA1, TTC7, CD34, PICALM
positive regulation of interferon-gamma production	0.000525	4.199646971	SASH3, KLRK1, TXK, PDE4B, IL12B, IRF8, CD226, SLAMF6, CD3E, IL27RA
positive regulation of B cell proliferation	0.000646	4.570778564	SASH3, IL4, PTPRC, BCL6, CLCF1, GPR183, NFATC2, IRS2, TNFRSF13C
regulation of platelet activation	0.000889	17.4705314	TEC, FCER1G, SYK, TXK
cellular oxidant detoxification	0.000889	17.4705314	HBA-A2, HBB-BT, HBA-A1, HBB-BS
apoptotic process	0.000927	1.68575303	CYFIP2, SHC4, BEX2, NCF1, DAPL1, SH3KBP1, GPR65, PRF1, TNFAIP3, LSP1, HIF3A, STK4, PAK1, NUAK2, BCL2L11, EVA1A, MECOM, RASSF5, RASSF6, BNIP2, BCL2A1A, SH3GLB1, EPHA7, PEG3, UNC5B, PRKCB, BNIP3, GZMA, NEK6, TRPV2, GZMB, TRAF1, GULP1, VIL1, TIAM1, FASL, PLSCR3, STK17B, BTK, CHIL1, FAS, TRIB3, FGFR3, FGFR2
positive regulation of alpha-beta T cell proliferation	0.001107	9.926438296	ZAP70, PTPRC, SYK, CD28, CD3E
vasoconstriction	0.001345	6.896262395	EDN1, EDNRB, ACE, CAV1, BDKRB2, AGT
positive regulation of interleukin-4 production	0.001345	6.896262395	SASH3, H2-T23, IL33, PRG2, CD28, CD3E
positive regulation of tumor necrosis factor production	0.001349	3.701383771	CD2, SASH3, H2-T23, FCER1G, CCL4, TLR9, CCL3, IL12B, CCR7, RASGRP1
receptor clustering	0.001426	5.459541063	ARHGEF9, PAK1, DOK7, LRP4, ITGB7, CLEC2I, ITGAL
immunological synapse formation	0.001601	9.099235105	CD6, DOCK8, PRF1, DOCK2, EPHB1
cellular response to transforming growth factor beta stimulus	0.001717	3.580026926	CX3CR1, EDN1, YES1, PARP1, COL4A2, ARG1, NOS3, HYAL2, CAV1, ZFP36L2
positive regulation of protein tyrosine kinase activity	0.001726	6.551449275	CSF1R, GPRC5B, ACE, DOK7, UNC119, AGT
positive regulation of JUN kinase activity	0.00203	4.36763285	TIAM1, IL1RN, EDN1, PAK1, TAOK3, TLR9, PTK2B, FGD2
positive regulation of ERK1 and ERK2 cascade	0.002512	2.207048515	CSF1R, NDRG4, SEMA7A, CCL22, PTPN22, HTR2A, RASGRP1, CX3CL1, BMP2, TRPV4, GPR183, CCL4, CHIL1, CCL3, ACKR3, PTK2B, FGFR3, FGFR2, NTSR2
T-helper 17 cell lineage commitment	0.002905	12.478951	IRF4, SLAMF6, LY9, BATF
negative regulation of B cell activation	0.002905	12.478951	BANK1, TBC1D10C, FAS, TNFAIP3
antigen processing and presentation of exogenous peptide antigen via MHC class II	0.003009	7.799344375	H2-DMB2, FCER1G, UNC93B1, H2-OA, IFI30
membrane depolarization	0.003344	5.696912413	EDN1, ADORA2A, CAV1, ADIPOQ, SCN3B, SCN1B
chemokine-mediated signaling pathway	0.003348	3.573517787	CCL22, CXCL12, CCL4, CCR9, CCL3, ACKR3, PTK2B, CXCL15, CX3CL1
integrin-mediated signaling pathway	0.003418	2.817827645	SEMA7A, TEC, FCER1G, ADAM15, SYK, ITGA3, TXK, PTK2B, ITGB7, ITGAL, VAV1, LAT
cellular response to cytokine stimulus	0.003452	4.632337871	CD86, CSF1R, NOS2, LEF1, HCLS1, CXCR4, CCR7
positive regulation of angiogenesis	0.003597	2.526729748	CX3CR1, FLT1, NOS3, PRKCB, VEGFC, CX3CL1, PGF, AQP1, CXCR3, CHIL1, PTK2B, HC, CD34, CCR3
positive regulation of protein binding	0.003674	3.211494743	SPTA1, TIAM1, BMP2, ACE, CALD1, CAV1, TRIB3, STK4, CTHRC1, ADD2
semaphorin-plexin signaling pathway	0.004032	4.49609264	SEMA6B, SEMA4A, SEMA4D, SEMA3C, CRMP1, SEMA4F, MET
positive regulation of nitric oxide biosynthetic process	0.004056	3.882340311	KLRK1, PTK2B, PTX3, PTGS2, PKD2, AIF1, AGT, ASS1
stimulatory C-type lectin receptor signaling pathway	0.00449	10.91908213	KLRK1, FCER1G, SYK, PLCG2
negative regulation of blood pressure	0.004681	4.36763285	NOS2, NOS3, ADIPOQ, RNPEP, ADORA1, BDKRB2, VEGFC
cytolysis	0.004895	5.24115942	LYZ2, GZMA, LYZ1, PRF1, GZMB, HC
positive regulation of mast cell degranulation	0.005087	6.824426329	IL4, ZAP70, FCER1G, SYK, FCER1A
multicellular organism development	0.005165	1.400698582	RYR1, FLT1, TCF23, HIF3A, IKZF1, LCLAT1, ANPEP, CREB3L2, HEY2, STMN1, PHACTR4, SEMA6B, EOMES, EPHA7, EGFL6, TPI1, UNC5B, WNT9B, SHROOM2, PAX5, TNFRSF1B, HIC1, PGF, TMEFF1, MN1, B3GNT5, NUMB, TIMELESS, APLNR, KCTD11, SIK1, FREM2, HEMGN, CEBPA, NOTCH3, SEMA7A, SEMA3C, DHH, TSHZ2, LRP4, TULP3, ATOH8, NNAT, MSX1, RELT, PDLIM7, JAG2, ZFP831, NTRK2, SEMA4A, SEMA4D, NDE1, FZD6, VEGFC, SEMA4F, TDRD7, SORL1, GGN, ICK, TTLL7, BMP2, KIF26B, SNAI2, ACKR3, CSPG4, PICALM
calcium ion transport	0.005248	2.323208963	RYR1, CALCRL, SLC24A3, RAMP3, PRKCB, CAV1, TRPV2, PKD2, CORO1A, CACNA1I, TRPV4, MYB, GJA4, CCL3, CLCA1
glycolytic process	0.005405	4.246309716	PFKL, TPI1, PKM, BPGM, ALDOA, ENO3, HK1
negative regulation of axon extension involved in axon guidance	0.005836	5.039576366	SEMA6B, SEMA4A, SEMA4D, SEMA3C, SEMA4F, SEMA3F
positive regulation of cell proliferation	0.006123	1.571380823	PTGER4, SHC4, CSF1R, CALCRL, NLGN2, LEF1, IRS2, HTR2A, PTGS2, PLAC8, PAK1, EDNRB, EPCAM, CXCR3, RAC2, PTK2B, MARK4, NTRK2, EDN1, ST8SIA1, VEGFC, NCCRP1, AGT, PGF, ACER2, IL4, TIAM1, HCK, CXCL12, MYCN, FASL, CLCF1, ACER3, MZB1, HCLS1, PTPN6, KRAS, FGFR3, FGFR2
negative regulation of inflammatory response	0.006293	2.761147204	PTGER4, MIR147, CALCRL, CNR2, ADORA2A, ADIPOQ, ADORA1, TNFAIP3, WFDC1, TNFRSF1B, UACA
positive regulation of T cell activation	0.006414	6.422989486	CCDC88B, CD4, LCK, CD3E, CORO1A
toll-like receptor signaling pathway	0.006414	6.422989486	TLR1, CD86, UNC93B1, CD180, TLR9
positive regulation of immunoglobulin production	0.006508	9.705850778	SASH3, IL4, CLCF1, CD37
negative regulation of cytokine-mediated signaling pathway	0.006508	9.705850778	SIGIRR, IL1RN, PTPRC, CAV1
myeloid cell differentiation	0.006898	4.852925389	CEBPA, GFI1, MYB, STAP1, IRF8, GATA1
response to drug	0.007186	1.739322816	RET, CD86, ATP1A3, WFDC1, AK4, HTR2A, PTGS2, ENO3, AQP1, NPPB, CCL3, PTK2B, TIMP4, SNCA, TGIF1, ARG1, ITGA3, PRKCB, IGF2, VEGFC, NFATC2, PGF, IL4, ADORA2A, LOX, LCK, CYP1A1
cellular response to lipopolysaccharide	0.00759	1.985287659	CD86, CX3CR1, CDK19, NOS2, ARG1, GFI1, CD180, TNFAIP3, TNFRSF1B, MIR147, EDNRB, KLRK1, PLSCR3, TNIP3, TLR9, PDE4B, IL12B, STAP1, IRF8
platelet activation	0.008064	3.919670507	ENTPD1, SYK, VWF, FZD6, RASGRP2, VAV1, CX3CL1
oxygen transport	0.008984	8.7352657	HBB-B2, HBB-B1, HBB-BT, HBB-BS
phagocytosis	0.009082	3.359717577	HCK, PLD4, IRF8, ITGAL, CORO1A, VAV1, MYO1G, GULP1
cell chemotaxis	0.009086	2.799764648	CCL22, CXCL12, BIN2, SAA3, GPR183, LEF1, CCL4, CCL3, EPHB1, CX3CL1
cellular response to mechanical stimulus	0.009086	2.799764648	PTGER4, NPPB, NOS3, BNIP3, CAV1, FAS, PTGS2, MAP3K14, AGT, AQP1
patterning of blood vessels	0.009128	3.821678744	EDN1, CXCL12, FLT1, COL4A1, LEF1, CXCR4, STK4
lymph node development	0.010879	4.36763285	RIPK3, CXCR5, CCR7, LTB, IKZF1, IL7R
positive regulation of establishment of protein localization to plasma membrane	0.010879	4.36763285	VIL1, RAMP3, ITGA3, KIF5B, EZR, NKD2
B cell differentiation	0.011525	2.696069661	CD79B, CD79A, CR2, PTPRC, BCL6, CLCF1, MYB, PLCG2, IKZF1, RAG1
extrinsic apoptotic signaling pathway in absence of ligand	0.011553	3.639694042	IL4, BCL2L11, UNC5B, FAS, BCL2A1D, BCL2A1A, BCL2A1B

neutrophil activation involved in immune response	0.011787	16.37862319	ZAP70, FCER1G, SYK
positive regulation of protein localization to early endosome	0.011787	16.37862319	RDX, EZR, SORL1
maintenance of blood-brain barrier	0.011787	16.37862319	BDKRB2, PTGS2, ABCB1A
mast cell degranulation	0.011938	7.941150637	LAT2, MILR1, RASGRP1, LAT
regulation of B cell differentiation	0.011938	7.941150637	PTPRC, PTPN6, IKZF3, ZFP36L2
urea cycle	0.011938	7.941150637	CEBPA, ARG1, GM5424, ASS1
superoxide anion generation	0.011938	7.941150637	EDN1, NCF1, NCF2, PRG3
cellular response to interferon-gamma	0.012114	2.890345269	CIITA, EDN1, CCL22, NOS2, CCL4, CCL3, IL12B, AIF1, CX3CL1
T cell homeostasis	0.012494	4.226741468	GPR174, BCL2L11, RIPK3, FAS, CORO1A, RAG1
epithelial to mesenchymal transition	0.012494	4.226741468	BMP2, LEF1, SNAI2, MSX1, FGFR2, LOXL2
MAPK cascade	0.013171	2.848456207	RET, DOK2, PLVAP, TAOK3, CAV1, CCL3, PTK2B, FGFR3, ZFP36L2
neutrophil chemotaxis	0.013171	2.848456207	CCL22, FCER1G, SYK, CCL4, PDE4B, CCL3, VAV1, CXCL15, CX3CL1
actin cytoskeleton organization	0.013379	2.153058447	TMOD3, CORO1A, SPTA1, PAK1, FMNL1, NUAK2, BCL6, RHOJ, RAC2, CCR7, KRAS, DOCK2, PDLIM7, PHACTR4
positive regulation of natural killer cell mediated cytotoxicity	0.013955	5.199562917	KLRK1, CD226, SLAMF6, RASGRP1, VAV1
bone morphogenesis	0.014264	4.094655797	CHSY1, SP5, MSX1, FGFR3, DHRS3, FGFR2
Fc-epsilon receptor signaling pathway	0.015383	7.279388084	ITK, TEC, BTK, RASGRP1
positive thymic T cell selection	0.015383	7.279388084	ZAP70, PTPRC, DOCK2, CD3D
positive regulation of T cell differentiation in thymus	0.015383	7.279388084	VNN1, TESPA1, IL7R, RASGRP1
regulation of GTPase activity	0.015484	2.768218004	TIAM1, NTRK2, BCL6, RASA3, RDX, RASAL3, ADAP1, RASGRP1, VAV1
vasodilation	0.016453	4.963219148	EDNRB, ADORA2A, KCNJ8, BDKRB2, AGT
myeloid dendritic cell differentiation	0.016453	4.963219148	CD86, IL4, SPI1, IRF4, BATF
negative regulation of transcription from RNA polymerase II promoter	0.016862	1.407947489	NOTCH3, CEBPA, CIITA, SPI1, SATB1, SLFN1, GFI1, LEF1, TCF7, IKZF1, GATA1, EDNRB, SOX17, MXI1, MYB, HEY2, MSX1, EOMES, TGIF1, EDN1, SEMA4D, PEG3, CAV1, FST, NFATC2, HFE2, PAX5, HIC1, BMP2, MAF, BCL6, FASL, TRPV4, PKIA, CRY1, TIMELESS, REL, IRF8, SNAI2, HCLS1, SIK1, TRIB3, EZR, MET, FGFR3, RCOR2, FGFR2
calcium-mediated signaling	0.017698	3.323198908	LAT2, EDN1, CXCR3, CCL3, CXCR4, PPP1R9A, LAT
negative regulation of canonical Wnt signaling pathway	0.018261	2.355096145	BMP2, SOX17, CAV1, FZD6, LEF1, PTPRO, LRP4, SNAI2, STK4, NKD2, CTHRC1
brown fat cell differentiation	0.018292	3.853793691	PLAC8, CEBPA, BNIP3, ADIPOQ, SLC2A4, PTGS2
homeostasis of number of cells	0.018292	3.853793691	RASSF2, MYB, PIK3CD, CCR7, IL7R, LAT
regulation of eIF2 alpha phosphorylation by heme	0.019051	13.10289855	HBB-B1, HBB-BT, HBB-BS
T cell receptor V(D)J recombination	0.019051	13.10289855	BCL11B, TCF7, LEF1
negative regulation of macrophage chemotaxis	0.019051	13.10289855	SLAMF8, STAP1, HC
B cell proliferation involved in immune response	0.019051	13.10289855	CD180, PLCL2, GAPT
positive regulation of odontogenesis	0.019051	13.10289855	BMP2, EDN1, CD34
endocytosis	0.019247	1.930445459	SH3GL3, SH3KBP1, CAV1, STAB2, LRP4, SNX30, CD209A, SORL1, DNM3, PSTPIP1, FCHO1, NOSTRIN, UNC119, RIN1, PACSIN1, PICALM
axon guidance	0.019273	2.05190805	CSF1R, SEMA3C, LAMA1, UNC5B, PTPRO, CRMP1, SEMA4F, ANK3, SEMA3F, CDH4, KIF26B, KIF5B, EPHB1, SCN1B
negative regulation of G-protein coupled receptor protein signaling pathway	0.01933	6.719435154	RGS4, RGS5, CRY1, KLK14
regulation of cell-cell adhesion	0.01933	6.719435154	EPHA7, PODXL, LEF1, ITGAL
positive regulation of neuron differentiation	0.019427	2.332231134	SPAG9, TGIF1, GPRC5B, BMP2, BRINP1, CXCL12, BCL6, BNIP2, KCTD11, GDPD5, ETV5
regulation of apoptotic process	0.020667	1.86557182	TRAF1, TNFRSF1B, IKZF3, AGT, BMP2, RASSF2, BCL2L11, SELL, LCK, RASSF5, RASSF6, TRIM24, SNAI2, FAS, INHA, BCL2A1A, RELT
regulation of inflammatory response	0.020846	2.864021541	IL4, SEMA7A, PGLYRP2, BCL6, CLCF1, TLR9, AGT, MGLL
brain development	0.021023	1.819847021	EOMES, NDRG4, EPHA7, SHROOM2, TULP3, CXCR4, IRS2, AK4, CXCL12, BCL2L11, COL4A1, SNPH, B3GNT5, STMN1, NNAT, POMK, FAS, MET
cardiac muscle contraction	0.021482	3.184732287	GAA, TNNI1, ATP1A3, CXCR4, SCN3B, MET, SCN1B
T cell proliferation	0.022242	4.549617552	PTPRC, CXCL12, EBI3, CXCR4, DOCK2
actin filament polymerization	0.022242	4.549617552	VIL1, WAS, COBL, HCLS1, AIF1
positive regulation of synaptic transmission, glutamatergic	0.022242	4.549617552	NTRK2, NLGN2, ADORA2A, PTK2B, PTGS2
regulation of actin filament polymerization	0.022242	4.549617552	ELN, HCLS1, CLEC2I, PPP1R9A, CORO1A
regulation of cell size	0.022242	4.549617552	RAP1GAP2, KEL, RDX, IL7R, VAV1
regulation of blood pressure	0.022605	2.817827645	EDN1, EDNRB, CYP4F18, ACE, NPR3, PTGS2, CD34, AGT
branching morphogenesis of an epithelial tube	0.023005	3.639694042	MMP14, PAK1, MYCN, WNT9B, TIMELESS, MET
negative regulation of potassium ion transport	0.02378	6.2394755	PLCB4, NOS3, PTK2B, HTR2A
macrophage chemotaxis	0.02378	6.2394755	CX3CR1, EDNRB, CCL3, CX3CL1
drug transmembrane transport	0.02378	6.2394755	ATP8B1, ABCB4, ABCB1A, ABCG2
striated muscle cell differentiation	0.02378	6.2394755	SPAG9, IGF2, BNIP2, KRAS
odontogenesis of dentin-containing tooth	0.024463	2.773100222	JAG2, BMP2, BCL2L11, BCL11B, FST, LEF1, LRP4, MSX1
positive regulation of neuron projection development	0.024501	2.057218371	RET, NTRK2, NDRG4, ITGA3, STMN2, PPP1R9A, TIAM1, GPRC5B, ADAMTS1, P2RY2, PTK2B, MET, SCN1B
vascular endothelial growth factor receptor signaling pathway	0.025546	4.36763285	FLT1, VEGFC, PTK2B, VAV1, PGF
release of sequestered calcium ion into cytosol	0.02563	3.541323933	RYR1, PTPRC, LCK, PLCG2, HTR2A, PKD2
positive regulation of phosphatidylinositol 3-kinase signaling	0.026423	2.729770531	NTRK2, FLT1, SEMA4D, UNC5B, CD28, HCLS1, PTPN6, AGT
positive regulation of protein kinase activity	0.026423	2.729770531	CD4, GPRC5B, RASSF2, ADIPOQ, PTK2B, LCP2, PPP1R9A, LAT
lipid catabolic process	0.027588	2.203851438	LIPE, ACAP1, PLCB4, ADORA1, PLCG2, PLD4, PLBD1, PLIN1, PAFAH2, MGLL, PLA1A
T cell chemotaxis	0.027718	10.91908213	CXCR3, GPR183, CCL3
renal water absorption	0.027718	10.91908213	HYAL2, AQP3, AQP1
positive regulation of gamma-delta T cell differentiation	0.027718	10.91908213	PTPRC, SYK, LCK
aging	0.027752	1.89348245	CD86, CIITA, ARG1, HTR2A, PAX5, RETN, PPP1R9A, TNFRSF1B, ENO3, AGT, EDNRB, CYP1A1, PCK1, CTSC, SNCA
homeostasis of number of cells within a tissue	0.028439	3.448131198	SASH3, CD7, KRAS, TEX15, CORO1A, GATA1
neuromuscular junction development	0.028439	3.448131198	NTRK2, PAK1, COL4A1, DOK7, ANK3, FGFR2
positive regulation of cell adhesion mediated by integrin	0.028736	5.823510467	RET, ZAP70, SYK, PTPN6
activation of phospholipase C activity	0.028736	5.823510467	ITK, TXK, HTR2A, AGT
regulation of actin cytoskeleton reorganization	0.028736	5.823510467	CSF1R, GMFG, ARHGDIB, PTK2B
detection of temperature stimulus involved in sensory perception of pain	0.028736	5.823510467	LXN, ADORA1, HTR2A, EPHB1
B cell homeostasis	0.029129	4.199646971	BCL2L11, PIK3CD, TNFRSF13C, BCL2A1A, GAPT
ERK1 and ERK2 cascade	0.029129	4.199646971	PTGER4, FGFR3, ZFP36L2, CCR3, AGT
negative chemotaxis	0.029129	4.199646971	SEMA4A, SEMA4D, SEMA3C, SEMA4F, SEMA3F
cellular response to tumor necrosis factor	0.029155	2.183816425	CEBPA, EDN1, CCL22, HYAL2, CCL4, CHIL1, CCL3, SLC2A4, PCK1, ZFP36L2, CX3CL1
cellular response to interleukin-1	0.029598	2.456793478	EDN1, CCL22, SAA3, HYAL2, CCL4, CHIL1, CCL3, PCK1, CX3CL1
negative regulation of cell proliferation	0.030328	1.535495924	CSF1R, CEBPA, SLFN1, PTGS2, STK4, NDRG1, GATA1, RASSF5, ADORA1, TRIM24, BDKRB2, PTK2B, CD37, MSX1, TGIF1, BCL11B, NOS3, CAV1, TRPV2, VEGFC, DHCR24, AGT, BMP2, ADORA2A, BCL6, FGFR3, FGFR2
positive regulation of catalytic activity	0.031435	3.359717577	APH1A, CAV1, IGF2, WNT9B, AGT, PHACTR4

cell differentiation	0.031963	1.343887031	HEMGN, BLK, NOTCH3, SEMA7A, EHF, FLT1, SEMA3C, DAPL1, TCF23, LRP4, PIK3CD, RASGRP1, FCRLA, KLRK1, MECOM, ANPEP, ATOH8, STMN1, THSD7A, PDLIM7, JAG2, SEMA6B, EOMES, NTRK2, SEMA4A, EGFL6, YES1, SEMA4D, NDE1, TNK1, VEGFC, SEMA4F, TDRD7, PAX5, TEX15, ETV5, PGF, BATF, GGN, TTLL7, HCK, BMP2, TEC, PTPN6, KCTD11, SIK1, CSPG4, FGFR3
negative regulation of cysteine-type endopeptidase activity involved in apoptotic process	0.032937	2.607542	ADORA2A, LAMP3, LEF1, DHCR24, BCL2A1D, AQP1, SNCA, RAG1
positive regulation of endothelial cell proliferation	0.032937	2.607542	BMP2, CXCL12, ARG1, CAV1, VEGFC, FGFR3, CCR3, PGF
cellular response to interleukin-4	0.032995	4.044104491	ARG1, TCF7, LEF1, KEAP1, CORO1A
lipid storage	0.032995	4.044104491	GM2A, CAV1, HEXA, CRY1, B4GALNT1
barbed-end actin filament capping	0.034194	5.459541063	VIL1, RDX, CAPG, ADD2
negative regulation of T cell proliferation	0.034622	3.275724638	SPN, ZC3H12D, SLFN1, BTLA, CTLA4, PTPN6
monocyte chemotaxis	0.034622	3.275724638	CCL22, FLT1, PTPRO, CCL4, CCL3, CX3CL1
cytokine-mediated signaling pathway	0.035783	1.944494077	CX3CR1, CSF1R, CEBPA, IL1RN, IFNGR1, ASPN, CX3CL1, IL2RB, IL3RA, STAT4, PTPN6, KRAS, IRF5
intrinsic apoptotic signaling pathway in response to DNA damage	0.035995	2.830873144	BCL2L11, BCL2A1D, TNFRSF1B, BCL2A1A, UACA, BCL2A1B, HIC1
positive regulation of neutrophil chemotaxis	0.037147	3.899672188	EDN1, SELL, RAC2, CCR7, CXCL15
protein homooligomerization	0.037206	1.77365801	SPAG9, ENTPD1, DGKD, VWF, RIPK3, CAV1, ADIPOQ, PRF1, AK3, EHD4, STOM, FAS, KCTD14, AQP11, KCTD11, KCTD17
positive regulation of natural killer cell mediated cytotoxicity directed against tumor cell target	0.037643	9.359213251	KLRK1, IL12B, CD226
cellular response to osmotic stress	0.037643	9.359213251	TRPV4, SLC2A4, PKD2
ossification involved in bone maturation	0.037643	9.359213251	RYR1, SEMA4D, THBS3
regulation of sensory perception of pain	0.038001	3.195828915	EDN1, EDNRB, ADORA1, CCL3, CTSS, MGLL
positive regulation of multicellular organism growth	0.038001	3.195828915	VIL1, FOXS1, IGF2, IKZF1, EZR, AGT
retina development in camera-type eye	0.038058	2.339803313	RET, TGIF1, IL4, NTRK2, ACHE, LAMA1, LAMC3, IKZF1, SLC4A5
positive regulation of cytosolic calcium ion concentration	0.039099	1.91821713	PTGER4, EDN1, HTR2A, PKD2, AGT, EDNRB, TRPV4, P2RY2, CXCR3, CCL3, PLCG2, BDKRB2, PTK2B
cellular calcium ion homeostasis	0.039438	2.183816425	RYR1, KEL, SLC24A3, DMPK, PRKCB, TRPV4, CAV1, CCL3, HTR2A, PKD2
cellular defense response	0.040147	5.138391589	NCF1, CD19, PTK2B, LAT
sprouting angiogenesis	0.041584	3.765200733	FLT1, LEF1, PTK2B, PGF, LOXL2
negative regulation of tumor necrosis factor production	0.045346	3.047185709	PTGER4, ADIPOQ, TNFAIP3, PTPN22, CD34, GPR18
positive regulation of tyrosine phosphorylation of Stat3 protein	0.046308	3.639694042	PTGER4, CSF1R, CLCF1, IL12B, FGFR3
positive regulation of B cell differentiation	0.046585	4.852925389	ZAP70, MMP14, SYK, IKZF1
positive regulation of cardiac muscle hypertrophy	0.046585	4.852925389	EDN1, PARP1, AGT, PDE9A
induction of positive chemotaxis	0.046585	4.852925389	CXCL12, VEGFC, IL16, PGF
actin filament capping	0.046585	4.852925389	SPTA1, VIL1, RDX, CAPG
positive regulation of nitric-oxide synthase activity	0.046585	4.852925389	NPR3, PTK2B, FCER2A, KRAS
neuromuscular process controlling balance	0.048501	2.635640513	ALDH1A3, CLIC5, FOXS1, NLGN2, GM2A, GAA, HEXA
cytoskeleton organization	0.048642	2.099823486	VIL1, BLK, HCK, FMNL1, SH3KBP1, CCL3, LSP1, DOCK2, PACSIN1, MARK1
antigen processing and presentation of endogenous peptide antigen via MHC class Ib via ER pathway, TAP-dependent	0.048694	8.189311594	H2-T23, ABCB4, ABCB1A
positive regulation of type I hypersensitivity	0.048694	8.189311594	FCER1G, BTK, FCER1A
negative regulation of smooth muscle contraction	0.048694	8.189311594	CALCRL, NCF1, PTGS2
positive regulation of interleukin-13 production	0.048694	8.189311594	IL4, H2-T23, IL33
positive regulation of granulocyte macrophage colony-stimulating factor production	0.048694	8.189311594	TLR9, IL12B, RASGRP1
regulation of NIK/NF-kappaB signaling	0.048694	8.189311594	RASSF2, PTPN22, UACA
response to yeast	0.048694	8.189311594	CD86, NCF1, PTX3
interferon-gamma production	0.048694	8.189311594	EOMES, ITK, TXK
negative regulation of platelet-derived growth factor receptor signaling pathway	0.048694	8.189311594	NDRG4, ADIPOQ, SNCA
negative regulation of leukocyte apoptotic process	0.048694	8.189311594	CXCL12, HCLS1, CCR7
carbon dioxide transport	0.048694	8.189311594	CAR14, RHAG, AQP1
negative regulation of Notch signaling pathway	0.051318	3.522284557	MMP14, BCL6, HEY2, NUMB, GDPD5
positive regulation of cytosolic calcium ion concentration involved in phospholipase C-activating G-protein coupled signaling pathway	0.051318	3.522284557	GPR17, P2RY10, EDN1, GPR65, GPR18
regulation of neuron differentiation	0.051318	3.522284557	EOMES, BCL11B, NUMB, NREP, S1PR5
programmed cell death	0.051318	3.522284557	PKM, RIPK3, MLKL, STK17B, RELT
sensory perception of sound	0.051876	1.898970804	JAG2, SLC9A3R2, CLIC5, MARVELD2, ATP8B1, SCN8A, HEXA, SNAI2, FAM65B, MBP, TPRN, FAM107B
positive regulation of peptidyl-serine phosphorylation	0.052065	2.360882622	NTRK2, PAK1, CAV1, HCLS1, STK4, MET, AGT, SNCA
regulation of blood coagulation	0.053499	4.597508263	CAV1, FAM46A, STAB2, CD209A
positive regulation of myoblast fusion	0.053499	4.597508263	CD53, IL4, CXCL12, NFATC2
cell-substrate adhesion	0.053499	4.597508263	VWF, CORO1A, EPHB1, MYO1G
regulation of ossification	0.053499	4.597508263	PTGER4, RSAD2, FGFR3, DHRS3
response to glucocorticoid	0.055266	2.329404187	IL1RN, ABCA3, ADIPOQ, FAS, KRAS, PTGS2, AIF1, ASS1
cellular response to drug	0.055266	2.329404187	ACER2, EDN1, NOS2, ADIPOQ, CCL4, PDE4B, SLAMF8, CD69
positive regulation of extrinsic apoptotic signaling pathway	0.056611	3.412213164	PTPRC, HYAL2, CAV1, FAS, AGT
positive regulation of apoptotic process	0.057147	1.49933665	EPHA7, CDK19, ACE, NOS3, BNIP3, GZMA, STK4, PTGS2, UACA, ALDH1A3, BMP2, RASSF2, BCL2L11, KLRK1, BCL6, FASL, RASSF6, FAS, CTLA4, IRF5, BCL2A1D, BCL2A1A, BCL2A1B
lung development	0.058044	1.937256506	CEBPA, MMP14, MYCN, LOX, ITGA3, ARG1, NOS3, TIMELESS, CHIL1, AARD, FGFR2
Notch signaling pathway	0.060638	1.921758454	CFD, JAG2, APH1A, NOTCH3, CEBPA, BMP2, HEY2, NUMB, SNAI2, GZMB, TIMP4
toll-like receptor 3 signaling pathway	0.060747	7.279388084	CD86, UNC93B1, RFTN1
glomerular filtration	0.060747	7.279388084	MCAM, CD34, AQP1
regulation of phagocytosis	0.060747	7.279388084	BLK, HCK, SYK
lymphocyte differentiation	0.060747	7.279388084	SPI1, LY6D, IKZF1
negative regulation of heterotypic cell-cell adhesion	0.060747	7.279388084	IL1RN, ADIPOQ, TNFAIP3
negative regulation of gene expression	0.061763	1.565755173	TGIF1, ACE, NOS2, SLC35C2, KEAP1, PTPN22, CD3E, AIF1, BMP2, MYCN, HEY2, CD28, CCL3, REL, MSX1OS, FGFR3, CD34, MET, PICALM
regulation of Rho protein signal transduction	0.062029	2.268900182	FGD3, TIAM1, ARHGEF9, ARHGEF25, ARHGDIB, EPS8L1, VAV1, FGD2
negative regulation of extrinsic apoptotic signaling pathway in absence of ligand	0.062186	3.308812765	CX3CR1, UNC5B, SNAI2, GATA1, CX3CL1
Ras protein signal transduction	0.062404	2.787850755	DOK2, DOK3, DHCR24, KRAS, RASGRP1, LAT
protein localization to plasma membrane	0.063386	2.46559919	RAMP3, FCER1G, ADIPOQ, ANK3, SCN3B, SKAP1, FYB

positive regulation of transcription from RNA polymerase II promoter	0.064099	1.251030515	EHF, CIITA, SPI1, FHL5, HIF3A, IKZF1, PKD2, CD3D, IKZF3, MECOM, SOX17, HYAL2, CREB3L2, MYB, HEY2, EOMES, EDN1, PARP1, PEG3, HFE2, PAX5, ETV4, ETV5, MYCN, MAF, IRF4, TLR9, HCLS1, NCOA7, IRF5, MET, SKAP1, CEBPA, NOTCH3, BEX2, TXK, LEF1, GATA1, PLAC8, EPCAM, CCL3, STAT4, MSX1, ZBTB7C, IL33, YES1, BCL11B, IGF2, NFATC2, POU2F2, BATF, IL4, BMP2, CD28, REL, CRLF3, FGFR2
nervous system development	0.064778	1.448154128	RET, SEMA7A, NRN1, SEMA3C, CRMP1, CXCR4, GDPD5, ATOH8, STMN1, SCN3B, MARK4, EPHB1, PHACTR4, SEMA6B, NTRK2, SEMA4A, EPHA7, SEMA4D, NDE1, FZD6, SEMA4F, PAX5, TTLL7, NUMB, KCTD11
erythrocyte differentiation	0.067162	2.729770531	ALAS2, SPI1, HCLS1, IKZF1, TRIM10, GATA1
branching involved in ureteric bud morphogenesis	0.067162	2.729770531	BMP2, WNT9B, TIMELESS, PKD2, AGT, PGF
response to mechanical stimulus	0.067483	2.426462695	MMP14, CXCL12, LCK, CAV1, CHIL1, PTK2B, RETN
stem cell differentiation	0.068038	3.211494743	SHC4, SOX17, EPCAM, MSX1, ETV4
negative regulation of ossification	0.068692	4.159650334	CHSY1, P2RY2, LRP4, PTK2B
negative regulation of smoothened signaling pathway	0.068692	4.159650334	TULP3, KCTD11, CD3E, FGFR3
response to osmotic stress	0.068692	4.159650334	TRPV4, SLC2A1, FMO1, PTK2B
liver development	0.069173	2.068878719	CEBPA, PKM, VWF, ARG1, COBL, AK4, KRAS, PKD2, MET
protein localization	0.069275	2.211459671	CLIC5, BCL6, KIF26B, CAV1, KIF5B, LRP4, DHCR24, GGN
positive regulation of GTPase activity	0.071659	1.79491761	RGS4, CCL22, SEMA4D, RASGEF1B, RGS1, CCL4, CCL3, CCR7, RASGRP2, RASGRP1, VAV1, CX3CL1
positive regulation of fat cell differentiation	0.072116	2.674060929	CEBPA, BMP2, SNAI2, HTR2A, STK4, ZBTB7C
positive regulation of smooth muscle cell proliferation	0.073079	2.183816425	NOTCH3, EDN1, CALCRL, RETN, PTGS2, AIF1, FGFR2, CX3CL1
positive regulation of chemokine secretion	0.073688	6.551449275	CSF1R, IL33, HC
alpha-beta T cell differentiation	0.073688	6.551449275	BCL11B, TCF7, LEF1
artery smooth muscle contraction	0.073688	6.551449275	EDN1, HTR2A, AGT
positive regulation of isotype switching to IgG isotypes	0.073688	6.551449275	IL4, PTPRC, CD28
bone mineralization	0.074164	3.11973775	BMP2, PTGS2, FGFR3, ASPN, FGFR2
actin filament bundle assembly	0.074164	3.11973775	CALD1, SHROOM2, EZR, AIF1, ADD2
response to bacterium	0.074164	3.11973775	VIL1, NCF1, MECOM, CAV1, IRF8
kidney development	0.074725	1.847844667	ACE, BCL2L11, KCNJ8, ADAMTS1, WNT9B, TIMELESS, LRP4, AQP11, PKD2, PCSK5, AGT
bicarbonate transport	0.07694	3.970575318	SLC4A1, SLC26A9, SLC26A10, SLC4A5
regulation of sodium ion transport	0.07694	3.970575318	DMPK, NOS3, SIK1, SCN1B
positive regulation of T cell differentiation	0.07694	3.970575318	IL4, ZAP70, PTPRC, RAG1
cell morphogenesis	0.077005	2.156855729	BCL6, UNC93B1, LST1, PTPRO, SHROOM2, MSX1, IL7R, STK4
extracellular matrix organization	0.077506	1.915628443	ERO1LB, EGFL6, COL4A2, LAMA1, LAMC3, COL4A1, ELN, ABI3BP, FBLN1, AGT
phosphatidylinositol-mediated signaling	0.080559	3.033078368	CSF1R, NPR3, PIK3CD, IRS2, EZR
excitatory postsynaptic potential	0.080559	3.033078368	ADORA2A, ADORA1, PPP1R9A, MET, SNCA
negative regulation of ERK1 and ERK2 cascade	0.080675	2.316168936	PTPRR, CAV1, ADIPOQ, TBC1D10C, TIMP3, FBLN1, EZR
peptidyl-serine phosphorylation	0.084083	1.806163961	MAP4K1, SBK1, SYK, DMPK, PRKCB, PKN3, NEK6, VRK1, STK4, PDK1, HK1
memory	0.085219	2.104883301	CX3CR1, IL1RN, ITGA3, IGF2, ATP1A3, HTR2A, PTGS2, RIN1
actin filament organization	0.085219	2.104883301	TRPV4, TMOD3, WAS, RAC2, PTK2B, PPP1R9A, CORO1A, PACSIN1
signal transduction involved in regulation of gene expression	0.0856	3.797941609	PARP1, SOX17, EPCAM, MSX1
natural killer cell activation	0.08741	5.955862978	KLRK1, IL2RB, IL12B
regulation of T cell proliferation	0.08741	5.955862978	RAC2, CLEC2I, CD209A
phospholipid translocation	0.08741	5.955862978	ATP8B1, ABCB4, ABCB1A
renal system process	0.08741	5.955862978	FAS, AGT, SLC4A5
positive regulation of interferon-gamma secretion	0.08741	5.955862978	CD2, PTPN22, RASGRP1
negative regulation of microtubule polymerization	0.08741	5.955862978	STMN1, STMN2, SNCA
macrophage activation involved in immune response	0.08741	5.955862978	IL33, ZAP70, SYK
positive regulation of cellular protein catabolic process	0.08741	5.955862978	RDX, TNFAIP3, EZR
heterophilic cell-cell adhesion via plasma membrane cell adhesion molecules	0.088138	2.519788183	CDH4, CD6, MCAM, CD226, ITGAL, CD200
interleukin-4 production	0.089383	21.83816425	ITK, TXK
lymphocyte aggregation	0.089383	21.83816425	STK10, RAC2
negative regulation of long-term synaptic potentiation	0.089383	21.83816425	CX3CR1, PPP1R9A
positive regulation of immunological synapse formation	0.089383	21.83816425	CCR7, CLEC2I
regulation of the force of heart contraction by chemical signal	0.089383	21.83816425	NOS3, CAV1
negative regulation of metalloendopeptidase activity involved in amyloid precursor protein catabolic process	0.089383	21.83816425	SORL1, PICALM
nitric oxide transport	0.089383	21.83816425	EDN1, AQP1
regulation of endocannabinoid signaling pathway	0.089383	21.83816425	ABHD6, MGLL
negative regulation of Wnt signaling pathway involved in heart development	0.089383	21.83816425	BMP2, SOX17
negative regulation of antigen processing and presentation of peptide antigen via MHC class II	0.089383	21.83816425	H2-OB, H2-OA
CD8-positive, gamma-delta intraepithelial T cell differentiation	0.089383	21.83816425	CCR9, GPR18
S-adenosylmethioninamine biosynthetic process	0.089383	21.83816425	AMD2, AMD1
leukocyte mediated cytotoxicity	0.089383	21.83816425	NCF1, STXBP2
positive regulation of toll-like receptor 7 signaling pathway	0.089383	21.83816425	RSAD2, PTPN22
leukocyte adhesive activation	0.089383	21.83816425	SELPLG, CX3CL1
response to virus	0.089506	2.079825167	RSAD2, OAS2, HYAL2, MX1, STMN1, CYP1A1, TLR9, KLRA8
glucose metabolic process	0.090213	2.24804632	TPI1, PKM, ADIPOQ, IGF2, PCK1, PGM1, PDK1
carbohydrate metabolic process	0.090858	1.590157591	GAA, HEXA, IGF2, HK1, GLB1L2, PPP1R2, PFKL, LDHA, HYAL2, CHST10, CHIL1, CHIL3, CHST2, PGM1, PDK1
leukocyte cell-cell adhesion	0.094651	3.639694042	PTPRC, SYK, ITGAL, EZR
collagen catabolic process	0.094651	3.639694042	MMP14, ADAM15, CTSK, CTSS
positive regulation of activated T cell proliferation	0.094651	3.639694042	CD86, IL4, IGF2, IL12B
cell-cell signaling	0.099405	1.908189109	GJC1, EDN1, DHH, GJA4, WNT9B, CCL3, FGFR3, GATA1, FGFR2
cell migration	0.099621	1.600703139	FLT1, SH3KBP1, SHROOM2, NFATC2, CXCR4, CORO1A, SORL1, TIAM1, PSTPIP1, MMP14, PODXL, IL12B, SNAI2, CTHRC1
defense response to bacterium	0.099621	1.600703139	FCER1G, SYK, NOS2, NCF1, LYZ2, LYZ1, PRG2, STAB2, SPN, TLR1, PLAC8, ADAMTS4, SLAMF8, IRF8
