# Supplementary material for: Endothelial senescence mediates hypoxia-induced vascular remodeling by modulating PDGFB expression
Source: Front Med (Lausanne). 2022 Sep 20;9:908639. doi: 10.3389/fmed.2022.908639 (PMC9530050; doi:10.3389/fmed.2022.908639)
Supplement: Supplementary file 8 [file Data_Sheet_5.PDF]

Biological Processes	PValue	Fold Enrichment	Genes
immune system process	3.16E-17	3.478141043	CD86, ITK, H2-T23, PGLYRP2, NRROS, LST1, PIK3CD, PTPN22, LRMP, TNFRSF13C, IFI30, CD3E, PSTPIP1, LAMP3, CTLA4, EOMES, CR2, RSAD2, SYK, PRKCB, CD180, CD8B1, TLR1, LAT2, HCK, ZAP70, OAS2, CD8A, TLR12, BTK, TLR9, IRF5, SKAP1, CFD, CSF1R, UNC93B1, TXK, LY9, CD79B, CD79A, KLRK1, BTLA, PTK2B, SLAMF7, SLAMF6, CD300LG, CD55, SEMA4A, MX1, PRG2, LY86, CD4, TEC, BPIFB1, THEMIS2, BCL6, GPR183, CD7, HC, LAT, MYO1G
adaptive immune response	4.98E-16	5.498818337	CD86, H2-T23, ITK, UNC93B1, TXK, PIK3CD, TNFRSF13C, LY9, CTSS, CD79B, **CD79A**, KLRK1, LAMP3, BTLA, SLAMF7, PTK2B, CTLA4, SLAMF6, RAG1, EOMES, SEMA4A, **SYK**, PRKCB, CD8B1, LAT2, ZAP70, CD4, TEC, CD8A, GPR183, CD7, BTK, SKAP1, LAT, MYO1G
immune response	9.60E-13	3.532644217	MCPT8, NRROS, MCPT4, LST1, WAS, CXCR5, CTSW, FYB, CXCL15, CX3CL1, CTSS, H2-DMB2, CCL4, CCL3, H2-OB, CCR9, BLNK, CTLA4, CCR7, H2-OA, TINAG, RELT, H2-EB2, CCL22, PRG2, PRG3, TNFRSF1B, VAV1, TLR1, IL4, ZAP70, CD4, CXCL12, FASL, OAS2, CD28, TLR9, TLR12, FAS, IRF8, LCP2, LTB, MAP3K14, LAT
transmembrane receptor protein tyrosine kinase signaling pathway	2.08E-12	5.677922705	RET, PTPRT, SHC4, **BLK**, ITK, CSF1R, FLT1, TXK, STAP1, EPHB1, NTRK2, EPHA7, YES1, **SYK**, TNK1, PILRA, ZAP70, HCK, DOK2, TEC, LCK, BTK, LCP2, CSPG4, MET, LAT
inflammatory response	6.11E-11	2.983702674	CSF1R, SEMA7A, CIITA, LXN, NRROS, NCF1, PIK3CD, TNFAIP3, PTGS2, AIF1, CXCL15, CX3CL1, NPPB, PSTPIP1, CNR2, MECOM, CXCR3, CCL4, CCL3, CCR7, RELT, CCR3, CCL22, NOS2, CD180, LY86, TNFRSF1B, TLR1, ZAP70, HCK, BMP2, VNN1, CXCL12, THEMIS2, BCL6, TLR9, CHIL1, REL, TLR12, FAS, ACKR2, CHIL3, CSPG4, HC, NFKBID, LAT, CHST2
cell surface receptor signaling pathway	3.86E-10	3.490117574	VIPR1, CALCRL, LAMA1, PIK3CD, CD3G, CD3E, ITGAL, CD3D, SPN, CD79B, **CD79A**, UPK1B, CXCR1, TSPAN8, CXCR3, TSPAN7, BTLA, PTK2B, **IL12B**, FCER1A, CD37, PDK1, CD53, EDN1, FCER1G, **SYK**, FZD6, TNFRSF1B, **AGT**, CD4, CD8A, LCK, CLCF1, CD247, CD22
positive regulation of cell migration	8.56E-10	3.550046405	RET, CSF1R, SEMA7A, FLT1, SEMA3C, LEF1, PIK3CD, CXCR4, IRS2, SEMA3F, AIF1, CORO1A, CX3CL1, AQP1, PAK1, PODXL, **CCL3**, PTK2B, SEMA6B, SPAG9, SEMA4A, EDN1, SEMA4D, MCAM, RDX, SEMA4F, VIL1, TIAM1, **MMP14**, BMP2, **CXCL12**, NUMB, SNAI2
B cell activation	1.96E-09	9.789521906	CD86, CR2, PRKCB, CXCR5, PIK3CD, IKZF3, LAT2, IL4, **CD79A**, BANK1, BLNK, MS4A1, GAPT
positive regulation of T cell proliferation	2.60E-09	6.141983696	CD86, SASH3, SLC4A1, TNFRSF13C, CD3E, ITGAL, AIF1, CORO1A, SPN, SPTA1, CCDC88B, IL4, CD4, PTPRC, CD6, CD28, **IL12B**, CCR7
erythrocyte development	1.14E-08	9.705850778	**BCL6**, TMOD3, HBB-B2, HBB-B1, HBA-A2, HBB-BT, RHAG, HBA-A1, BPGM, HBB-BS, ANK1, GATA1
B cell receptor signaling pathway	1.52E-08	6.352920509	**BLK**, **SYK**, PRKCB, RFTN1, NFATC2, LAT2, CD79B, **CD79A**, ZAP70, PTPRC, TEC, LCK, CD19, PLCG2, CTLA4, PTPN6
chemotaxis	1.66E-08	4.256591337	CX3CR1, CCL22, FLT1, CXCR5, PIK3CD, CXCR4, IL16, LSP1, CXCL15, CX3CL1, **CXCL12**, CXCR1, CXCR3, CCL4, CCR9, ACKR4, **CCL3**, RAC2, ACKR3, CCR7, ACKR2, DOCK2, CCR3
signal transduction	5.81E-08	1.740092769	VIPR1, CHRM1, CD83, GPR65, IRS2, GPR174, RGS5, GPR176, EDNRB, RASSF2, GRB14, RGS1, RASSF5, RASSF6, GPR171, ADORA1, GRB10, UNC5B, HMHA1, ANK3, ANK1, SIGIRR, TIAM1, ACAP1, APLNR, SPARCL1, RIN1, PDE9A, NDRG4, CALCRL, ARHGAP15, STK4, RASAL3, NDRG1, PLCG2, RHPN2, NTSR2, PACSIN1, GPR17, BCL11B, RASL10B, FZD6, RASSF9, PILRA, ICK, GPR183, CSPG4, GPR18, LAT, ARHGAP9, LSP1, HTR2A, ARHGAP4, AKAP12, GPR132, TAGAP, BDKRB2, CCR9, FCER1A, CCR7, CD34, CCR3, RPS6KL1, FCER1G, WNT9B, TRAF1, TLR1, GPRC5B, ADORA2A, RASA3, TLR12, TLR9, CD48, GPRC5C, PTGER4, CX3CR1, PKN3, CXCR5, CXCR4, FYB, GNG2, CNR2, OLFR56, CXCR1, P2RY2, CXCR3, STAT4, PTK2B, S1PR5, ZFP831, P2RY10, PPP1R14C, NOS3, PLCL2, ACKR4, FAS, ACKR3, KRAS, RGS12, ACKR2
receptor internalization	1.32E-07	7.097403382	GRIA2, ACHE, CALCRL, FCER1G, RAMP3, **SYK**, **CAV1**, DNM3, CXCR1, ACKR3, EZR, SNCA, PICALM
positive regulation of peptidyl-tyrosine phosphorylation	1.35E-07	4.324388961	YES1, SEMA4D, **SYK**, ADIPOQ, **IGF2**, VEGFC, LRP4, HTR2A, CD3E, GATA1, **AGT**, IL4, CD4, TEC, EHD4, FCER1A, HCLS1, PTK2B, CSPG4, FGFR3
protein autophosphorylation	2.74E-07	3.222024234	CSF1R, FLT1, TXK, STK4, HK1, STK10, PAK1, TRIM24, EPHB1, **MAP4K1**, NTRK2, YES1, RIPK3, **SYK**, **NEK6**, TNK1, VRK1, ZAP70, HCK, TAOK3, LCK, STK17B, BTK, **SIK1**, MET, FGFR3, FGFR2
response to lipopolysaccharide	3.41E-07	3.103901518	PTGER4, NCF2, PTPN22, **PTGS2**, CXCL15, NPPB, EDNRB, CNR2, PLCG2, CCR7, PCK1, RELT, TIMP4, SNCA, CD96, EDN1, ACE, KCNJ8, NOS2, IL10RA, FMO1, TNFRSF1B, FASL, CD6, TRPV4, CYP1A1, **FAS**, ALDOA
protein phosphorylation	4.67E-07	2.085241378	RET, ITK, FLT1, HK1, STK10, TRIM24, EPHB1, PDK1, MAP4K1, EPHA7, RPS6KL1, RIPK3, SYK, PRKCB, VRK1, HCK, ZAP70, LCK, BTK, SIK1, TRIB3, MET, BLK, CSF1R, LAMA1, PKN3, TXK, STK4, PAK1, NUAK2, CCL3, STAT4, PTK2B, MARK4, IP6K3, MARK1, NTRK2, CDK19, YES1, MLKL, DMPK, NEK6, TNK1, LIMK1, ICK, SBK3, BMP2, TEC, SBK1, TAOK3, STK17B, POMK, MAP3K14, FGFR3, FGFR2
negative thymic T cell selection	1.06E-06	12.478951	SPN, ZAP70, PTPRC, CD28, **FAS**, CCR7, DOCK2, CD3E
phosphorylation	1.38E-06	1.998263396	RET, DGKG, ITK, CIITA, FLT1, PIK3CD, HK1, STK10, EPHB1, PDK1, MAP4K1, EPHA7, RPS6KL1, RIPK3, SYK, PRKCB, VRK1, HCK, ZAP70, PKM, LCK, BTK, SIK1, MET, PRPS2, BLK, CSF1R, PKN3, TXK, AK3, AK4, STK4, PAK1, NUAK2, PTK2B, MARK4, IP6K3, MARK1, NTRK2, CDK19, YES1, DMPK, NEK6, TNK1, LIMK1, ICK, SBK3, PFKL, TEC, SBK1, TAOK3, STK17B, POMK, MAP3K14, FGFR3, FGFR2
peptidyl-tyrosine autophosphorylation	1.54E-06	6.39165783	**BLK**, HCK, ITK, ZAP70, TEC, YES1, **SYK**, LCK, TXK, TNK1, BTK, PTK2B
innate immune response	3.06E-06	2.238411836	CFD, BLK, ITK, CSF1R, NRROS, UNC93B1, TXK, PIK3CD, LY9, PSTPIP1, KLRK1, SLAMF7, PTK2B, SLAMF6, CD55, CR2, YES1, FCER1G, RSAD2, SYK, MX1, TNK1, CD180, LY86, TLR1, ZAP70, HCK, BPIFB1, VNN1, TEC, ADAM15, OAS2, LCK, BTK, TLR9, REL, TLR12, PTX3, IRF5, HC, TRIM10
regulation of cell shape	5.27E-06	3.299291002	CSF1R, SEMA4A, SEMA4D, SH3KBP1, RDX, LST1, VRK1, ARHGAP15, CORO1A, SPTA1, VIL1, HCK, FMNL1, PALMD, RHOJ, CDC42EP3, **CCL3**, PTK2B, PLEKHO1, EZR, ALDOA
positive regulation of calcium-mediated signaling	7.08E-06	8.189311594	ZAP70, CD4, **SYK**, CD8A, CCL4, **CCL3**, FCER1A, CD3E, ITGAL
response to hypoxia	7.73E-06	2.84351097	RYR1, ALAS2, FLT1, CXCR4, PLAT, PLOD1, HIF3A, LOXL2, NPPB, PAK1, ADORA1, PTK2B, EDN1, NOS2, PRKCB, BNIP3, **CAV1**, ADIPOQ, VEGFC, PGF, **MMP14**, BMP2, **CXCL12**, CYP1A1, ALDOA
regulation of cell proliferation	1.60E-05	2.597490902	**BLK**, ITK, CEBPA, TXK, TCF7, PKD2, **PTGS2**, **NDRG1**, CXCL15, MECOM, PTK2B, RELT, JAG2, YES1, NOS2, TNK1, TNFRSF1B, HCK, TEC, **BCL6**, LCK, BTK, MZB1, **FAS**, KCTD11, INHA, FGFR2
peptidyl-tyrosine phosphorylation	3.33E-05	4.36763285	CSF1R, FLT1, **SYK**, HK1, ZAP70, HCK, TEC, LCK, BTK, PTK2B, PTPN6, FGFR3, FGFR2
angiogenesis	3.90E-05	2.467072949	CALCRL, FLT1, HIF3A, **PTGS2**, SOX17, ANPEP, CXCR3, PTK2B, THSD7A, EPHB1, SEMA4A, WARS, **SYK**, NOS3, UNC5B, MCAM, **CAV1**, VEGFC, PGF, **MMP14**, COL4A2, ADAM15, COL4A1, COL8A1, ACKR3, CSPG4, FGFR2
positive regulation of interleukin-10 production	5.26E-05	7.595883218	SASH3, IL4, CD83, FCER1G, CD28, TLR9, **IL12B**, CD34
positive regulation of gene expression	8.46E-05	2.025092926	RET, PTGER4, LEF1, PIK3CD, PTPN22, IKZF1, PKD2, CD3E, LRRC32, SOX17, LAMP3, **CCL3**, TRIM24, STAP1, CD34, IL33, NTRK2, ITGA3, **CAV1**, RDX, NFATC2, ANK3, ETV4, **AGT**, BMP2, MAF, MYCN, LCK, TRPV4, CD28, TLR9, KRAS, SLC26A9, IL7R, EZR, MET, FGFR2
T cell costimulation	9.51E-05	6.98821256	SPN, CD5, **CAV1**, CD28, BTLA, TNFRSF13C, CD3E, ICOS
positive regulation of inflammatory response	0.000119	4.159650334	PTGER4, IL33, GPRC5B, CDK19, ACE, TRPV4, HYAL2, CCL4, TLR9, **CCL3**, CTSS, CX3CL1
sensory perception of pain	0.000184	3.686962796	EDN1, **IL1RN**, NLGN2, ACE, HTR2A, **PTGS2**, AQP1, EDNRB, CNR2, P2RY2, POMK, **IL12B**, SCN3B
positive regulation of renal sodium excretion	0.000187	10.07915273	PTGER4, NPPB, EDN1, EDNRB, ADORA2A, **AGT**
mast cell activation	0.000187	10.07915273	FCER1G, RHOH, LCP2, CD48, **NDRG1**, FYB
intracellular signal transduction	0.000191	1.965434783	SHC4, DGKG, ITK, DGKD, PKN3, RASGRP2, STK4, RASGRP1, NUAK2, RASSF5, PLCG2, BLNK, MARK1, PDK1, **MAP4K1**, EDN1, **SYK**, PRKCB, PLCL2, HMHA1, VAV1, ICK, LAT2, TIAM1, ZAP70, PLCB4, TEC, RASA3, STK17B, BTK, PTPN6, **SIK1**, LCP2, CSPG4, MYZAP, LAT
defense response to virus	0.000234	2.615349012	CD86, IL33, SLFN8, RSAD2, KCNJ8, IFNGR1, UNC93B1, BNIP3, MX1, PRF1, ABCC9, NCR1, PTPRC, OAS2, CD8A, HYAL2, TAGAP, TLR9, **IL12B**, **IRF5**
negative regulation of smooth muscle cell proliferation	0.000267	5.172196796	NPPB, NDRG4, NOS3, **CAV1**, NPR3, ADIPOQ, **IL12B**, TNFAIP3, AIF1
positive regulation of MAPK cascade	0.000272	3.149735229	SPAG9, NTRK2, FLT1, **IGF2**, TNFRSF1B, **AGT**, BMP2, PTPRC, BANK1, BNIP2, **FAS**, RELT, FGFR3, FGFR2, NTSR2
positive regulation of protein phosphorylation	0.0003	2.492399181	PTGER4, CSF1R, SEMA7A, EPHA7, SEMA4D, LRRN3, ADIPOQ, **IGF2**, CXCR4, STK4, AIF1, RASGRP1, SPN, IL4, GPRC5B, PAK1, BMP2, EDNRB, CD6, DOK7, KRAS
defense response to protozoan	0.00033	5.823510467	CCDC88B, IL4, IRF4, TLR12, **IL12B**, IRF8, CD37, BATF

leukocyte chemotaxis	0.000357	6.948506807	CNR2, GPR183, CCL4, CXCR5, **CCL3**, IL16, CORO1A
regulation of ERK1 and ERK2 cascade	0.000409	5.635655291	TIAM1, EPHA7, **SYK**, CLCF1, PTPN6, EPHB1, RASGRP1, FGFR2
apoptotic signaling pathway	0.000452	4.28199299	SPN, FASL, DAPL1, CD5, **CAV1**, CD28, **FAS**, CD3E, RELT, UACA
regulation of immune response	0.000504	5.459541063	SPN, IL4, FCER1G, **SYK**, CLEC12A, PHF11A, TNFRSF13C, CD200
positive regulation of interferon-gamma production	0.000525	4.199646971	SASH3, KLRK1, TXK, PDE4B, **IL12B**, IRF8, CD226, SLAMF6, CD3E, IL27RA
positive regulation of B cell proliferation	0.000646	4.570778564	SASH3, IL4, PTPRC, **BCL6**, CLCF1, GPR183, NFATC2, IRS2, TNFRSF13C
regulation of platelet activation	0.000889	17.4705314	TEC, FCER1G, **SYK**, TXK
apoptotic process	0.000927	1.68575303	CYFIP2, SHC4, BEX2, NCF1, DAPL1, SH3KBP1, GPR65, PRF1, TNFAIP3, LSP1, HIF3A, STK4, PAK1, NUAK2, BCL2L11, EVA1A, MECOM, RASSF5, RASSF6, BNIP2, BCL2A1A, SH3GLB1, EPHA7, PEG3, UNC5B, PRKCB, BNIP3, GZMA, NEK6, TRPV2, GZMB, TRAF1, GULP1, VIL1, TIAM1, FASL, PLSCR3, STK17B, BTK, CHIL1, FAS, TRIB3, FGFR3, FGFR2
positive regulation of alpha-beta T cell proliferation	0.001107	9.926438296	ZAP70, PTPRC, **SYK**, CD28, CD3E
vasoconstriction	0.001345	6.896262395	EDN1, EDNRB, ACE, **CAV1**, BDKRB2, **AGT**
positive regulation of tumor necrosis factor production	0.001349	3.701383771	CD2, SASH3, H2-T23, FCER1G, CCL4, TLR9, **CCL3**, **IL12B**, CCR7, RASGRP1
cellular response to transforming growth factor beta stimulus	0.001717	3.580026926	CX3CR1, EDN1, YES1, **PARP1**, COL4A2, ARG1, NOS3, HYAL2, **CAV1**, ZFP36L2
positive regulation of protein tyrosine kinase activity	0.001726	6.551449275	CSF1R, GPRC5B, ACE, DOK7, UNC119, **AGT**
positive regulation of JUN kinase activity	0.00203	4.36763285	TIAM1, **IL1RN**, EDN1, PAK1, TAOK3, TLR9, PTK2B, FGD2
positive regulation of ERK1 and ERK2 cascade	0.002512	2.207048515	CSF1R, NDRG4, SEMA7A, CCL22, PTPN22, HTR2A, RASGRP1, CX3CL1, BMP2, TRPV4, GPR183, CCL4, CHIL1, **CCL3**, ACKR3, PTK2B, FGFR3, FGFR2, NTSR2
negative regulation of B cell activation	0.002905	12.478951	BANK1, TBC1D10C, **FAS**, TNFAIP3
membrane depolarization	0.003344	5.696912413	EDN1, ADORA2A, **CAV1**, ADIPOQ, SCN3B, SCN1B
chemokine-mediated signaling pathway	0.003348	3.573517787	CCL22, **CXCL12**, CCL4, CCR9, **CCL3**, ACKR3, PTK2B, CXCL15, CX3CL1
integrin-mediated signaling pathway	0.003418	2.817827645	SEMA7A, TEC, FCER1G, ADAM15, **SYK**, ITGA3, TXK, PTK2B, ITGB7, ITGAL, VAV1, LAT
positive regulation of protein binding	0.003674	3.211494743	SPTA1, TIAM1, BMP2, ACE, CALD1, **CAV1**, TRIB3, STK4, CTHRC1, ADD2
positive regulation of nitric oxide biosynthetic process	0.004056	3.882340311	KLRK1, PTK2B, PTX3, **PTGS2**, PKD2, AIF1, **AGT**, ASS1
stimulatory C-type lectin receptor signaling pathway	0.00449	10.91908213	KLRK1, FCER1G, **SYK**, PLCG2
positive regulation of mast cell degranulation	0.005087	6.824426329	IL4, ZAP70, FCER1G, **SYK**, FCER1A
multicellular organism development	0.005165	1.400698582	RYR1, FLT1, TCF23, HIF3A, IKZF1, LCLAT1, ANPEP, CREB3L2, HEY2, STMN1, PHACTR4, SEMA6B, EOMES, EPHA7, EGFL6, TPI1, UNC5B, WNT9B, SHROOM2, PAX5, TNFRSF1B, HIC1, PGF, TMEFF1, MN1, B3GNT5, NUMB, TIMELESS, APLNR, KCTD11, SIK1, FREM2, HEMGN, CEBPA, NOTCH3, SEMA7A, SEMA3C, DHH, TSHZ2, LRP4, TULP3, ATOH8, NNAT, MSX1, RELT, PDLIM7, JAG2, ZFP831, NTRK2, SEMA4A, SEMA4D, NDE1, FZD6, VEGFC, SEMA4F, TDRD7, SORL1, GGN, ICK, TTLL7, BMP2, KIF26B, SNAI2, ACKR3, CSPG4, PICALM
calcium ion transport	0.005248	2.323208963	RYR1, CALCRL, SLC24A3, RAMP3, PRKCB, **CAV1**, TRPV2, PKD2, CORO1A, CACNA1I, TRPV4, MYB, GJA4, **CCL3**, CLCA1
glycolytic process	0.005405	4.246309716	PFKL, TPI1, **PKM**, BPGM, ALDOA, ENO3, HK1
positive regulation of cell proliferation	0.006123	1.571380823	PTGER4, SHC4, CSF1R, CALCRL, NLGN2, LEF1, IRS2, HTR2A, PTGS2, PLAC8, PAK1, EDNRB, EPCAM, CXCR3, RAC2, PTK2B, MARK4, NTRK2, EDN1, ST8SIA1, VEGFC, NCCRP1, AGT, PGF, ACER2, IL4, TIAM1, HCK, CXCL12, MYCN, FASL, CLCF1, ACER3, MZB1, HCLS1, PTPN6, KRAS, FGFR3, FGFR2
negative regulation of cytokine-mediated signaling pathway	0.006508	9.705850778	SIGIRR, **IL1RN**, PTPRC, **CAV1**
response to drug	0.007186	1.739322816	RET, CD86, ATP1A3, WFDC1, AK4, HTR2A, **PTGS2**, ENO3, AQP1, NPPB, **CCL3**, PTK2B, TIMP4, SNCA, TGIF1, ARG1, ITGA3, PRKCB, **IGF2**, VEGFC, NFATC2, PGF, IL4, ADORA2A, LOX, LCK, CYP1A1
cellular response to lipopolysaccharide	0.00759	1.985287659	CD86, CX3CR1, CDK19, NOS2, ARG1, GFI1, CD180, TNFAIP3, TNFRSF1B, MIR147, EDNRB, KLRK1, PLSCR3, TNIP3, TLR9, PDE4B, **IL12B**, STAP1, IRF8
platelet activation	0.008064	3.919670507	ENTPD1, **SYK**, VWF, FZD6, RASGRP2, VAV1, CX3CL1
cell chemotaxis	0.009086	2.799764648	CCL22, **CXCL12**, BIN2, SAA3, GPR183, LEF1, CCL4, **CCL3**, EPHB1, CX3CL1
cellular response to mechanical stimulus	0.009086	2.799764648	PTGER4, NPPB, NOS3, BNIP3, **CAV1**, **FAS**, **PTGS2**, MAP3K14, **AGT**, AQP1
patterning of blood vessels	0.009128	3.821678744	EDN1, **CXCL12**, FLT1, COL4A1, LEF1, CXCR4, STK4
B cell differentiation	0.011525	2.696069661	CD79B, **CD79A**, CR2, PTPRC, **BCL6**, CLCF1, MYB, PLCG2, IKZF1, RAG1
extrinsic apoptotic signaling pathway in absence of ligand	0.011553	3.639694042	IL4, BCL2L11, UNC5B, **FAS**, BCL2A1D, BCL2A1A, BCL2A1B
neutrophil activation involved in immune response	0.011787	16.37862319	ZAP70, FCER1G, **SYK**
maintenance of blood-brain barrier	0.011787	16.37862319	BDKRB2, **PTGS2**, ABCB1A
cellular response to interferon-gamma	0.012114	2.890345269	CIITA, EDN1, CCL22, NOS2, CCL4, **CCL3**, **IL12B**, AIF1, CX3CL1
T cell homeostasis	0.012494	4.226741468	GPR174, BCL2L11, RIPK3, **FAS**, CORO1A, RAG1
MAPK cascade	0.013171	2.848456207	RET, DOK2, PLVAP, TAOK3, **CAV1**, **CCL3**, PTK2B, FGFR3, ZFP36L2
neutrophil chemotaxis	0.013171	2.848456207	CCL22, FCER1G, **SYK**, CCL4, PDE4B, **CCL3**, VAV1, CXCL15, CX3CL1
actin cytoskeleton organization	0.013379	2.153058447	TMOD3, CORO1A, SPTA1, PAK1, FMNL1, NUAK2, **BCL6**, RHOJ, RAC2, CCR7, KRAS, DOCK2, PDLIM7, PHACTR4
regulation of GTPase activity	0.015484	2.768218004	TIAM1, NTRK2, **BCL6**, RASA3, RDX, RASAL3, ADAP1, RASGRP1, VAV1
vasodilation	0.016453	4.963219148	EDNRB, ADORA2A, KCNJ8, BDKRB2, **AGT**
negative regulation of transcription from RNA polymerase II promoter	0.016862	1.407947489	NOTCH3, CEBPA, CIITA, SPI1, SATB1, SLFN1, GFI1, LEF1, TCF7, IKZF1, GATA1, EDNRB, SOX17, MXI1, MYB, HEY2, MSX1, EOMES, TGIF1, EDN1, SEMA4D, PEG3, CAV1, FST, NFATC2, HFE2, PAX5, HIC1, BMP2, MAF, BCL6, FASL, TRPV4, PKIA, CRY1, TIMELESS, REL, IRF8, SNAI2, HCLS1, SIK1, TRIB3, EZR, MET, FGFR3, RCOR2, FGFR2
calcium-mediated signaling	0.017698	3.323198908	LAT2, EDN1, CXCR3, **CCL3**, CXCR4, PPP1R9A, LAT
negative regulation of canonical Wnt signaling pathway	0.018261	2.355096145	BMP2, SOX17, **CAV1**, FZD6, LEF1, PTPRO, LRP4, SNAI2, STK4, NKD2, CTHRC1
brown fat cell differentiation	0.018292	3.853793691	PLAC8, CEBPA, BNIP3, ADIPOQ, SLC2A4, **PTGS2**
endocytosis	0.019247	1.930445459	SH3GL3, SH3KBP1, **CAV1**, STAB2, LRP4, SNX30, CD209A, SORL1, DNM3, PSTPIP1, FCHO1, NOSTRIN, UNC119, RIN1, PACSIN1, PICALM
positive regulation of neuron differentiation	0.019427	2.332231134	SPAG9, TGIF1, GPRC5B, BMP2, BRINP1, **CXCL12**, **BCL6**, BNIP2, KCTD11, GDPD5, ETV5
regulation of apoptotic process	0.020667	1.86557182	TRAF1, TNFRSF1B, IKZF3, **AGT**, BMP2, RASSF2, BCL2L11, SELL, LCK, RASSF5, RASSF6, TRIM24, SNAI2, **FAS**, INHA, BCL2A1A, RELT
regulation of inflammatory response	0.020846	2.864021541	IL4, SEMA7A, PGLYRP2, **BCL6**, CLCF1, TLR9, **AGT**, MGLL
brain development	0.021023	1.819847021	EOMES, NDRG4, EPHA7, SHROOM2, TULP3, CXCR4, IRS2, AK4, **CXCL12**, BCL2L11, COL4A1, SNPH, B3GNT5, STMN1, NNAT, POMK, **FAS**, MET
T cell proliferation	0.022242	4.549617552	PTPRC, **CXCL12**, EBI3, CXCR4, DOCK2
positive regulation of synaptic transmission, glutamatergic	0.022242	4.549617552	NTRK2, NLGN2, ADORA2A, PTK2B, **PTGS2**
regulation of blood pressure	0.022605	2.817827645	EDN1, EDNRB, CYP4F18, ACE, NPR3, **PTGS2**, CD34, **AGT**
branching morphogenesis of an epithelial tube	0.023005	3.639694042	**MMP14**, PAK1, MYCN, WNT9B, TIMELESS, MET
macrophage chemotaxis	0.02378	6.2394755	CX3CR1, EDNRB, **CCL3**, CX3CL1

striated muscle cell differentiation	0.02378	6.2394755	SPAG9, **IGF2**, BNIP2, KRAS
drug transmembrane transport	0.02378	6.2394755	ATP8B1, ABCB4, ABCB1A, **ABCG2**
positive regulation of phosphatidylinositol 3-kinase signaling	0.026423	2.729770531	NTRK2, FLT1, SEMA4D, UNC5B, CD28, HCLS1, PTPN6, **AGT**
T cell chemotaxis	0.027718	10.91908213	CXCR3, GPR183, **CCL3**
positive regulation of gamma-delta T cell differentiation	0.027718	10.91908213	PTPRC, **SYK**, LCK
aging	0.027752	1.89348245	CD86, CIITA, ARG1, HTR2A, PAX5, RETN, PPP1R9A, TNFRSF1B, ENO3, **AGT**, EDNRB, CYP1A1, PCK1, CTSC, SNCA
positive regulation of cell adhesion mediated by integrin	0.028736	5.823510467	RET, ZAP70, **SYK**, PTPN6
activation of phospholipase C activity	0.028736	5.823510467	ITK, TXK, HTR2A, **AGT**
ERK1 and ERK2 cascade	0.029129	4.199646971	PTGER4, FGFR3, ZFP36L2, CCR3, **AGT**
cellular response to tumor necrosis factor	0.029155	2.183816425	CEBPA, EDN1, CCL22, HYAL2, CCL4, CHIL1, **CCL3**, SLC2A4, PCK1, ZFP36L2, CX3CL1
cellular response to interleukin-1	0.029598	2.456793478	EDN1, CCL22, SAA3, HYAL2, CCL4, CHIL1, **CCL3**, PCK1, CX3CL1
negative regulation of cell proliferation	0.030328	1.535495924	CSF1R, CEBPA, SLFN1, **PTGS2**, STK4, **NDRG1**, GATA1, RASSF5, ADORA1, TRIM24, BDKRB2, PTK2B, CD37, MSX1, TGIF1, BCL11B, NOS3, **CAV1**, TRPV2, VEGFC, **DHCR24**, **AGT**, BMP2, ADORA2A, **BCL6**, FGFR3, FGFR2
positive regulation of catalytic activity	0.031435	3.359717577	APH1A, **CAV1**, **IGF2**, WNT9B, **AGT**, PHACTR4
cell differentiation	0.031963	1.343887031	HEMGN, BLK, NOTCH3, SEMA7A, EHF, FLT1, SEMA3C, DAPL1, TCF23, LRP4, PIK3CD, RASGRP1, FCRLA, KLRK1, MECOM, ANPEP, ATOH8, STMN1, THSD7A, PDLIM7, JAG2, SEMA6B, EOMES, NTRK2, SEMA4A, EGFL6, YES1, SEMA4D, NDE1, TNK1, VEGFC, SEMA4F, TDRD7, PAX5, TEX15, ETV5, PGF, BATF, GGN, TTLL7, HCK, BMP2, TEC, PTPN6, KCTD11, SIK1, CSPG4, FGFR3
negative regulation of cysteine-type endopeptidase activity involved in apoptotic process	0.032937	2.607542	ADORA2A, LAMP3, LEF1, **DHCR24**, BCL2A1D, AQP1, SNCA, RAG1
positive regulation of endothelial cell proliferation	0.032937	2.607542	BMP2, **CXCL12**, ARG1, **CAV1**, VEGFC, FGFR3, CCR3, PGF
lipid storage	0.032995	4.044104491	GM2A, **CAV1**, HEXA, CRY1, B4GALNT1
monocyte chemotaxis	0.034622	3.275724638	CCL22, FLT1, PTPRO, CCL4, **CCL3**, CX3CL1
cytokine-mediated signaling pathway	0.035783	1.944494077	CX3CR1, CSF1R, CEBPA, **IL1RN**, IFNGR1, ASPN, CX3CL1, IL2RB, IL3RA, STAT4, PTPN6, KRAS, **IRF5**
protein homooligomerization	0.037206	1.77365801	SPAG9, ENTPD1, DGKD, VWF, RIPK3, **CAV1**, ADIPOQ, PRF1, AK3, EHD4, STOM, **FAS**, KCTD14, AQP11, KCTD11, KCTD17
positive regulation of natural killer cell mediated cytotoxicity directed against tumor cell target	0.037643	9.359213251	KLRK1, **IL12B**, CD226
regulation of sensory perception of pain	0.038001	3.195828915	EDN1, EDNRB, ADORA1, **CCL3**, CTSS, MGLL
positive regulation of multicellular organism growth	0.038001	3.195828915	VIL1, FOXS1, **IGF2**, IKZF1, EZR, **AGT**
positive regulation of cytosolic calcium ion concentration	0.039099	1.91821713	PTGER4, EDN1, HTR2A, PKD2, **AGT**, EDNRB, TRPV4, P2RY2, CXCR3, **CCL3**, PLCG2, BDKRB2, PTK2B
cellular calcium ion homeostasis	0.039438	2.183816425	RYR1, KEL, SLC24A3, DMPK, PRKCB, TRPV4, **CAV1**, **CCL3**, HTR2A, PKD2
positive regulation of tyrosine phosphorylation of Stat3 protein	0.046308	3.639694042	PTGER4, CSF1R, CLCF1, **IL12B**, FGFR3
positive regulation of B cell differentiation	0.046585	4.852925389	ZAP70, **MMP14**, **SYK**, IKZF1
positive regulation of cardiac muscle hypertrophy	0.046585	4.852925389	EDN1, **PARP1**, **AGT**, PDE9A
induction of positive chemotaxis	0.046585	4.852925389	**CXCL12**, VEGFC, IL16, PGF
cytoskeleton organization	0.048642	2.099823486	VIL1, **BLK**, HCK, FMNL1, SH3KBP1, **CCL3**, LSP1, DOCK2, PACSIN1, MARK1
negative regulation of leukocyte apoptotic process	0.048694	8.189311594	**CXCL12**, HCLS1, CCR7
negative regulation of smooth muscle contraction	0.048694	8.189311594	CALCRL, NCF1, **PTGS2**
positive regulation of granulocyte macrophage colony-stimulating factor production	0.048694	8.189311594	TLR9, **IL12B**, RASGRP1
negative regulation of Notch signaling pathway	0.051318	3.522284557	**MMP14**, **BCL6**, HEY2, NUMB, GDPD5
programmed cell death	0.051318	3.522284557	**PKM,** RIPK3, MLKL, STK17B, RELT
positive regulation of peptidyl-serine phosphorylation	0.052065	2.360882622	NTRK2, PAK1, **CAV1**, HCLS1, STK4, MET, **AGT**, SNCA
regulation of blood coagulation	0.053499	4.597508263	**CAV1**, FAM46A, STAB2, CD209A
positive regulation of myoblast fusion	0.053499	4.597508263	CD53, IL4, **CXCL12**, NFATC2
response to glucocorticoid	0.055266	2.329404187	**IL1RN**, ABCA3, ADIPOQ, **FAS**, KRAS, **PTGS2**, AIF1, ASS1
positive regulation of extrinsic apoptotic signaling pathway	0.056611	3.412213164	PTPRC, HYAL2, **CAV1**, **FAS**, **AGT**
positive regulation of apoptotic process	0.057147	1.49933665	EPHA7, CDK19, ACE, NOS3, BNIP3, GZMA, STK4, **PTGS2**, UACA, ALDH1A3, BMP2, RASSF2, BCL2L11, KLRK1, **BCL6**, FASL, RASSF6, FAS, CTLA4, **IRF5**, BCL2A1D, BCL2A1A, BCL2A1B
lung development	0.058044	1.937256506	CEBPA, **MMP14**, MYCN, LOX, ITGA3, ARG1, NOS3, TIMELESS, CHIL1, AARD, FGFR2
Notch signaling pathway	0.060638	1.921758454	CFD, JAG2, APH1A, **NOTCH3**, CEBPA, BMP2, HEY2, NUMB, SNAI2, GZMB, TIMP4
regulation of phagocytosis	0.060747	7.279388084	**BLK**, HCK, **SYK**
lymphocyte differentiation	0.060747	7.279388084	SPI1, **LY6D**, IKZF1
negative regulation of heterotypic cell-cell adhesion	0.060747	7.279388084	**IL1RN**, ADIPOQ, TNFAIP3
negative regulation of gene expression	0.061763	1.565755173	TGIF1, ACE, NOS2, SLC35C2, KEAP1, PTPN22, CD3E, AIF1, BMP2, MYCN, HEY2, CD28, **CCL3**, REL, MSX1OS, FGFR3, CD34, MET, PICALM
Ras protein signal transduction	0.062404	2.787850755	DOK2, DOK3, **DHCR24**, KRAS, RASGRP1, LAT
positive regulation of transcription from RNA polymerase II promoter	0.064099	1.251030515	EHF, CIITA, SPI1, FHL5, HIF3A, IKZF1, PKD2, CD3D, IKZF3, MECOM, SOX17, HYAL2, CREB3L2, MYB, HEY2, EOMES, EDN1, PARP1, PEG3, HFE2, PAX5, ETV4, ETV5, MYCN, MAF, IRF4, TLR9, HCLS1, NCOA7, IRF5, MET, SKAP1, CEBPA, NOTCH3, BEX2, TXK, LEF1, GATA1, PLAC8, EPCAM, CCL3, STAT4, MSX1, ZBTB7C, IL33, YES1, BCL11B, IGF2, NFATC2, POU2F2, BATF, IL4, BMP2, CD28, REL, CRLF3, FGFR2
branching involved in ureteric bud morphogenesis	0.067162	2.729770531	BMP2, WNT9B, TIMELESS, PKD2, **AGT**, PGF
response to mechanical stimulus	0.067483	2.426462695	**MMP14**, **CXCL12**, LCK, **CAV1**, CHIL1, PTK2B, RETN
liver development	0.069173	2.068878719	CEBPA, **PKM**, VWF, ARG1, COBL, AK4, KRAS, PKD2, MET
protein localization	0.069275	2.211459671	CLIC5, **BCL6**, KIF26B, **CAV1**, KIF5B, LRP4, **DHCR24**, GGN
positive regulation of GTPase activity	0.071659	1.79491761	RGS4, CCL22, SEMA4D, RASGEF1B, RGS1, CCL4, **CCL3**, CCR7, RASGRP2, RASGRP1, VAV1, CX3CL1
positive regulation of smooth muscle cell proliferation	0.073079	2.183816425	**NOTCH3**, EDN1, CALCRL, RETN, **PTGS2**, AIF1, FGFR2, CX3CL1
artery smooth muscle contraction	0.073688	6.551449275	EDN1, HTR2A, **AGT**
response to bacterium	0.074164	3.11973775	VIL1, NCF1, MECOM, **CAV1**, IRF8
bone mineralization	0.074164	3.11973775	BMP2, **PTGS2**, FGFR3, ASPN, FGFR2

kidney development	0.074725	1.847844667	ACE, BCL2L11, KCNJ8, ADAMTS1, WNT9B, TIMELESS, LRP4, AQP11, PKD2, PCSK5, **AGT**
regulation of sodium ion transport	0.07694	3.970575318	DMPK, NOS3, **SIK1**, SCN1B
cell morphogenesis	0.077005	2.156855729	**BCL6**, UNC93B1, LST1, PTPRO, SHROOM2, MSX1, IL7R, STK4
extracellular matrix organization	0.077506	1.915628443	ERO1LB, EGFL6, COL4A2, LAMA1, LAMC3, COL4A1, ELN, ABI3BP, FBLN1, **AGT**
negative regulation of ERK1 and ERK2 cascade	0.080675	2.316168936	PTPRR, **CAV1**, ADIPOQ, TBC1D10C, TIMP3, FBLN1, EZR
peptidyl-serine phosphorylation	0.084083	1.806163961	**MAP4K1**, SBK1, **SYK**, DMPK, PRKCB, PKN3, **NEK6**, VRK1, STK4, PDK1, HK1
memory	0.085219	2.104883301	CX3CR1, **IL1RN**, ITGA3, **IGF2**, ATP1A3, HTR2A, **PTGS2**, RIN1
signal transduction involved in regulation of gene expression	0.0856	3.797941609	**PARP1**, SOX17, EPCAM, MSX1
renal system process	0.08741	5.955862978	**FAS**, **AGT**, SLC4A5
macrophage activation involved in immune response	0.08741	5.955862978	IL33, ZAP70, **SYK**
natural killer cell activation	0.08741	5.955862978	KLRK1, IL2RB, **IL12B**
regulation of the force of heart contraction by chemical signal	0.089383	21.83816425	NOS3, **CAV1**
glucose metabolic process	0.090213	2.24804632	TPI1, **PKM**, ADIPOQ, **IGF2**, PCK1, PGM1, PDK1
carbohydrate metabolic process	0.090858	1.590157591	GAA, HEXA, **IGF2**, HK1, GLB1L2, PPP1R2, PFKL, LDHA, HYAL2, CHST10, CHIL1, CHIL3, CHST2, PGM1, PDK1
leukocyte cell-cell adhesion	0.094651	3.639694042	PTPRC, **SYK**, ITGAL, EZR
collagen catabolic process	0.094651	3.639694042	**MMP14**, ADAM15, CTSK, CTSS
positive regulation of activated T cell proliferation	0.094651	3.639694042	CD86, IL4, **IGF2**, **IL12B**
cell-cell signaling	0.099405	1.908189109	GJC1, EDN1, DHH, GJA4, WNT9B, **CCL3**, FGFR3, GATA1, FGFR2
cell migration	0.099621	1.600703139	FLT1, SH3KBP1, SHROOM2, NFATC2, CXCR4, CORO1A, SORL1, TIAM1, PSTPIP1, **MMP14**, PODXL, **IL12B**, SNAI2, CTHRC1
defense response to bacterium	0.099621	1.600703139	FCER1G, **SYK**, NOS2, NCF1, LYZ2, LYZ1, PRG2, STAB2, SPN, TLR1, PLAC8, ADAMTS4, SLAMF8, IRF8
