# Supplementary material for: Endothelial senescence mediates hypoxia-induced vascular remodeling by modulating PDGFB expression
Source: Front Med (Lausanne). 2022 Sep 20;9:908639. doi: 10.3389/fmed.2022.908639 (PMC9530050; doi:10.3389/fmed.2022.908639)
Supplement: Supplementary file 9 [file Data_Sheet_6.PDF]

Proteins	Normoxia1 (Spectrum Count)	Normoxia2 (Spectrum Count)	Normoxia3 (Spectrum Count)	Hypoxia1 (Spectrum Count)	Hypoxia2 (Spectrum Count)	Hypoxia3 (Spectrum Count)
Fn1	98	34	97	108	88	87
Tln1	132	22	70	94	70	71
Lamc1	75	30	83	90	102	88
Flna	119	8	47	91	64	58
Thbs1	71	26	74	79	63	62
Tnc	45	51	114	54	101	75
Lama5	55	34	70	60	90	78
Lamb2	45	21	69	56	93	70
Cbr2	50	21	47	79	38	46
Actg1	68	8	53	73	52	41
Hspg2	40	19	61	39	81	69
Lama3	25	11	50	31	69	56
Igkc	74	6	11	34	10	51
Sptan1	27	14	45	37	58	51
Cltc	55	7	28	46	42	29
Nid1	27	16	40	35	60	51
Sptbn1	25	12	43	31	49	56
Fasn	30	10	29	34	34	22
Myh9	47	14	36	23	34	31
Hist1h2bf	16	9	73	13	36	58
Lamb1	10	8	25	33	55	40
Vcp	15	15	43	20	40	37
Lgals3bp	13	22	34	15	45	39
Actc1	27	5	14	35	26	42
Ighm	39	2	6	6	8	108
Dpysl2	21	11	35	29	29	21
Acta1	0	0	0	0	0	41
C3	20	14	29	13	31	38
Tuba1b	26	7	22	29	26	22
Tuba1a	24	7	22	30	25	23
Tubb4b	21	8	22	26	24	27
Ager	26	11	19	24	22	17
Tubb5	19	10	23	24	23	25
Tgfbi	15	13	32	13	40	19
Col4a1	16	8	24	13	35	28
Msn	13	9	35	14	22	35
Agrn	12	10	27	16	40	25
Tgm2	17	3	21	23	26	19
Lama4	9	8	19	18	38	32
Tuba4a	23	6	20	24	21	18
Flnb	10	4	14	30	27	19
Fbn1	4	7	42	14	35	25
Aldh1a1	13	6	21	21	23	19
Actr3	19	13	17	14	19	17
Hist1h3b	13	11	42	12	19	25
Itgb1	12	2	18	19	23	26
Anxa2	11	13	19	21	22	24
Lama2	2	1	14	25	31	25
Hist2h2ac	6	18	31	4	18	26
Eef2	18	7	19	16	19	16
Lamb3	8	3	19	13	34	24
Ace	11	4	10	26	22	18
Actn1	23	2	10	11	18	17
Gsn	12	7	15	16	17	16
Inmt	11	3	22	19	23	15
Bcam	10	3	16	15	23	20
Actn4	20	2	14	12	26	15
Pkm	12	2	13	18	17	21
Hspa8	19	5	11	13	13	17
Dync1h1	17	2	9	22	12	9
Ctsb	7	11	17	12	24	20
Col4a4	9	11	17	9	23	23
Uba1	16	3	14	13	10	11
Hist1h2ab	0	10	27	0	13	19
Pxdn	0	7	27	3	32	25
Col4a2	10	7	22	7	22	15
Aldh2	8	6	15	17	12	11
Hba	10	4	7	11	10	14
Xdh	9	1	12	18	14	13
Iqgap1	15	2	9	17	9	8
1	25	0	0	18	0	1
Col1a1	0	11	41	0	15	17
Vcl	12	6	10	13	17	16
Cap1	15	4	11	10	13	11
Selenbp1	5	2	9	16	16	17
Actr2	24	3	3	14	7	2
Col4a3	14	4	18	10	13	12
Ehd4	15	0	13	13	11	11
Fgg	18	2	8	13	12	10
Alb	7	13	15	7	11	17
Ehd2	10	2	10	17	12	12
H2afx	3	11	21	4	12	13
H2afy	9	11	16	8	9	11
Vwf	9	1	5	15	15	14
Hbb-b1	8	2	2	10	6	10
Eef1a1	10	4	9	10	6	8
C4b	12	2	14	4	10	14
Ppia	8	5	12	10	9	7
Lamc2	6	1	11	12	21	11
Myh8	1	0	2	7	7	54
Myo1c	9	0	3	16	6	8
Myh6	3	1	4	13	16	19
Hist1h1e	1	2	12	7	16	6
Fgb	14	1	5	8	12	7
Myh1	0	0	0	6	0	51

Itga3	5	3	7	8	10	11
Clic5	9	0	10	12	6	9
Hist1h4a	6	7	14	5	9	10
Hist1h1c	2	1	12	6	11	5
Tkt	8	1	4	13	6	8
Anxa5	1	6	14	2	11	21
Ehd1	6	1	10	11	8	8
Chil3	12	2	5	3	9	9
Gapdh	9	1	5	11	12	7
Cd36	9	5	6	10	5	8
Plec	10	0	4	11	6	12
Col6a1	2	3	11	8	13	10
Vat1	7	3	10	10	9	7
Pfn1	5	2	12	7	10	9
Cfl1	10	1	3	10	5	7
Eno1	8	0	6	9	6	9
Cavin1	5	4	8	6	11	6
Cdh5	4	1	6	6	11	7
Atp5f1b	10	1	3	12	3	3
Vim	6	2	6	5	11	5
Aqp1	7	1	6	9	5	10
Rsu1	8	4	8	7	5	6
Gnb2	4	1	12	7	10	6
Rack1	9	1	5	10	5	4
Aldoa	2	0	5	5	16	12
Hsp90ab1	8	1	8	5	8	5
Npnt	7	1	7	6	8	6
Psap	1	1	13	0	17	12
Hmgb1	7	5	8	4	2	3
Dpp4	6	1	6	10	5	3
Rap1b	12	3	5	7	2	4
Cct5	9	3	4	7	4	4
Gnb1	6	2	7	6	8	7
Ap2b1	3	2	10	4	5	8
Lmna	2	2	9	4	12	7
Arpc2	5	2	8	6	2	7
Ywhaq	4	1	6	11	5	5
Ppp2r1a	6	2	3	7	3	5
Atp1a1	4	3	4	5	5	5
Ncl	7	2	5	8	2	1
Cfh	6	0	6	5	9	4
Pgk1	5	1	5	8	3	6
Lcp1	4	1	5	6	5	4
Itga1	1	0	7	7	8	7
Prdx6	2	2	8	5	7	6
Ctnna1	3	1	9	4	8	7
Hspa5	9	0	3	3	4	3
Lpl	2	3	9	2	8	10
Clu	3	2	8	3	6	7
Cct6a	6	2	5	10	1	1
Serpinh1	6	0	3	9	3	4
Hsp90aa1	7	0	6	3	5	0
Tubb1	16	0	2	3	3	4
Anxa6	0	1	13	0	9	12
Nid2	2	2	6	5	9	7
Arf1	4	1	9	4	6	8
Ldha	2	2	6	3	10	9
Myh14	0	0	12	3	9	3
Col6a2	3	1	4	3	9	7
Ywhaz	5	1	4	9	5	3
Clic1	6	1	4	6	4	4
Thbd	7	0	3	11	5	3
Fermt3	20	0	0	3	0	3
Pdcd6ip	4	1	6	6	4	2
Arhgdia	2	2	5	5	4	4
Hspa12b	2	1	3	4	6	7
Tcp1	4	1	7	6	4	4
Cct4	5	1	4	5	4	3
Coro1c	5	2	4	6	4	3
Tagln2	5	1	3	7	6	4
Anxa3	1	3	9	2	6	9
Gdi2	3	2	5	3	7	3
Tpp1	3	0	4	3	6	7
Macf1	4	0	2	9	4	0
Coro1a	5	2	5	5	4	2
Vars	10	0	7	5	1	1
H2afz	0	9	10	0	3	9
Adk	4	2	6	3	5	5
Ptgfrn	3	1	3	5	11	5
Atp6v1a	5	0	1	4	8	1
Myh11	0	0	6	4	5	7
Fbln1	1	2	5	2	7	6
Snrnp200	2	2	6	5	4	4
Wars	2	1	3	6	8	5
Icam1	3	2	4	6	4	3
44811	4	1	5	4	5	3
Ap2a2	4	1	7	3	6	3
Ywhag	4	1	7	3	6	5
Cdc42	4	0	1	6	5	4
Capzb	6	0	1	8	2	2
Jup	6	0	2	6	2	3
Ctsd	0	1	10	0	8	8
Col1a2	0	3	14	0	6	5
Vps35	6	0	2	4	2	2
Acly	7	0	1	5	1	0
Cd9	3	1	8	4	2	3

Clic4	2	0	4	4	5	6
Ltbp4	0	0	1	4	11	5
Ap2a1	3	0	5	2	5	4
Lrp1	5	0	2	5	5	7
Cand1	4	1	5	3	4	3
44806	3	0	3	5	3	3
Capza2	6	0	2	5	1	3
Wdr1	2	0	8	3	5	6
Gnai2	3	1	4	3	4	4
Ubb	2	1	7	2	4	6
Rhoa	5	1	3	4	3	4
Dpep1	1	0	4	6	3	3
Ywhab	2	0	3	4	5	4
Marcks	3	1	5	3	3	4
Arpc1b	4	1	3	1	5	3
Fmo2	0	1	3	5	8	5
Itga2b	11	0	0	7	0	0
Itga8	3	0	2	4	3	3
Psme2	4	0	3	4	2	1
Kpnb1	2	0	3	6	2	1
S100a11	1	0	2	6	5	4
Serpine1	1	0	5	1	8	5
Bgn	0	3	4	1	10	3
Postn	0	0	6	0	6	6
Ehd3	5	0	0	0	0	3
Gstp1	3	2	4	1	3	3
Cavin2	3	1	4	3	3	3
Psma2	3	2	3	1	2	4
Itih2	1	3	3	2	5	3
Cct7	3	1	5	4	2	1
Ctnnb1	2	1	4	2	3	4
Eef1g	5	0	2	3	2	3
Mrc1	2	2	2	4	5	3
Anxa1	2	0	2	3	2	3
Samhd1	1	5	4	1	0	2
Ap2m1	1	0	6	1	8	5
Crip2	3	0	3	2	4	1
H2-K1	2	0	1	5	3	2
Fermt2	1	0	6	1	1	8
Krt77	4	2	2	3	1	1
Capn2	3	0	2	3	6	2
Cct2	4	0	4	3	2	2
Atp6v1b2	5	0	0	3	2	2
Myl6	3	0	2	4	4	0
Plod1	0	1	2	1	6	8
Nptx1	0	0	3	0	8	6
Gpi	0	0	5	0	6	8
Ppp1cb	4	0	1	3	2	2
Actr1a	4	0	1	3	2	3
Tspan8	1	0	3	2	3	4
Plod3	2	2	3	0	2	3
Gstm1	0	2	6	0	4	6
Fbln2	0	2	6	0	4	7
Gdi1	0	0	0	0	4	0
Serpinf1	1	2	4	1	2	4
Fga	4	1	1	1	3	1
F2	2	0	4	1	3	4
Cct3	4	0	0	2	0	1
Ezr	1	0	2	3	0	0
Olfml3	0	1	5	0	6	5
Gprc5a	2	0	3	1	3	3
Krt19	4	0	1	3	2	2
Ywhah	1	0	3	1	4	5
Pura	1	0	5	2	4	1
Csrp1	1	0	3	1	4	3
Rplp1	2	0	2	3	1	1
Des	2	0	1	1	3	1
Copb2	3	0	2	2	3	1
Pdia6	7	0	1	0	2	1
Mmp19	4	0	0	1	1	0
Psmb1	0	1	2	0	4	6
H2afy2	1	0	3	0	4	4
Col3a1	0	0	9	0	2	1
Thbs4	1	1	1	3	1	2
Psma7	1	0	2	1	3	4
Esd	2	0	3	1	3	2
Cyfip1	1	0	4	3	1	3
Rpsa	2	0	3	1	4	3
Ces1d	0	1	3	1	4	5
Ppp2ca	0	0	2	3	5	2
Pebp1	1	1	4	1	2	1
Pecam1	1	0	2	3	1	2
Plg	2	1	2	0	2	3
Cct8	3	0	2	2	1	1
Rac1	2	1	1	1	5	1
Anxa4	0	1	2	1	4	4
Arhgap1	0	0	1	4	1	3
Slc9a3r2	2	0	0	4	1	0
Cemip2	1	0	0	2	4	4
Ywhae	1	0	4	3	0	4
Mcam	0	0	0	4	3	1
Pls3	0	0	5	1	3	2
Col12a1	0	0	7	0	5	1
Slc44a2	2	0	3	0	1	1
Sars	1	0	3	2	1	0
Cfb	0	3	2	1	2	3

Ddah2	3	0	3	1	2	0
Mfge8	1	0	2	2	4	1
1	0	0	0	0	0	3
Mdh2	1	1	2	1	1	2
Dnpep	3	0	1	2	1	1
Itgb2	3	1	1	1	1	0
Tpi1	1	0	2	1	3	2
Gnaq	2	0	1	1	2	1
Nrp1	1	0	1	2	3	0
Hnrnpf	3	0	1	3	2	0
Itga6	2	0	1	4	1	1
Krt75	2	1	0	2	1	0
Lyz2	1	2	4	0	3	2
Eif3b	3	0	0	3	1	0
Sod1	0	1	4	0	4	2
Pgls	1	0	0	3	0	0
Slc25a4	2	0	0	4	0	0
Ttn	0	0	0	1	0	10
Cd47	1	0	2	1	2	2
Fhl1	1	0	1	1	2	1
Gnas	0	1	2	1	2	1
Pafah1b1	1	0	0	2	1	1
Comp	1	1	2	0	2	4
Prdx1	2	0	1	2	0	0
Vtn	2	0	2	0	2	2
Hmgb2	1	1	3	0	1	0
Nt5e	0	0	2	2	2	3
Pgm2	0	1	4	0	2	2
Prpf19	0	0	4	0	1	1
Igf2r	1	1	1	0	2	3
Ly6c1	0	0	2	1	3	3
Tinagl1	0	2	2	2	2	3
Abcb1a	0	0	1	2	3	4
Marco	0	1	3	1	4	2
1	4	0	0	1	0	1
Anxa7	1	0	2	1	2	2
Krt10	1	1	0	1	1	2
Fmo1	1	2	2	1	0	0
H2-D1	1	0	0	2	3	1
Rab11a	2	0	1	2	0	1
Serpinc1	0	0	2	1	2	2
Apob	0	0	2	1	1	3
Ppm1f	0	0	1	2	1	4
Rab7a	1	0	4	0	0	1
Kif5b	2	0	1	4	0	0
Lta4h	0	0	1	0	4	4
Lmnb1	1	1	2	1	1	1
Krt7	2	1	1	1	1	2
Psma5	1	0	1	1	1	2
Rras	1	0	1	2	1	0
Tspan9	0	0	2	1	2	2
Wasf2	2	0	0	1	1	1
Tppp3	1	0	2	2	1	0
Anp32a	0	0	1	2	3	1
Psma6	2	1	2	0	2	1
Gucy1a1	2	1	2	0	1	1
Enpep	2	0	2	2	0	1
Mmp12	1	1	4	1	1	0
Acp5	0	0	1	1	4	2
Alcam	0	0	0	2	2	1
Hnrnpu	1	0	0	2	1	0
Vps26a	3	0	0	2	0	0
Ltf	3	0	0	2	0	0
Pcolce	0	1	2	1	5	0
1	2	0	0	0	0	3
Rock2	3	0	0	3	0	0
Xpo1	1	0	2	0	0	0
H2-Aa	0	0	4	0	2	3
Prkar2a	1	0	2	1	2	1
Dnm2	1	0	2	1	2	1
Arpc4	1	0	0	0	1	2
Gbp2	0	0	3	2	0	1
S100a10	0	0	0	2	2	1
Copg1	0	0	2	0	1	2
Tjp1	0	0	4	1	0	2
Stab1	0	0	1	2	3	0
Podxl	0	0	1	1	1	4
Psmc3	2	0	0	1	0	0
Itih3	0	3	1	0	4	0
Cat	1	0	0	0	0	0
Kpna4	0	0	1	1	1	1
Calm1	0	0	1	1	2	1
Glul	1	0	0	2	0	1
Hnrnpk	1	0	1	2	0	0
Prdx2	0	1	2	0	1	2
Pygb	3	0	0	1	0	0
Sparc	0	1	1	0	3	2
Pdia3	3	0	1	0	0	0
Hnrnpab	1	0	2	1	0	0
Eif3a	1	0	1	2	0	0
Ca2	0	0	4	0	2	0
Snd1	3	0	0	1	0	0
Npm1	0	0	4	0	1	2
Hmgn1	0	0	5	0	1	1
Pros1	0	0	0	0	4	2
Alox12	4	0	0	0	0	1

Iglc2	1	0	0	0	0	6
Hsp90b1	4	0	1	0	0	0
Plek	7	0	0	0	0	0
Rplp0	1	0	1	1	0	0
Sf3b3	1	0	1	1	0	0
Pcbp1	0	0	1	1	0	0
Ear2	0	0	2	0	2	1
Prep	1	0	0	1	0	1
F11r	0	0	1	2	1	0
Dctn2	1	0	1	1	0	0
Gucy1b1	1	0	1	1	0	0
Psma4	1	0	2	0	1	2
Hnrnpl	0	0	0	1	0	1
Efemp1	0	0	2	0	2	2
Pfkl	1	0	0	4	0	1
Cbx1	0	1	3	0	0	0
Rab1A	0	0	0	3	1	0
Dctn1	2	0	0	2	0	0
Myl12b	0	0	4	0	1	1
Prx	1	0	1	1	0	0
44812	2	0	0	2	0	1
Ntn4	2	0	0	0	0	0
Dnaja2	1	0	0	1	0	0
1	1	0	0	0	0	2
1	1	0	0	0	0	3
Ilk	0	0	1	0	0	2
C1ra	0	0	0	0	3	1
44808	0	0	2	0	2	0
Picalm	0	0	1	0	1	2
Stxbp1	0	0	1	0	1	2
Gars	2	0	0	1	0	0
Cse1l	0	0	0	2	1	1
Slco2a1	0	0	0	1	1	0
Prkacb	2	0	0	0	0	0
Slc3a2	0	0	0	2	0	0
Col5a2	0	0	2	0	1	0
Cotl1	2	0	0	0	0	0
Loxl2	0	0	2	0	1	0
Aebp1	0	0	0	0	1	2
Ecpas	3	0	0	0	0	0
Psmd3	0	0	2	0	0	1
Plcg2	2	0	0	0	0	0
Diaph1	2	0	0	0	0	0
Hnrnpa3	0	0	0	1	0	0
Sf3b1	0	0	0	2	0	0
Aco1	0	0	1	0	0	2
Pnp	0	0	2	0	1	0
Eif2s3x	0	0	2	0	0	0
Sub1	0	0	2	0	0	0
Ubr4	3	0	0	0	0	0
Gpx4	2	0	0	0	0	0
Prcp	0	0	0	2	0	0
Pde5a	2	0	0	0	0	0
Alyref	0	0	2	0	0	0
Unc13d	2	0	0	0	0	0
