# Supplementary material for: Endothelial senescence mediates hypoxia-induced vascular remodeling by modulating PDGFB expression
Source: Front Med (Lausanne). 2022 Sep 20;9:908639. doi: 10.3389/fmed.2022.908639 (PMC9530050; doi:10.3389/fmed.2022.908639)
Supplement: Supplementary file 10 [file Data_Sheet_7.PDF]

Biological Process	PValue	Fold Enrichment	Genes
cell-substrate junction assembly	0.006948554	277.1586207	FN1, **TLN1**
positive regulation of integrin-mediated signaling pathway	4.07253E-09	230.9655172	LAMB2, FLNA, **LAMB1**, LAMC1, NID1
hemidesmosome assembly	0.008332666	230.9655172	**LAMA3**, LAMC1
positive regulation of muscle cell differentiation	8.72976E-07	197.9704433	LAMB2, **LAMB1**, LAMC1, NID1
morphogenesis of a polarized epithelium	0.011095313	173.2241379	LAMA5, **LAMA3**
basement membrane assembly	0.012473852	153.9770115	LAMB2, **LAMB1**
tissue development	8.16745E-14	138.5793103	LAMA5, LAMB2, FASN, **LAMA3**, **LAMB1**, LAMC1, AGRN, HSPG2
integrin activation	0.015225373	125.9811912	FN1, **TLN1**
cell morphogenesis involved in differentiation	0.016598359	115.4827586	LAMB2, **MYH9**
actin filament capping	0.026157712	72.93647913	SPTAN1, SPTBN1
monocyte differentiation	0.027515998	69.28965517	FASN, **MYH9**
neuromuscular junction development	2.68222E-05	65.99014778	COL4A1, LAMB2, **TNC**, AGRN
positive regulation of chemotaxis	0.031579902	60.25187406	FN1, **THBS1**
substrate adhesion-dependent cell spreading	1.21483E-06	59.73246136	LAMA5, LAMB2, FN1, **LAMB1**, LAMC1
positive regulation of fibroblast migration	0.032930894	57.74137931	ACTR3, **THBS1**
plasma membrane organization	0.035627429	53.29973475	AGRN, SPTBN1
regulation of cell adhesion	7.10251E-05	47.78596908	LAMA5, **LAMA4**, **LAMA3**, **TNC**
endodermal cell differentiation	0.03965864	47.78596908	**LAMA3**, FN1
regulation of embryonic development	0.001786291	46.19310345	LAMA5, **LAMA4**, **LAMA3**
peptide cross-linking	0.046341242	40.75862069	FN1, **THBS1**
positive regulation of cell adhesion	0.000135501	38.49425287	LAMB2, **LAMB1**, LAMC1, NID1
blood vessel morphogenesis	0.050329256	37.45386766	COL4A1, **THBS1**
positive regulation of filopodium assembly	0.050329256	37.45386766	ACTR3, AGRN
receptor clustering	0.051655016	36.46823956	FLNA, AGRN
regulation of synapse organization	0.051655016	36.46823956	VCP, AGRN
actin cytoskeleton reorganization	0.003689029	31.97984085	FLNA, **MYH9**, SPTAN1
animal organ morphogenesis	6.75585E-08	30.89347683	LAMA5, LAMB2, **LAMA3**, **LAMB1**, LAMC1, AGRN, HSPG2
positive regulation of cell-substrate adhesion	0.062197043	30.12593703	NID1, **THBS1**
mRNA transcription from RNA polymerase II promoter	0.06481484	28.87068966	ANXA2, FLNA
regulation of cell migration	2.36569E-05	28.39739966	LAMA5, **LAMA4**, **LAMA3**, **TNC**, FLNA
integrin-mediated signaling pathway	0.000436483	25.90267483	LAMA5, **LAMA3**, FN1, **TLN1**
positive regulation of protein localization to plasma membrane	0.075215741	24.74630542	ACTR3, SPTBN1
negative regulation of cell adhesion	0.075215741	24.74630542	**TNC**, **LAMB1**
response to mechanical stimulus	0.085505053	21.65301724	**TNC**, **THBS1**
chondrocyte differentiation	0.089335011	20.68347916	TGFBI, HSPG2
odontogenesis of dentin-containing tooth	0.090608223	20.37931034	LAMA5, **TNC**
synapse organization	0.0931495	19.79704433	LAMB2, FLNA
extracellular matrix organization	0.000117316	18.82871064	COL4A1, FN1, TGFBI, NID1, HSPG2
angiogenesis	2.19296E-06	17.07843613	ANXA2, COL4A1, FN1, FLNA, **MYH9**, TGFBI, HSPG2
protein localization to plasma membrane	0.00155839	16.69630245	LAMA5, ANXA2, FLNA, SPTBN1
cell adhesion	3.41567E-13	16.1138733	LGALS3BP, LAMA5, LAMB2, **LAMA4**, **LAMA3**, **TNC**, FN1, **LAMB1**, LAMC1, NID1, **THBS1**, **MYH9**, TGFBI, **TLN1**
neuron projection development	0.001902394	15.57070903	LAMB2, **TNC**, **LAMB1**, LAMC1
axon guidance	0.002222643	14.74247982	LAMA5, LAMB2, DPYSL2, **LAMA3**
cell migration	5.75309E-05	13.63075184	LAMA5, LAMB2, **LAMA3**, **LAMB1**, LAMC1, **THBS1**
cell-cell adhesion	0.002879681	13.45430198	LAMA5, **LAMA3**, **MYH9**, **TLN1**
regulation of cell shape	0.019835054	13.32493369	FN1, MSN, **MYH9**
actin cytoskeleton organization	0.030872248	10.4984326	FLNA, SPTAN1, SPTBN1
cilium assembly	0.037736647	9.405835544	ACTR3, LAMA5, FLNA
positive regulation of protein phosphorylation	0.0519613	7.873824451	C3, ANXA2, AGRN
positive regulation of cell migration	0.0519613	7.873824451	FN1, **LAMB1**, **THBS1**
brain development	0.055150062	7.614247821	COL4A1, DPYSL2, HSPG2
